# Access to germasiloxanes and alkynylgermanes mediated by earth-abundant species

**DOI:** 10.1038/s41598-023-32172-9

**Published:** 2023-04-06

**Authors:** Hanna Stachowiak-Dłużyńska, Krzysztof Kuciński, Konstancja Broniarz, Ewelina Szafoni, Marcin Gruszczyński, Dariusz Lewandowski, Giuseppe Consiglio, Grzegorz Hreczycho

**Affiliations:** 1 Faculty of Chemistry, Adam Mickiewicz University, Uniwersytetu Poznanskiego St. 8, 61-614 Poznan, Poland; 2 Dipartimento di Scienze Chimiche, Università degli studi di Catania, viale A. Doria 6, 95125 Catania, Italy

**Keywords:** Chemistry, Catalysis, Environmental chemistry, Green chemistry, Chemical synthesis

## Abstract

The reactions between silanols or terminal acetylenes with alkynylgermanes have been accomplished using potassium bis(trimethylsilyl)amide as the catalyst. This strategy has provided an entry point into various organogermanes including germasiloxanes and alkynylgermanes. Remarkably, not only KHMDS but also simple bases such as KOH can serve as efficient catalysts in this process.

## Introduction

**Figure 1:**
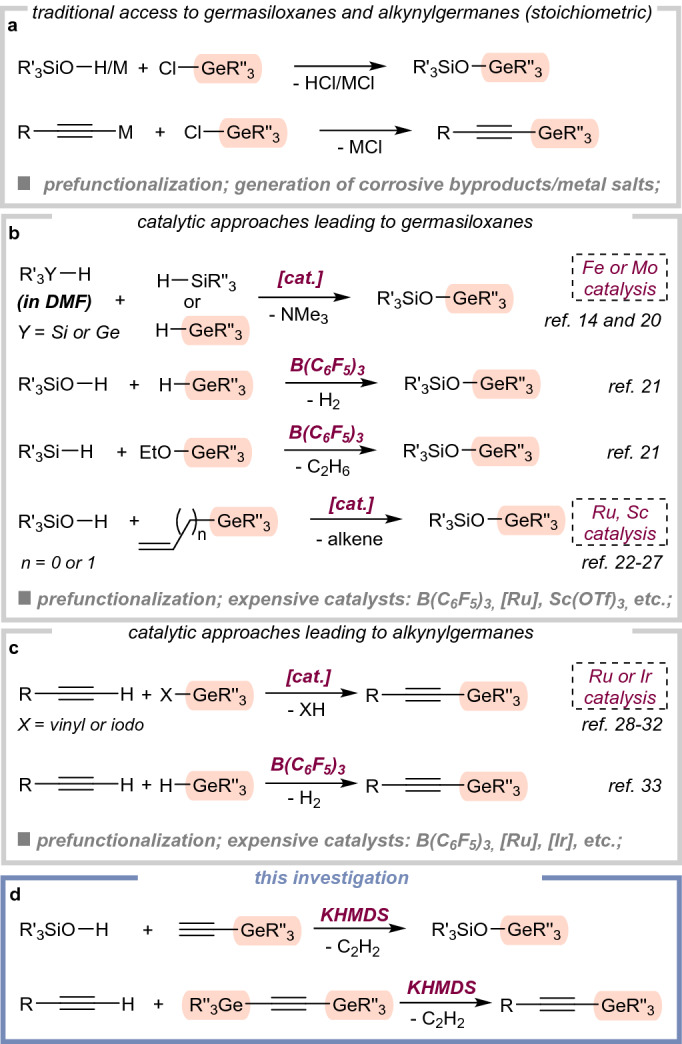
Context of the investigation.

Organogermanium compounds are much less studied than their silicon counterparts, but very recently, an impetus to design new synthetic routes to various organogermanes was witnessed and significant contributions were reported^1–11^. That is not merely a scientific curiosity but primarily, the unique features of germanium compounds are the leading cause of this trend. Moreover, due to their high stability and low toxicity, they can be considered very useful reagents in the synthesis of complex organic molecules^4,12^. The germanium analogues of siloxanes with Ge–O–Si fragments attract considerable attention owing to their high refractive index, low dielectric constant, and biocompatible properties^13–15^. This may lead to completely new materials with different properties than their silicon analogs^16^. There are known several available reaction manifolds to forge Ge–O–Si moieties (Fig. 1). They can be readily accessed via well-developed stoichiometric methods. Here, the germasiloxanes are formed by the condensation of chlorogermanes (or aminogermanes) with silanols or metal silanolates^17,18^. Moreover, there is also a known reaction between germoxanes and silyl azides^19^. Because of the inconvenient nature of these processes (e.g., high moisture sensitivity of substrates, generation of corrosive or explosive byproducts, etc.), researchers have tried to develop catalytic alternatives. These methods can be generally divided into approaches involving siloxymethylamines^20^ (or germyl intermediates^14^), as well as the reaction of various organosilicons with different germylation agents via dehydrogenative^21^, dealkylative^21^, and dealkenative^22–27^ coupling reactions. Despite several advantages, other features of these processes, especially the need for an expensive catalyst (e.g., [Ru_3_(CO)_12_], Sc(OTf)_3_, B(C_6_F_5_)_3_, etc*.*) dramatically reduce their potential. On the other hand, unlike alkynylsilanes, alkynylgermanes have just begun to meet the criteria of useful reagents in organic synthesis. Here, the synthetic arsenal for the formation of *sp* C–Ge bonds mainly relies on the stoichiometric reaction between moisture-sensitive halogermanes with metal acetylides or the use of expensive transition metal complexes (Ru—vinylgermanes/alkynylgermanes^28–30^, Ir—chlorogermanes/iodo-germanes^31,32^). Very recently, an outstanding B(C_6_F_5_)_3_-catalyzed cross-dehydrogenative germylation of terminal alkynes with triethylgermanium hydride was reported by the Schoenebeck group^33^. Considering the drawbacks of previously established methods (e.g., expensive catalysts, the limited availability of hydrogermanes, etc.), it would be fascinating and yet challenging to develop a novel catalytic approach in a more green and sustainable manner. Finally, terminal alkynes can be C–germylated by using 1-trimethylsilyl-2-trimethylgermylacetylene molecule under basic conditions^34^. To the best of our knowledge, there is only one example of such an atypical synthetic strategy. Notably, this strategy is not without its own disadvantages, including the use of fluoride reagents and expensive crown ethers. Moreover, the authors have reported only two products, with low selectivity and yields (less than 50%).

Sustainable and eco-friendly synthetic approaches proceeded by the main-group species have gained recent significant attention^35–44^. Thus, we sought the method leading to versatile germanium compounds that blends the high selectivity of TM-mediated approaches with the practicality of a base-promoted protocol. Based on our recent success in activating silylacetylenes under sustainable catalysis^45–49^, we reasoned that an appropriate catalytic manifold could provide an efficient platform to generate diversified libraries of organogermanes. In this communication, we report on the catalytic O– and *sp* C–germylation by using potassium bis(trimethylsilylamide) as the catalyst (Fig. 1d).

## Results and discussion

**Figure 2:**
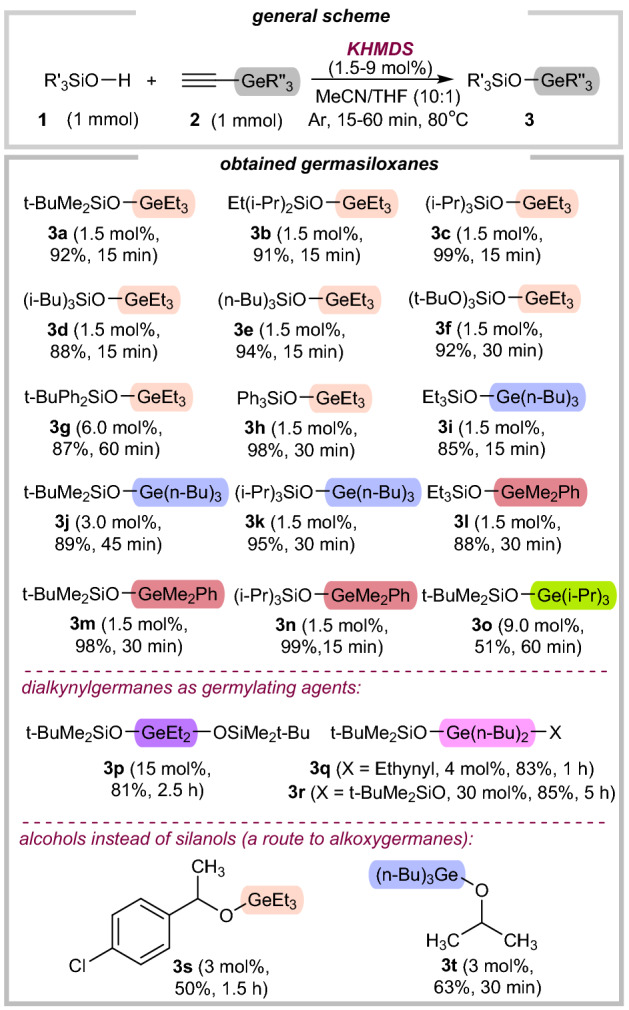
Substrate scope for O-germylation of various silanols.

Our optimization studies (for details, see Table S1 in SI) revealed that utilizing triethyl(ethynyl)germane (**2a**) as the germylating agent, and KHMDS as the catalyst led to the corresponding germasiloxane **3a**. Using a mixture of acetonitrile and tetrahydrofuran as the medium (*v/v* 10:1), this main-group catalytic combination afforded the desired product in 92% yield. Particularly noteworthy is a very good conversion of silanols in the presence of potassium hydroxide (for details, see Table S2 in SI). However, we decided to continue our work with potassium bis(trimethylsilylamide), due to better conversions and yields of final products. The catalyst-free attempt was also carried out and proved the essential role of the main-group catalysis and confirmed no leaching of the alkali species from the glassware, which could act as potential co-catalysts^50,51^. The reaction can be also performed under an air atmosphere but gave inferior results. Figure 2 demonstrates a product scope for alkynylgermanes coupling with silanols using KHMDS in MeCN/THF mixture. The desired germasiloxanes (**3a**-**3n**) were obtained in each case with a very good isolated yield (85–99%). Conversely, in the presence of bulky isopropyl substituents at the germanium atom, the lower conversion and isolated yield were observed (**3o**, 51%).

We were particularly delighted that dialkynylgermanes could be converted to germasiloxanes (**3p**-**3r**). In the case of dibutyldiethynylgermane, the reaction can selectively proceed to both mono- and disubstituted products (**3q** and **3r**). This should be treated as another advantage of this germylation method over previously known approaches due to the possibility of further modifications of the untouched ethynyl group (**3q**). Encouraged by these results, we then investigated the use of alcohols instead of silanols. We were pleased to find that 1-(4-chlorophenyl)ethan-1-ol and isopropanol were successfully germylated under standard conditions (Fig. 2), leading to products **3s** and **3t** in moderate yields (50–63%). Unfortunately, our attempts to perform analogous S– and N–germylation failed. Benzenethiol and aniline were not reactive even under forcing conditions.

**Figure 3:**
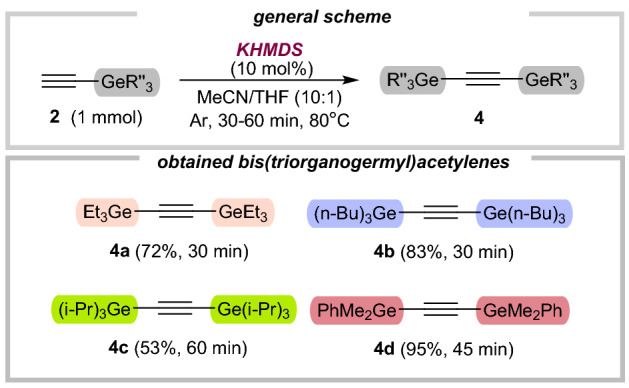
Substrate scope for C–germylation of alkynylgermanes.

Intrigued by the high efficiency and chemoselectivity of the transformation, we next pursued the development of further applications of our catalytic system. Encouragingly, this strategy enabled the germylation of terminal alkynes. We started to explore the substrate scope with respect to alkynylgermanes, particularly to their self-metathesis reaction (Fig. 3).

**Figure 4:**
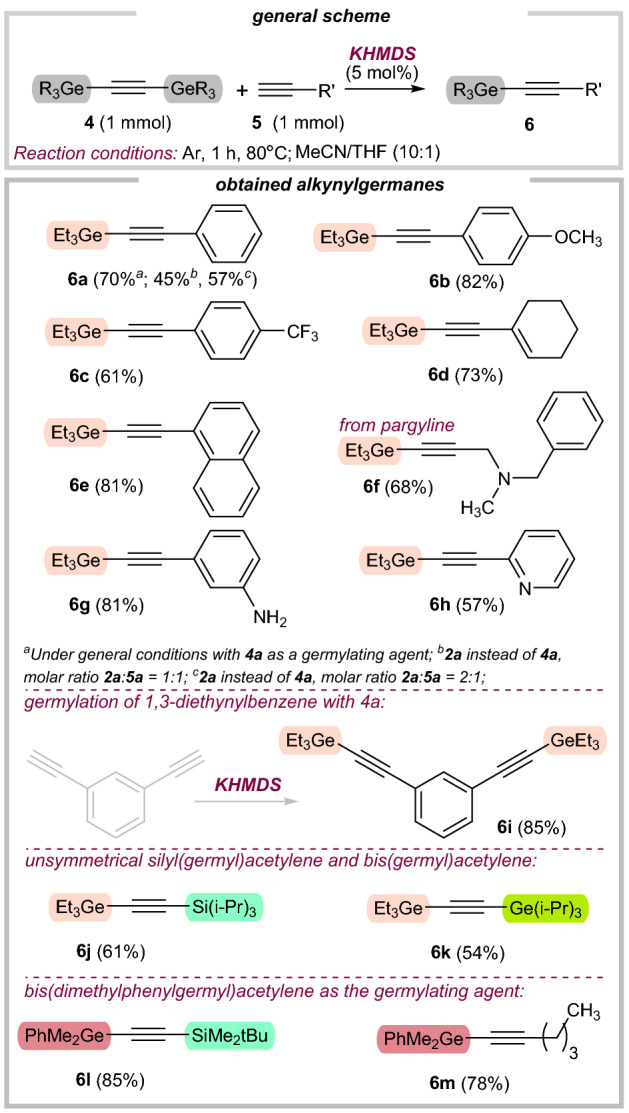
Substrate scope for C–germylation of terminal alkynes.

Thus, except for bulky triisopropyl(ethynyl)germane (**4c**; 53%), the reaction smoothly proceeded with remaining alkynylgermanes in good to excellent yields (72–95%). Guided by our previous studies^45,48,49^, we explored the versatility of our dealkynative coupling of **4a** (or **4d**) employing a representative set of terminal alkynes (Fig. 4). Although the conversion of phenylacetylene (**5a**) was also seen with triethyl(ethynyl)germane (**2a**), we later found that isolated yields were significantly lower than for **4a** (Fig. 4; footnotes *a*, *b*, and *c*). Notably, the excess of **4a** can be recovered **via** distillation. However, all actions should be performed exclusively under an argon atmosphere. Otherwise, significant amounts of digermoxane byproduct are formed, thus impeding the isolation process (as well as recovery of **4a)**. With suitable reaction conditions established (Fig. 4; general conditions), we turned toward the examination of the substrate scope. Coupling reactions of phenylacetylenes substituted with an electron-donating group **5b** and electron-withdrawing group **5c** provided the corresponding alkynylgermanes **6b** and **6c** in good yields (61–82%), and the parent phenylacetylene **5a** reacted equally well (70%). Next, commercial ene-yne derivative **5d** also participated effectively in this reaction (**6d**, 73% yield), while preserving the ene-functionality untouched. Encouraged by these results, we then investigated the use of *N*-containing acetylenes **5f**-**5h**, which are biorelevant scaffolds. Our strategy enabled the germylation of one pharmaceutical—pargyline (**5f**), known as an inhibitor of monoamine-oxidase-B (68%). Importantly, the amine moiety in **5g** remained during the reaction, confirming again the high chemoselectivity of our approach. Next, we sought to obtain germylated diyne, which can be subsequently used in polymer chemistry. Using our catalytic system, product **6i** was afforded (85% yield). Next, unsymmetrical silyl(germyl)- and bis(germyl)acetylenes were obtained in moderate yields (**6j** and **6k**, 54–61%). It should be noted, that in these particular cases, the products were selectively obtained only for bulky substituted silane (**6j**) or germane (**6k**). Otherwise, we observed the mixture of symmetrical and unsymmetrical bis-substituted acetylenes. Finally, we also tested our methodology on bis(dimethylphenylgermyl)acetylene (**4d**), providing the desired products **6l** and **6m** in very good yields (78–85%). All these examples highlight both the electronic generality of this method and its tolerance for typically existing organic motifs, showcasing the unique robustness and versatility of our strategy.

**Figure 5:**
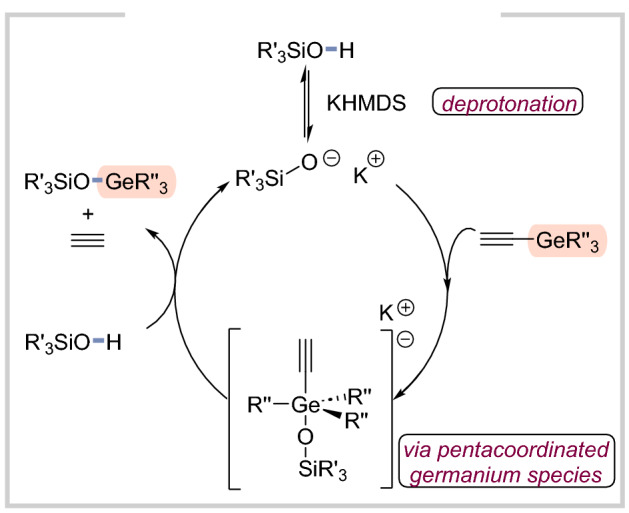
Plausible mechanism for the O–germylation.

**Figure 6:**
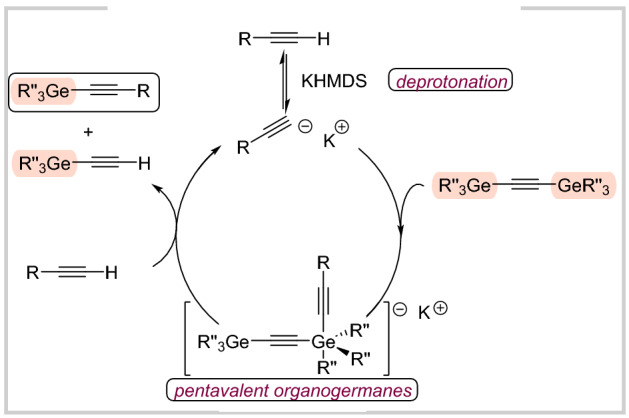
Plausible mechanism for the *sp* C–germylation.

Based on our experimental results (for details, see SI) and our previous developments in the case of analogues alkynylsilanes, a plausible catalytic cycle is presented for the O–germylation (Fig. 5; the mechanism for *sp* C–germylation is shown in Fig. 6). Please note (for details please see SI), that both silanol and triethylethynylgermane can undergo the deprotonation. However, we still suggest that “the silanol activation pathway” is dominant for the O–germylation considering the expected higher acidity of silanols compared to alkynylgermanes. In the case of *sp* C–germylation, ^1^H NMR analysis confirmed a deprotonation step (no signal for the acetylenic proton, for details please see SI). Subsequently, it is suggested that formed acetylide reacts with bis(triorganogermyl)acetylene to generate pentacoordinated germanium intermediates. This is followed by the addition of another alkyne molecule with simultaneous liberation of the desired product and triorganogermylacetylene. Notably, the latter can also serve as a germylating agent or undergo a self-metathesis reaction, which finally leads to the evolution of gaseous acetylene in both scenarios. In general, all the previous protocols for the base-catalyzed reactions of analogous silylacetylenes were assuming the intermediacy of hypervalent species. In our specific case, it is also the most probable pathway. Thus, a plausible catalytic cycle is presented in Fig. 6.

## Conclusions

In summary, we have reported on a very efficient protocol for catalytic O–H and *sp* C–H germylation of silanols and terminal alkynes in the presence of main-group species. Here, a commercially available KHMDS enabled a dealkynative coupling with ample scope. Considering the combination of desirable features (e.g., operational simplicity, high chemoselectivity, benign reaction conditions, low cost of the reagents, and their commercial availability, etc.), we believe this reaction system offers new perspectives for the synthesis of valuable organogermanium compounds in a sustainable and green manner. Furthermore, we anticipate that the general design principle utilizing alkynyl-substituted metalloids will lead to new reactions that are difficult to achieve with traditional transformations. Thus, further studies on the scope and synthetic application of this methodology are currently underway in our laboratory.

## Methods

General Information: Air- and moisture-sensitive reactions were carried out under an argon atmosphere using standard Schlenk techniques or a glove box. Solvents used for all experiments were purchased from Honeyweel or Sigma Aldrich (Merck), dried over calcium hydride (CaH_2_), and purified by distillation. Tetrahydrofurane was additionally dried over sodium with benzophenone system. All alkali metal compounds (lithium bis(trimethylsilyl)amide, sodium bis(trimethylsilyl)amide, potassium bis(trimethylsilyl)amide, lithium tert-butoxide, sodium tert-butoxide, potassium tert-butoxide, lithium hydroxide, sodium hydroxide, and potassium hydroxide) were purchased in the solid state from Sigma Aldrich (Merck) or StanLab. Additionally, potassium bis(trimethylsilyl)amide was also purchased as a solution in THF from Sigma Aldrich (Merck). Commercially available silanols (e.g.*,* tert-butyldimethylsilanol, tert-butyldiphenylsilanol, tris(tert-butoxy)silanol, triethylsilanol, triisopropylsilanol, triphenylsilanol, etc.) were purchased from Sigma Aldrich (Merck) or AmBeed and used as received. Non-commercially available silanols were prepared by hydrolysis of corresponding chlorosilanes (e.g., chlorotriisobutylsilane, chlorotributylsilane, etc.). Terminal alkynes (e.g., phenylacetylene, 4-ethynylanisole, 4-ethynyl-α,α,α-trifluorotoluene, 1-ethynylcyclohexene, *N*-methyl-*N*-propargylbenzylamine, etc.) were purchased from Sigma-Aldrich (Merck) and used as received. Alkynylgermanes (e.g., triethyl(ethynyl)germane, tributyl(ethynyl)germane, ethynyldimethyl(phenyl)germane, ethynyltriisopropylgermane, etc*.*) were synthesized from corresponding chlorogermanes by well-known procedure using ethynylmagnesium bromide solution in THF (Grignard reagent). The progress of reactions (conversion of alkynylgermane, silanol, alcohol, or alkyne) was monitored by GC chromatography using Bruker Scion 460-GC and Agilent 5977B GC/MSD with Agilent 8860 GC System. The structures of products were determined by NMR spectroscopy and MS spectrometry. The ^1^H NMR (400 or 600 MHz), ^13^C NMR (101 or 151 MHz) and ^29^Si NMR (79 or 119 MHz) spectra were recorded on Bruker Avance III HD NanoBay spectrometer, using chloroform-d1 (CDCl_3_) or benzene-d6 (C_6_D_6_) as the solvents. Deuterated solvents were purchased from respectively Deutero GmbH (CDCl_3_ 99.6 atom% D) and Sigma Aldrich (Merck) (C_6_D_6_ 99.8 atom% D) and used as received.

## Supplementary Information

Supplementary Information.

## Data Availability

The datasets used and/or analyzed during the current study available from the corresponding author on reasonable request.
