# Supplementary material for: Access to germasiloxanes and alkynylgermanes mediated by earth-abundant species
Source: Sci Rep. 2023 Apr 6;13:5618. doi: 10.1038/s41598-023-32172-9 (PMC10079689; doi:10.1038/s41598-023-32172-9)
Supplement: Supplementary file 1 — Supplementary Information. [file 41598_2023_32172_MOESM1_ESM.pdf]

SUPPORTING INFORMATION

For

Access to Germasiloxanes and Alkynylgermanes Mediated by Earth-Abundant Species

Hanna Stachowiak-Dłużyńska,[a] Krzysztof Kuciński,[a*] Konstancja Broniarz,[a] Ewelina Szafoni,[a] Marcin Gruszczyński, [a] Dariusz Lewandowski,[a] Giuseppe Consiglio,[b] and Grzegorz Hreczycho[a*]

[a] Faculty of Chemistry, Adam Mickiewicz University in Poznań, Uniwersytetu Poznańskiego 8, 61-614 Poznan (Poland).
[b] Dipartimento di Scienze Chimiche, Università degli studi di Catania, viale A. Doria 6, I-95125 Catania (Italy).

e-mail:
kucinski.k@amu.edu.pl; g.h@amu.edu.pl

CONTENT:

1. GENERAL INFORMATION..2
2. OPTIMIZATION STUDIES..3
3. EXPERIMENTAL PROCEDURES..4
4. CHARACTERIZATION DATA FOR ALL PRODUCTS..7
5. SPECTRA FOR ALL PRODUCTS..20
6. REFERENCES..95

GENERAL INFORMATION

Air- and moisture-sensitive reactions were carried out under an argon atmosphere using standard Schlenk techniques or a glove box. Solvents used for all experiments were purchased from Honeyweel or Sigma Aldrich (Merck), dried over calcium hydride (CaH_2), and purified by distillation. Tetrahydrofurane was additionally dried over sodium with benzophenone system. All alkali metal compounds (lithium bis(trimethylsilyl)amide, sodium bis(trimethylsilyl)amide, potassium bis(trimethylsilyl)amide, lithium tert-butoxide, sodium tert-butoxide, potassium tert-butoxide, lithium hydroxide, sodium hydroxide, and potassium hydroxide) were purchased in the solid state from Sigma Aldrich (Merck) or StanLab. Additionally, potassium bis(trimethylsilyl)amide was also purchased as a solution in THF from Sigma Aldrich (Merck). Commercially available silanols (*e.g.,* tert-butyldimethylsilanol, tert-butyldiphenylsilanol, tris(tert-butoxy)silanol, triethylsilanol, triisopropylsilanol, triphenylsilanol, *etc.*) were purchased from Sigma Aldrich (Merck) or AmBeed and used as received. Non-commercially available silanols were prepared by hydrolysis of corresponding chlorosilanes (*e.g.,* chlorotriisobutylsilane, chlorotributylsilane, *etc.*). Terminal alkynes (*e.g.,* phenylacetylene, 4-ethynylanisole, 4-ethynyl-α,α,α-trifluorotoluene, 1-ethynylcyclohexene, N-methyl-N-propargylbenzylamine, *etc.*) were purchased from Sigma-Aldrich (Merck) and used as received. Alkynylgermanes (*e.g.,* triethyl(ethynyl)germane, tributyl(ethynyl)germane, ethynyldimethyl(phenyl)germane, ethynyltriisopropylgermane, *etc.*) were synthesized from corresponding chlorogermanes by well-known procedure using ethynylmagnesium bromide solution in THF (Grignard reagent). The progress of reactions (conversion of alkynylgermane, silanol, alcohol, or alkyne) was monitored by GC chromatography using Bruker Scion 460-GC and Agilent 5977B GC/MSD with Agilent 8860 GC System. The structures of products were determined by NMR spectroscopy and MS spectrometry. The ^{1}H NMR (400 or 600 MHz), ^{13}C NMR (101 or 151 MHz) and ^{29}Si NMR (79 or 119 MHz) spectra were recorded on Bruker Avance III HD NanoBay spectrometer, using chloroform-d1 ($CDCl_3$) or benzene-d6 (C_6D_6) as the solvents. Deuterated solvents were purchased from respectively Deutero GmbH ($CDCl_3$ 99.6 atom% D) and Sigma Aldrich (Merck) (C_6D_6 99.8 atom% D) and used as received.

OPTIMIZATION STUDIES

Table S1. Optimization process for dealkynative coupling utilizing **2a**

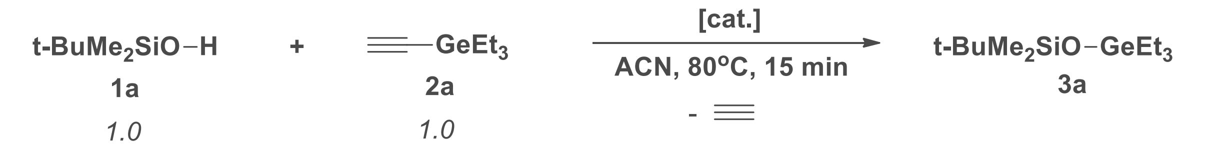

No.	Catalyst	Cat. load. [mol%]	Conv. of 2a [%][a]
1	LiHMDS	1.5	traces
2	NaHMDS	1.5	3
3	KHMDS	1.0	56
4		1.5	95
5			0[b]
6			0[c]
7			**97[d] (92[e])**
8		2.0	95
9	LiOtBu	2.0	traces
10	NaOtBu	2.0	52
11	KOtBu	1.0	61
12		1.5	89
13		2.0	92
14	LiOH	3.0	1
15	NaOH	3.0	47
16	KOH	2.0	56
17		3.0	95
18		4.0	95

[a] Determined by GC; [b] in the presence of THF instead of ACN; [c] in the presence of 2-Me-THF instead of ACN; [d] in the presence of ACN/THF (10:1) instead of ACN; [e] isolated yield.

Table S2. Comparison of KHMDS and KOH in the preparation of germasiloxanes

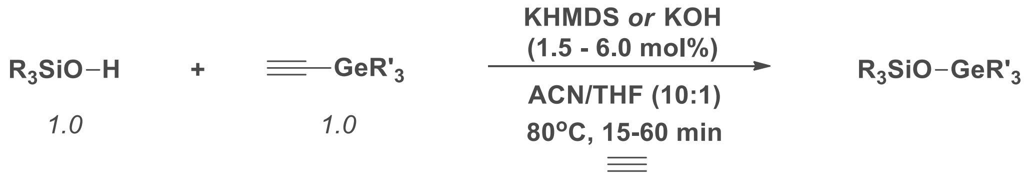

No.	Ethynylgermane	Silanol	Catalyst	Cat. load. [mol%]	Time [min]	Isolated yield [%]
1	≡—$GeEt_3$	$Ph_3SiO{-}H$	KOH	3.0	15	86
2			KHMDS	1.5	20	98
3	≡—$Ge(n\text{-}Bu)_3$	$Et_3SiO{-}H$	KOH	6.0	15	80
4			KHMDS	1.5	15	85
5		$t\text{-}BuMe_2SiO{-}H$	KOH	6.0	60	79
6			KHMDS	3.0	40	89
7	≡—$GeMe_2Ph$	$Et_3SiO{-}H$	KOH	3.0	15	82
8			KHMDS	1.5	20	88
9		$t\text{-}BuMe_2SiO{-}H$	KOH	6.0	15	84
10			KHMDS	1.5	20	98

EXPERIMENTAL PROCEDURES

*General procedure for the preparation of germasiloxanes using alkynylgermanes (**3a-3o**)*

To a 50 mL Schlenk tube equipped with a magnetic stirring bar and a septum were added respectively: 1 mL of acetonitrile, 100 µL of tetrahydrofuran, 1 mmol of alkynylgermane (*e.g.,* triethyl(ethynyl)germane, tributyl(ethynyl)germane, *etc.*), 1 mmol of silanol (*e.g.,* triethylsilanol, triphenylsilanol, *etc.*) and 1.5 mol% (3 mol% for product **3j**, 6 mol% for **3g**, and 9 mol% for **3o**) of KHMDS in THF (C_m = 1.0 M) under argon atmosphere. The reaction mixture was heated at 80°C and the progress was controlled by gas chromatography (GC). After the corresponding time for each reaction, solvents and all volatile residues were evaporated under reduced pressure on a rotary evaporator. Next, the crude products were separated by trap-to-trap distillation or an extraction with Et_2O/H_2O mixture. Finally, all the desired products were characterized by ^{1}H, ^{13}C, and ^{29}Si NMR, and on GC-MS.

*General procedure for the preparation of germasiloxane **3q***

To a 50 mL Schlenk tube equipped with a magnetic stirring bar and a septum were added respectively: 1 mL of acetonitrile, 100 µL of tetrahydrofuran, 1 mmol of dibutyldiethynylgermane, 1 mmol of tert-butyldimethylsilanol and 4 mol% of KHMDS in THF (C_m = 1.0 M) under argon atmosphere. The reaction mixture was heated at 80°C and the progress was controlled by gas chromatography (GC). After corresponding time (1 h) solvents

and all volatile residues were evaporated under reduced pressure on a rotary evaporator. Next, the crude product was separated by trap-to-trap distillation to give corresponding product **3q**. Finally, the desired product was characterized by ^{1}H, ^{13}C, and ^{29}Si NMR, and on GC-MS.

*General procedure for the preparation of germasiloxanes using dialkynylgermanes (**3p** and **3r**)*

To a 50 mL Schlenk tube equipped with a magnetic stirring bar and a septum were added respectively: 1 mL of acetonitrile, 100 µL of tetrahydrofuran, 1 mmol of diethyldiethynylgermane or dibutyldiethynylgermane, 3 mmol of tert-butyldimethylsilanol and appropriate amount (15 mol% for product **3p**, and 30 mol% for **3r**) of KHMDS in THF (C_m = 1.0 M) under argon atmosphere. The reaction mixture was heated at 80°C and the progress was controlled by gas chromatography (GC). After corresponding time for each reaction, solvents and all volatile residues were evaporated under reduced pressure on a rotary evaporator. Next, the crude products were separated by trap-to-trap distillation or an extraction with Et_2O/H_2O mixture. Finally, all the desired products were characterized by ^{1}H, ^{13}C, and ^{29}Si NMR, and on GC-MS.

*General procedure for the preparation of alkoxygermanes (**3s-3t**)*

To a 50 mL Schlenk tube equipped with a magnetic stirring bar and a septum were added respectively: 1 mL of acetonitrile, 100 µL of tetrahydrofuran, 1 mmol of triethyl(ethynyl)germane or tributyl(ethynyl)germane, 1 mmol of respectively 1-(4-chlorophenyl)ethanol or propan-2-ol, and 3 mol% of KHMDS in THF (C_m = 1.0 M) under argon atmosphere. The reaction mixture was heated at 80°C and the progress was controlled by gas chromatography (GC). After corresponding time for each reaction, solvents and all volatile residues were evaporated under reduced pressure on a rotary evaporator. Next, the crude products were separated by trap-to-trap distillation. Finally, both desired products were characterized by ^{1}H and ^{13}C NMR and on GC-MS.

*General procedure for the preparation of bis(triorganogermyl)acetylenes (**4a-4d**)*

To a 50 mL Schlenk tube equipped with a magnetic stirring bar and a septum were added respectively: 1 mL of acetonitrile, 100 µL of tetrahydrofuran, 1 mmol of alkynylgermane (*e.g.,* triethyl(ethynyl)germane, tributyl(ethynyl)germane, *etc.*) and 10 mol% of KHMDS in THF (C_m = 1.0 M) under argon atmosphere. The reaction mixture was heated at 80°C and the progress was controlled by gas chromatography (GC). After corresponding time for each reaction, solvents and all volatile residues were evaporated under reduced pressure on a rotary evaporator. Next, the crude products were separated by trap-to-trap distillation or an extraction with Et_2O/H_2O mixture. Finally, all the desired products were characterized by ^{1}H and ^{13}C NMR, and on GC-MS.

*General procedure for C-germylation of terminal alkynes (**6a-6m**)*

To a 50 mL Schlenk tube equipped with a magnetic stirring bar and a septum were added respectively: 1 mL of acetonitrile, 100 µL of tetrahydrofuran, 1 mmol of bis(triethylgermyl)acetylene (**4a**) or bis(dimethylphenylgermyl)acetylene (**4d**), 1 mmol of alkyne (*e.g.* phenylacetylene, 4-ethynylanisole, *etc.*) and 5 mol% of KHMDS in THF (C_m = 1.0 M) under argon atmosphere. The reaction mixture was heated at 80°C and the

progress was controlled by a gas chromatography (GC). After the corresponding time (1 h), solvents and all volatile residues were evaporated under reduced pressure on a rotary evaporator. Next, the crude products were separated by trap-to-trap distillation. Finally, all the desired products were characterized by ^{1}H, ^{13}C, and ^{29}Si NMR, and on GC-MS.

*KHMDS-catalyzed O-germylation of **1a** in the presence of TEMPO*

To a 50 mL Schlenk tube equipped with a magnetic stirring bar and a septum were added respectively: 1 mL of acetonitrile, 100 µL of tetrahydrofuran, 1 mmol of triethyl(ethynyl)germane (**2a**), 1 mmol of tert-butyldimethylsilanol (**1a**), 1 mmol of TEMPO, and 1.5 mol% of KHMDS in THF (C_m = 1.0 M) under argon atmosphere. The reaction mixture was heated at 80°C and the progress was controlled by gas chromatography (GC). After the specified time (15 min) solvents and all volatile residues were evaporated under reduced pressure on a rotary evaporator. Next, the crude product was separated by trap-to-trap distillation to give corresponding product **3a** (89%).

CHARACTERIZATION DATA FOR ALL PRODUCTS

Tert-butyldimethyl((triethylgermyl)oxy)silane (3a)

t-BuMe$_2$SiO—GeEt$_3$

Tert-butyldimethyl((triethylgermyl)oxy)silane was obtained as oil in 92% yield. The title compound was known in the literature and all spectroscopic data are in agreement.[1]

^{1}H NMR (400 MHz, CDCl$_3$) δ (ppm) = 0.00 (s, 6H), 0.84-0.95 (m, 15H), 1.04-1.12 (m, 9H).

^{13}C NMR (101 MHz, CDCl$_3$) δ (ppm) = -2.2, 7.8, 8.4, 18.7, 26.0.

^{29}Si NMR (79 MHz, CDCl$_3$) δ (ppm) = 9.2.

EI-MS m/z (rel. int.): 263 (4%, [M-Et]$^+$), 235 (100, [M-*t*-Bu]$^+$), 221 (11), 207 (68), 193 (8), 179 (27), 163 (9), 149 (42), 133 (10), 119 (10), 103 (10), 89 (6), 75 (11), 59 (24).

Ethyldiisopropyl((triethylgermyl)oxy)silane (3b)

Et(i-Pr)$_2$SiO—GeEt$_3$

Ethyldiisopropyl((triethylgermyl)oxy)silane was obtained as oil in 91% yield. The title compound was previously unknown.

^{1}H NMR (400 MHz, CDCl$_3$) δ (ppm) = 0.54 (q, J = 7.9 Hz, 2H), 0.80-0.96 (m, 9H), 0.97-1.04 (m, 14H), 1.05-1.14 (m, 9H).

^{13}C NMR (101 MHz, CDCl$_3$) δ (ppm) = 4.8, 7.5, 7.9, 8.7, 13.8, 17.8.

^{29}Si NMR (79 MHz, CDCl$_3$) δ (ppm) = 6.9.

EI-MS m/z (rel. int.): 291 (10%, [M-Et]$^+$), 277 (100, [M-*i*Pr]$^+$), 263 (5), 249 (39), 235 (22), 221 (22), 207 (20), 193 (16), 177 (19), 163 (15), 149 (26), 133 (21), 119 (21), 103 (15), 89 (7), 75 (6), 61 (6).

Triisopropyl((triethylgermyl)oxy)silane (3c)

(i-Pr)$_3$SiO—GeEt$_3$

Triisopropyl((triethylgermyl)oxy)silane was obtained as oil in 99% yield. The title compound was known in the literature and all spectroscopic data are in agreement.[2]

^{1}H NMR (400 MHz, CDCl$_3$) δ (ppm) = 0.84-0.95 (m, 9H), 0.96-1.03 (m, 18H), 1.04-1.10 (m, 9H).

^{13}C NMR (101 MHz, CDCl$_3$) δ (ppm) = 7.9, 8.8, 13.5, 18.2.

^{29}Si NMR (79 MHz, CDCl$_3$) δ (ppm) = 4.9.

EI-MS m/z (rel. int.): 305 (6%, [M-Et]$^+$), 291 (100, [M-*i*Pr]$^+$), 263 (24), 249 (27), 235 (10), 221 (18), 207 (12), 191 (16), 177 (9), 161 (14), 149 (16), 133 (18), 119 (14), 103 (16), 89 (3), 75 (5), 59 (4).

Triisobutyl((triethylgermyl)oxy)silane (3d)

$(i\text{-}Bu)_3SiO-GeEt_3$

Triisobutyl((triethylgermyl)oxy)silane was obtained as oil in 88% yield. The title compound was known in the literature and all spectroscopic data are in agreement.[1]

^{1}H NMR (400 MHz, $CDCl_3$) δ (ppm) = 0.50 (d, J = 6.8 Hz, 6H), 0.84-0.91 (m, 6H), 0.93 (d, J = 6.6 Hz, 18H), 1.03-1.09 (m, 9H), 1.73-1.86 (m, 3H).

^{13}C NMR (101 MHz, $CDCl_3$) δ (ppm) = 7.9, 8.7, 24.5, 26.6, 28.6.

^{29}Si NMR (79 MHz, $CDCl_3$) δ (ppm) = 4.6.

EI-MS m/z (rel. int.): 319 (27%, $[M\text{-}i\text{Bu}]^+$), 291 (6), 263 (65), 235 (9), 207 (100), 177 (22), 159 (7), 149 (23), 133 (13), 119 (14), 103 (13), 89 (2), 75 (2), 57 (4).

Tributyl((triethylgermyl)oxy)silane (3e)

$(n\text{-}Bu)_3SiO-GeEt_3$

Tributyl((triethylgermyl)oxy)silane was obtained as oil in 94% yield. The title compound was previously unknown.

^{1}H NMR (400 MHz, $CDCl_3$) δ (ppm) = 0.43-0.54 (m, 6H), 0.80-0.94 (m, 15H), 1.00-1.11 (m, 9H), 1.22-1.39 (m, 12H).

^{13}C NMR (101 MHz, $CDCl_3$) δ (ppm) = 7.8, 8.5, 13.8, 16.2, 25.8, 26.8.

^{29}Si NMR (79 MHz, $CDCl_3$) δ (ppm) = 6.6.

EI-MS m/z (rel. int.): 347 (4%, $[M\text{-}Et]^+$), 319 (96), 291 (95), 263 (100), 233 (16), 207 (94), 177 (35), 159 (9), 149 (34), 133 (12), 121 (20), 103 (22), 87 (2), 75 (4), 55 (7).

Tri-tert-butyl(triethylgermyl)orthosilicate (3f)

$(t\text{-}BuO)_3SiO-GeEt_3$

Tri-tert-butyl(triethylgermyl)orthosilicate was obtained as oil in 92% yield. The title compound was previously unknown.

^{1}H NMR (400 MHz, $CDCl_3$) δ (ppm) = 0.90-0.98 (m, 6H), 1.05-1.12 (m, 9H), 1.28 (s, 27H).

^{13}C NMR (101 MHz, $CDCl_3$) δ (ppm) = 7.9, 8.1, 31.4, 71.6.

^{29}Si NMR (79 MHz, $CDCl_3$) δ (ppm) = -94.4.

EI-MS m/z (rel. int.): 409 (3%, $[M\text{-}Me]^+$), 395 (2), 297 (9), 267 (11), 239 (34), 227 (100), 209 (10), 181 (16), 169 (20), 151 (11), 119 (10), 79 (5), 57 (43).

Tert-butyldiphenyl((triethylgermyl)oxy)silane (3g)

$t\text{-}BuPh_2SiO{-}GeEt_3$

Tert-butyldiphenyl((triethylgermyl)oxy)silane was obtained as oil in 87% yield. The title compound was previously unknown.

^{1}H NMR (400 MHz, $CDCl_3$) δ (ppm) = 0.85-0.93 (m, 6H), 0.99-1.06 (m, 9H), 1.08 (s, 9H), 7.34-7.43 (m, 6H), 7.67-7.74 (m, 4H).

^{13}C NMR (101 MHz, $CDCl_3$) δ (ppm) = 7.9, 8.7, 19.7, 27.1, 127.3, 128.9, 135.3, 137.8.

^{29}Si NMR (79 MHz, $CDCl_3$) δ (ppm) = -12.6.

EI-MS m/z (rel. int.): 387 (2%, $[M\text{-}Et]^+$), 359 (100), 331 (22), 301 (11), 273 (17), 253 (4), 225 (15), 197 (17), 183 (82), 151 (30), 135 (8), 121 (10), 105 (24), 78 (10), 57 (23).

Triphenyl((triethylgermyl)oxy)silane (3h)

$Ph_3SiO{-}GeEt_3$

Triphenyl((triethylgermyl)oxy)silane was obtained as oil in 98% yield. The title compound was previously unknown.

^{1}H NMR (400 MHz, $CDCl_3$) δ (ppm) = 0.88-0.98 (m, 6H), 1.02-1.11 (m, 9H), 7.37-7.48 (m, 9H), 7.59-7.73 (m, 6H).

^{13}C NMR (101 MHz, $CDCl_3$) δ (ppm) = 7.9, 8.7, 127.6, 129.3, 135.1, 138.1.

^{29}Si NMR (79 MHz, $CDCl_3$) δ (ppm) = -21.7.

EI-MS m/z (rel. int.): 407 (100%, $[M\text{-}Et]^+$), 379 (8), 349 (4), 329 (3), 301 (7), 273 (14), 259 (41), 229 (16), 199 (6), 189 (16), 183 (27), 151 (23), 136 (4), 105 (12), 77 (4).

Triethyl((tributylgermyl)oxy)silane (3i)

$Et_3SiO{-}Ge(n\text{-}Bu)_3$

Triethyl((tributylgermyl)oxy)silane was obtained as oil in 85% yield. The title compound was known in the literature,[3] but it was characterized for the first time.

^{1}H NMR (400 MHz, $CDCl_3$) δ (ppm) = 0.42-0.59 (m, 6H), 0.84-1.02 (m, 24H), 1.30-1.48 (m, 12H).

^{13}C NMR (101 MHz, $CDCl_3$) δ (ppm) = 7.1, 7.1, 13.7, 17.2, 26.2.

^{29}Si NMR (79 MHz, $CDCl_3$) δ (ppm) = 8.9.

EI-MS m/z (rel. int.): 347 (86%, $[M\text{-}Et]^+$), 319 (26), 291 (93), 263 (64), 235 (88), 207 (83), 177 (100), 149 (86), 133 (18), 119 (38), 103 (27), 87 (11), 75 (12), 57 (16).

Tert-butyldimethyl((tributylgermyl)oxy)silane (3j)

t-BuMe$_2$SiO—Ge(n-Bu)$_3$

Tert-butyldimethyl((tributylgermyl)oxy)silane was obtained as oil in 89% yield. The title compound was known in the literature and all spectroscopic data are in agreement.[4]

^{1}H NMR (600 MHz, CDCl$_3$) δ (ppm) = 0.00 (s, 6H), 0.84-0.90 (m, 15H), 0.91-0.95 (m, 9H), 1.32-1.46 (m, 12H).

^{13}C NMR (151 MHz, CDCl$_3$) δ (ppm) = -2.1, 13.7, 17.1, 18.7, 26.0, 26.2, 26.2.

^{29}Si NMR (119 MHz, CDCl$_3$) δ (ppm) = 8.9.

EI-MS m/z (rel. int.): 319 (65%, [M-*t*-Bu]$^+$), 263 (82), 235 (7), 221 (10), 207 (88), 179 (18), 163 (30), 149 (100), 133 (9), 103 (6), 89 (10), 73 (13), 59 (23).

Triisopropyl((tributylgermyl)oxy)silane (3k)

(i-Pr)$_3$SiO—Ge(n-Bu)$_3$

Triisopropyl((tributylgermyl)oxy)silane was obtained as oil in 95% yield. The title compound was known in the literature and all spectroscopic data are in agreement.[4]

^{1}H NMR (400 MHz, CDCl$_3$) δ (ppm) = 0.82-0.95 (m, 18H), 0.96-1.08 (m, 18H), 1.28-1.46 (m, 12H).

^{13}C NMR (101 MHz, CDCl$_3$) δ (ppm) = 13.5, 13.7, 17.4, 18.2, 26.2, 26.3.

^{29}Si NMR (79 MHz, CDCl$_3$) δ (ppm) = 4.8.

EI-MS m/z (rel. int.): 375 (64%, [M-*i*-Pr]$^+$), 319 (100), 305 (8), 275 (7), 263 (68), 249 (8), 235 (8), 219 (18), 205 (28), 189 (11), 177 (18), 163 (33), 149 (19), 131 (34), 119 (20), 103 (11), 89 (74), 75 (9), 55 (10).

((Dimethyl(phenyl)germyl)oxy)triethylsilane (3l)

Et$_3$SiO—GeMe$_2$Ph

((Dimethyl(phenyl)germyl)oxy)triethylsilane was obtained as oil in 88% yield. The title compound was previously unknown.

^{1}H NMR (400 MHz, CDCl$_3$) δ (ppm) = 0.53 (q, J = 7.9 Hz, 6H), 0.65 (s, 6H), 0.96 (t, J = 7.9 Hz, 9H), 7.40-7.46 (m, 3H), 7.57-7.63 (m, 2H).

^{13}C NMR (101 MHz, CDCl$_3$) δ (ppm) = 1.4, 7.0, 7.0, 128.1, 129.3, 132.5, 141.2.

^{29}Si NMR (79 MHz, CDCl$_3$) δ (ppm) = 11.8.

EI-MS m/z (rel. int.): 297 (2%, [M-Me]$^+$), 283 (100, [M-Et]$^+$), 255 (34), 227 (13), 181 (11), 167 (12), 149 (20), 135 (12), 121 (12), 107 (9), 89 (5).

Tert-butyl((dimethyl(phenyl)germyl)oxy)dimethylsilane (3m)

t-BuMe$_2$SiO—GeMe$_2$Ph

Tert-butyl((dimethyl(phenyl)germyl)oxy)dimethylsilane was obtained as oil in 98% yield. The title compound was previously unknown.

^{1}H NMR (400 MHz, $CDCl_3$) δ (ppm) = 0.01 (s, 6H), 0.63 (s, 6H), 0.91 (s, 9H), 7.39-7.47 (m, 3H), 7.56-7.62 (m, 2H).

^{13}C NMR (101 MHz, $CDCl_3$) δ (ppm) = -2.2, 1.4, 18.6, 26.0, 128.1, 129.3, 132.5, 141.2.

^{29}Si NMR (79 MHz, $CDCl_3$) δ (ppm) = 11.6.

EI-MS m/z (rel. int.): 255 (100%, [M-*t*-Bu]$^+$), 239 (5), 181 (35), 151 (11), 135 (18), 119 (12), 73 (11).

((Dimethyl(phenyl)germyl)oxy)triisopropylsilane (3n)

(i-Pr)$_3$SiO—GeMe$_2$Ph

((Dimethyl(phenyl)germyl)oxy)triisopropylsilane was obtained as oil in 99% yield. The title compound was previously unknown.

^{1}H NMR (400 MHz, $CDCl_3$) δ (ppm) = 0.64 (s, 6H), 0.91-1.05 (m, 21H), 7.37-7.42 (m, 3H), 7.55-7.61 (m, 2H).

^{13}C NMR (101 MHz, $CDCl_3$) δ (ppm) = 1.6, 13.3, 18.2, 128.1, 129.2, 132.5, 141.3.

^{29}Si NMR (79 MHz, $CDCl_3$) δ (ppm) = 7.4.

EI-MS m/z (rel. int.): 311 (100%, [M-*i*-Pr]$^+$), 283 (9), 269 (30), 255 (10), 241 (21), 225 (10), 181 (35), 163 (15), 149 (21), 135 (20), 121 (18), 105 (15), 75 (11).

Tert-butyldimethyl((triisopropylgermyl)oxy)silane (3o)

t-BuMe$_2$SiO—Ge(i-Pr)$_3$

Tert-butyldimethyl((triisopropylgermyl)oxy)silane was obtained as oil in 51% yield. The title compound was previously unknown.

^{1}H NMR (400 MHz, $CDCl_3$) δ (ppm) = 0.01 (s, 6H), 0.88 (s, 9H), 1.14 (d, J = 7.4 Hz, 18H) 1.32-1.42 (m, 3H).

^{13}C NMR (101 MHz, $CDCl_3$) δ (ppm) = -1.9, 17.3, 18.8, 19.1, 26.2.

^{29}Si NMR (79 MHz, $CDCl_3$) δ (ppm) = 7.6.

EI-MS m/z (rel. int.): 291 (18%, [M-*i*-Pr]$^+$), 277 (100, [M-*t*-Bu]$^+$), 249 (48), 235 (28), 221 (10), 207 (29), 191 (15), 179 (14), 163 (16), 149 (52), 133 (11), 73 (17), 59 (19).

Bis(tert-butyldimethylsiloxy)diethylgermane (3p)

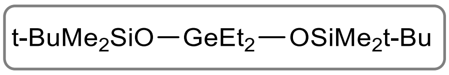

Bis(tert-butyldimethylsiloxy)diethylgermane was obtained as oil in 81% yield. The title compound was known in the literature and all spectroscopic data are in agreement.[1]

^{1}<u>H NMR</u> (400 MHz, $CDCl_3$) δ (ppm) = 0.02 (s, 12H), 0.85-0.90 (m, 18H), 0.93-1.01 (m, 4H) 1.06-1.13 (m, 6H).

^{13}<u>C NMR</u> (101 MHz, $CDCl_3$) δ (ppm) = -2.3, 7.2, 11.6, 18.6, 25.9.

^{29}<u>Si NMR</u> (79 MHz, $CDCl_3$) δ (ppm) = 10.8.

<u>EI-MS m/z (rel. int.)</u>: 337 (26%, $[M\text{-}t\text{-}Bu]^+$), 223 (16), 147 (100), 133 (99), 119 (72), 105 (19), 89 (17), 73 (25), 57 (27).

Tert-butyl((dibutyl(ethynyl)germyl)oxy)dimethylsilane (3q)

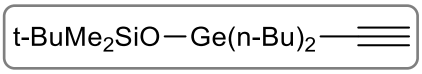

Tert-butyl((dibutyl(ethynyl)germyl)oxy)dimethylsilane was obtained as oil in 83% yield. The title compound was previously unknown.

^{1}<u>H NMR</u> (400 MHz, $CDCl_3$) δ (ppm) = 0.04 (s, 6H), 0.85-0.88 (m, 9H), 0.91 (t, J = 7.3 Hz, 6H), 0.99-1.06 (m, 4H), 1.32-1.43 (m, 4H), 1.44-1.55 (m, 4H), 2.38 (s, 1H).

^{13}<u>C NMR</u> (101 MHz, $CDCl_3$) δ (ppm) = -2.5, 13.7, 18.5, 18.6, 25.6, 25.8, 25.9, 88.2, 93.0.

^{29}<u>Si NMR</u> (79 MHz, $CDCl_3$) δ (ppm) = 13.3.

<u>EI-MS m/z (rel. int.)</u>: 287 (30%, $[M\text{-}t\text{-}Bu]^+$), 231 (48), 173 (100), 159 (12), 149 (34), 83 (63), 75 (22), 57 (20).

Bis(tert-butyldimethylsiloxy)dibutylgermane (3r)

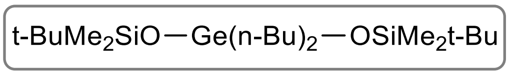

Bis(tert-butyldimethylsiloxy)dibutylgermane was obtained as oil in 85% yield. The title compound was previously unknown.

^{1}<u>H NMR</u> (400 MHz, $CDCl_3$) δ (ppm) = 0.02 (s, 12H), 0.85-0.88 (m, 18H), 0.90 (t, J = 7.3 Hz, 6H), 0.93-1.00 (m, 4H), 1.30-1.40 (m, 4H), 1.42-1.52 (m, 4H).

^{13}<u>C NMR</u> (101 MHz, $CDCl_3$) δ (ppm) = -2.3, 13.7, 18.6, 19.7, 25.4, 25.7, 25.9.

^{29}<u>Si NMR</u> (79 MHz, $CDCl_3$) δ (ppm) = 10.7.

<u>EI-MS m/z (rel. int.)</u>: 393 (21%, $[M\text{-}t\text{-}Bu]^+$), 223 (16), 203 (33), 161 (15), 147 (100), 133 (36), 105 (18), 89 (15), 73 (13), 57 (17).

(1-(4-Chlorophenyl)ethoxy)triethylgermane (3s)

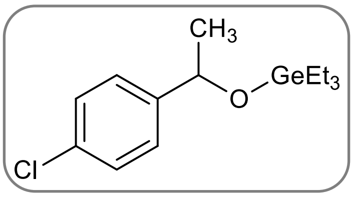

(1-(4-Chlorophenyl)ethoxy)triethylgermane was obtained as oil in 50% yield. The title compound was previously unknown.

^{1}H NMR (400 MHz, C_6D_6) δ (ppm) = 0.57-0.70 (m, 6H), 0.84 (t, J = 7.9 Hz, 9H), 1.27 (d, J = 6.3 Hz, 3H), 4.47 (q, J = 6.3 Hz, 1H), 7.05 (s, 4H).

^{13}C NMR (101 MHz, C_6D_6) δ (ppm) = 6.6, 7.7, 28.0, 71.3, 126.8, 128.2, 132.1, 147.4.

EI-MS m/z (rel. int.): 301 (60%, $[M-Me]^+$), 287 (59), 243 (37), 215 (8), 161 (34), 149 (39), 139 (62), 133 (51), 121 (26), 111 (14), 103 (100), 91 (18), 77 (45), 63 (6), 51 (14).

Tributyl(isopropoxy)germane (3t)

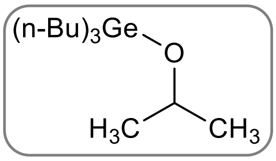

Tributyl(isopropoxy)germane was obtained as oil in 63% yield. The title compound was known in the literature.[5]

^{1}H NMR (400 MHz, C_6D_6) δ (ppm) = 0.78-0.87 (m, 15H), 1.15 (d, J = 6.0 Hz, 5H), 1.22-1.30 (m, 6H), 1.33-1.44 (m, 6H), 3.75-3.90 (m, 1H).

^{13}C NMR (101 MHz, C_6D_6) δ (ppm) = 13.6, 15.3, 26.3, 26.4, 26.8, 65.5.

EI-MS m/z (rel. int.): 247 (74%, $[M\text{-}n\text{-}Bu]^+$), 189 (100), 133 (89), 103 (13), 89 (31), 55 (13).

1,2-Bis(triethylgermyl)ethyne (4a)

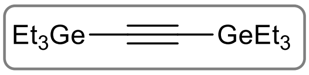

1,2-Bis(triethylgermyl)ethyne was obtained as oil in 72% yield. The title compound was known in the literature and all spectroscopic data are in agreement.[6]

^{1}H NMR (400 MHz, $CDCl_3$) δ (ppm) = 0.79-0.88 (m, 12H), 1.05-1.13 (m, 18H).

^{13}C NMR (101 MHz, $CDCl_3$) δ (ppm) = 5.9, 9.0, 110.8.

EI-MS m/z (rel. int.): 315 (100%, $[M-Et]^+$), 287 (18), 259 (13), 229 (15), 201 (14), 173 (32), 157 (12), 129 (25), 115 (10), 101 (24), 75 (4).

1,2-Bis(tributylgermyl)ethyne (4b)

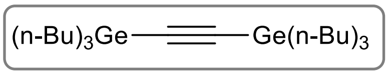

1,2-Bis(tributylgermyl)ethyne was obtained as oil in 83% yield. The title compound was previously unknown.

^{1}H NMR (400 MHz, $CDCl_3$) δ (ppm) = 0.81-0.89 (m, 12H), 0.92 (t, J = 7.2 Hz, 18H), 1.32-1.40 (m, 12H), 1.41-1.52 (m, 12H).

^{13}C NMR (101 MHz, $CDCl_3$) δ (ppm) = 13.8, 14.3, 26.1, 27.5, 111.6.

EI-MS m/z (rel. int.): 455 (100%, $[M\text{-}n\text{-}Bu]^+$), 399 (16), 343 (10), 287 (20), 245 (18), 229 (37), 189 (62), 171 (63), 157 (32), 143 (14), 129 (40), 101 (18), 89 (19), 55 (40).

1,2-Bis(triisopropylgermyl)ethyne (4c)

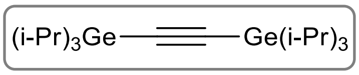

1,2-Bis(triisopropylgermyl)ethyne was obtained as oil in 53% yield. The title compound was previously unknown.

^{1}H NMR (400 MHz, $CDCl_3$) δ (ppm) = 1.19 (d, J = 7.3 Hz, 36H), 1.31-1.44 (m, 6H).

^{13}C NMR (101 MHz, $CDCl_3$) δ (ppm) = 14.7, 19.9, 110.0.

EI-MS m/z (rel. int.): 385 (100%, $[M\text{-}i\text{-}Pr]^+$), 343 (12), 299 (4), 257 (11), 215 (13), 171 (21), 159 (6), 141 (5), 115 (4).

1,2-Bis(dimethyl(phenyl)germyl)ethyne (4d)

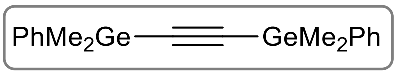

1,2-Bis(dimethyl(phenyl)germyl)ethyne was obtained as oil in 95% yield. The title compound was previously unknown.

^{1}H NMR (400 MHz, $CDCl_3$) δ (ppm) = 0.60-0.69 (m, 12H), 7.37-7.46 (m, 6H), 7.60-7.71 (m, 4H).

^{13}C NMR (101 MHz, $CDCl_3$) δ (ppm) = -0.8, 111.4, 128.2, 128.9, 133.2, 138.9.

EI-MS m/z (rel. int.): 384 (4%, $[M]^+$), 369 (100, $[M\text{-}Me]^+$), 267 (15), 243 (11), 205 (10), 189 (9), 177 (38), 151 (25), 115 (5), 77 (12), 51 (10).

Triethyl(phenylethynyl)germane (6a)

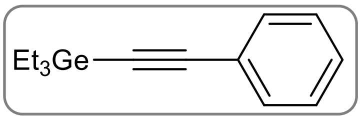

Triethyl(phenylethynyl)germane was obtained as oil in 70% yield. The title compound was known in the literature and all spectroscopic data are in agreement.[7]

^{1}H NMR (400 MHz, C_6D_6) δ (ppm) = 0.89 (q, J = 8.0 Hz, 6H), 1.16 (t, J = 7.9 Hz, 9H), 6.87-6.99 (m, 3H), 7.45-7.55 (m, 2H).

^{13}C NMR (101 MHz, C_6D_6) δ (ppm) = 5.8, 9.0, 91.7, 106.6, 124.1, 127.9, 128.1, 131.9.

EI-MS m/z (rel. int.): 262 (3%, $[M]^+$), 233 (100, [M-Et]), 205 (36), 175 (67), 149 (15), 103 (14).

Triethyl((4-methoxyphenyl)ethynyl)germane (6b)

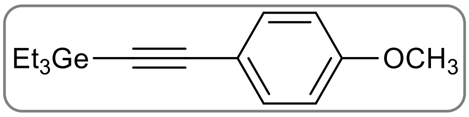

Triethyl((4-methoxyphenyl)ethynyl)germane was obtained as oil in 82% yield. The title compound was known in the literature and all spectroscopic data are in agreement.[8]

^{1}H NMR (400 MHz, C_6D_6) δ (ppm) = 0.91 (q, J = 7.9 Hz, 6H), 1.19 (t, J = 7.9 Hz, 9H), 3.14 (s, 3H), 6.49-6.60 (m, 2H), 7.41-7.51 (m, 2H).

^{13}C NMR (101 MHz, C_6D_6) δ (ppm) = 5.8, 9.0, 54.3, 89.8, 106.6, 113.9, 116.3, 133.4, 159.6.

EI-MS m/z (rel. int.): 292 (15%, $[M]^+$), 263 (100, $[M\text{-}Et]^+$), 235 (31), 205 (40), 190 (8), 181 (12), 132 (10).

Triethyl((4-(trifluoromethyl)phenyl)ethynyl)germane (6c)

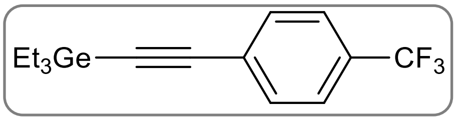

Triethyl((4-(trifluoromethyl)phenyl)ethynyl)germane was obtained as oil in 61% yield. The title compound was previously unknown.

^{1}H NMR (400 MHz, C_6D_6) δ (ppm) = 0.99 (q, J = 7.9 Hz, 6H), 1.26 (t, J = 7.9 Hz, 9H), 7.16-7.24 (m, 2H), 7.32-7.41 (m, 2H).

^{13}C NMR (101 MHz, C_6D_6) δ (ppm) = 5.7, 8.9, 95.2, 104.9, 124.2 (q, J = 272 Hz), 125.0 (q, J = 3.8 Hz), 127.7 (d, J = 24 Hz), 129.48 (q, J = 32.5 Hz), 132.1.

EI-MS m/z (rel. int.): 301 (100%, $[M\text{-}Et]^+$), 273 (30), 243 (59), 170 (7), 151 (27).

(Cyclohex-1-en-1-ylethynyl)triethylgermane (6d)

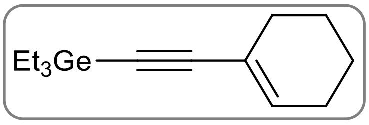

(Cyclohex-1-en-1-ylethynyl)triethylgermane was obtained as oil in 73% yield. The title compound was known in the literature and all spectroscopic data are in agreement.[7]

^{1}H NMR (400 MHz, C_6D_6) δ (ppm) = 0.86 (q, J = 8.0 Hz, 6H), 1.16 (t, J = 7.9 Hz, 9H), 1.22-1.42 (m, 4H), 1.74-1.83 (m, 2H), 2.12-2.25 (m, 2H), 6.13-6.27 (m, 1H).

^{13}C NMR (101 MHz, C_6D_6) δ (ppm) = 5.8, 9.0, 21.4, 22.2, 25.4, 29.6, 88.0, 108.8, 121.5, 134.5.

EI-MS m/z (rel. int.): 266 (4%, $[M]^+$), 237 (100, $[M-Et]^+$), 209 (35), 181 (40), 151 (15), 105 (20), 91 (21), 77 (10).

Triethyl(naphthalen-1-ylethynyl)germane (6e)

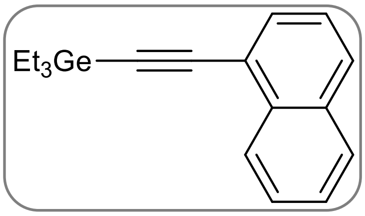

Triethyl(naphthalen-1-ylethynyl)germane was obtained as oil in 81% yield. The title compound was previously unknown.

^{1}H NMR (400 MHz, C_6D_6) δ (ppm) = 0.65-0.75 (m, 6H), 0.94-1.02 (m, 9H), 6.76-6.84 (m, 1H), 6.94-7.02 (m, 1H), 7.10-7.18 (m, 1H), 7.23 (d, J = 8.4 Hz, 1H), 7.31 (d, J = 8.2 Hz, 1H), 7.45-7.50 (m, 1H), 8.50-8.56 (m, 1H).

^{13}C NMR (101 MHz, C_6D_6) δ (ppm) = 5.9, 9.1, 97.1, 104.5, 121.7, 125.1, 126.2, 126.4, 126.7, 128.3, 128.5, 130.7, 133.4, 133.8.

EI-MS m/z (rel. int.): 312 (22%, $[M]^+$), 283 (100), 255 (37), 225 (92), 199 (23), 178 (8), 165 (16), 152 (44), 113 (13), 99 (12).

N-Benzyl-N-methyl-3-(triethylgermyl)prop-2-yn-1-amine (6f)

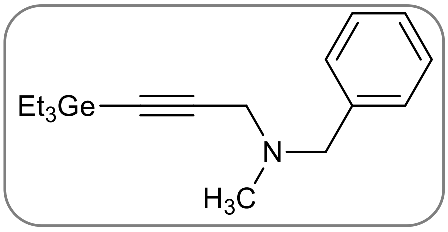

N-Benzyl-N-methyl-3-(triethylgermyl)prop-2-yn-1-amine was obtained as oil in 68% yield. The title compound was known in the literature and all spectroscopic data are in agreement.[7]

^{1}H NMR (400 MHz, C_6D_6) δ (ppm) = 0.64 (q, J = 7.9 Hz, 6H), 0.96 (t, J = 7.9 Hz, 9H), 2.08 (s, 3H), 3.08 (s, 2H), 3.36 (s, 2H), 6.84-6.92 (m, 1H), 6.94-6.97 (m, 2H), 7.17-7.24 (m, 2H).

^{13}C NMR (101 MHz, C_6D_6) δ (ppm) = 5.8, 9.0, 41.5, 46.0, 59.9, 86.2, 101.5, 127.0, 128.2, 129.0, 139.2.

EI-MS m/z (rel. int.): 319 (8%, $[M]^+$), 290 (18, $[M-Et]^+$), 158 (64), 133 (9), 118 (16), 102 (11), 91 (100), 65 (10).

3-((Triethylgermyl)ethynyl)aniline (6g)

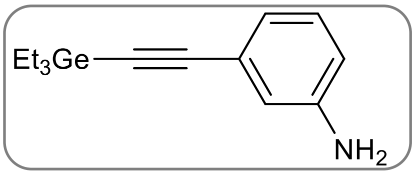

3-((Triethylgermyl)ethynyl)aniline was obtained as oil in 81% yield. The title compound was previously unknown.

^{1}H NMR (400 MHz, C_6D_6) δ (ppm) = 0.60-0.69 (m, 6H), 0.93 (t, J = 7.9 Hz, 9H), 2.44 (s, 2H), 5.87-5.94 (m, 1H), 6.40-6.45 (m, 1H), 6.56-6.63 (m, 1H), 6.75-6.82 (m, 1H).

^{13}C NMR (101 MHz, C_6D_6) δ (ppm) = 5.8, 9.0, 90.5, 107.2, 114.7, 118.0, 122.0, 124.6, 129.0, 146.6.

EI-MS m/z (rel. int.): 277 (32%, $[M]^+$), 248 (100), 220 (46), 190 (81), 173 (8), 162 (17), 117 (15), 95 (19), 89 (12).

2-((Triethylgermyl)ethynyl)pyridine (6h)

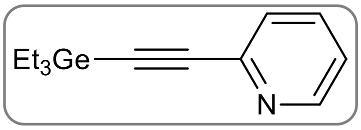

2-((Triethylgermyl)ethynyl)pyridine obtained as oil in 57% yield. The title compound was known in the literature and all spectroscopic data are in agreement.[9]

^{1}H NMR (400 MHz, C_6D_6) δ (ppm) = 0.81-0.89 (m, 6H), 1.13 (t, J = 7.9 Hz, 9H), 6.45 (ddd, J = 7.6, 4.8, 1.2 Hz, 1H), 6.80 (td, J = 7.7, 1.9 Hz, 1H), 7.16-7.22 (m, 1H), 8.31-8.39 (m, 1H).

^{13}C NMR (101 MHz, C_6D_6) δ (ppm) = 5.6, 8.9, 92.3, 106.3, 122.1, 126.9, 135.0, 144.0, 149.9.

EI-MS m/z (rel. int.): 234 (100%, $[M-Et]^+$), 206 (18), 176 (68), 152 (36), 123 (6), 104 (8), 99 (9), 78 (7).

1,3-Bis((triethylgermyl)ethynyl)benzene (6i)

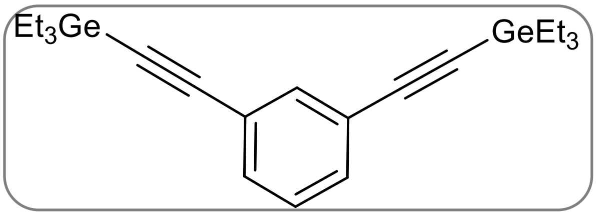

1,3-Bis((triethylgermyl)ethynyl)benzene was obtained as oil in 85% yield. The title compound was previously unknown.

^{1}H NMR (400 MHz, C_6D_6) δ (ppm) = 0.81-0.92 (m, 12H), 1.07-1.20 (m, 18H), 6.75 (t, J = 7.8 Hz, 1H), 7.27-7.35 (m, 2H), 7.85-7.93 (m, 1H).

^{13}C NMR (101 MHz, C_6D_6) δ (ppm) = 6.1, 9.3, 92.9, 105.9, 124.6, 128.6, 131.8, 135.8.

EI-MS m/z (rel. int.): 444 (3%, $[M]^+$), 415 (100, $[M-Et]^+$), 387 (11), 359 (6), 331 (8), 301 (8), 271 (13), 199 (27), 179 (6), 164 (10), 150 (12), 137 (19), 103 (6).

Triisopropyl((triethylgermyl)ethynyl)silane (6j)

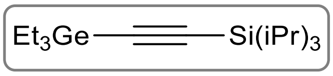

Triisopropyl((triethylgermyl)ethynyl)silane was obtained as oil in 61% yield. The title compound was known in the literature and all spectroscopic data are in agreement.[6]

^{1}H NMR (400 MHz, C_6D_6) δ (ppm) = 0.75-0.88 (m, 6H), 1.04-1.23 (m, 30H).

^{13}C NMR (101 MHz, C_6D_6) δ (ppm) = 6.1, 9.4, 11.6, 18.9, 110.5, 113.6.

^{29}Si NMR (79 MHz, $CDCl_3$) δ (ppm) = -3.1

EI-MS m/z (rel. int.): 313 (25%, $[M-Et]^+$), 299 (100, $[M-i\text{Pr}]^+$), 271 (30), 243 (17), 229 (23), 213 (9), 201 (16), 169 (18), 157 (12), 143 (14), 129 (13), 111 (11), 101 (13), 83 (11), 73 (8), 59 (7).

Triethyl((triisopropylgermyl)ethynyl)germane (6k)

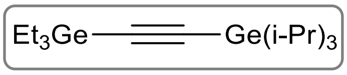

Triethyl((triisopropylgermyl)ethynyl)germane was obtained as oil in 54% yield. The title compound was previously unknown.

^{1}H NMR (400 MHz, C_6D_6) δ (ppm) = 0.81 (q, J = 8.0 Hz, 6H), 1.16 (t, J = 7.9 Hz, 9H), 1.24 (d, J = 7.1 Hz, 18H), 1.29-1.39 (m, 3H).

^{13}C NMR (101 MHz, C_6D_6) δ (ppm) = 5.9, 9.0, 14.7, 19.8, 109.7, 111.4.

EI-MS m/z (rel. int.): 357 (7%, $[M-Et]^+$), 343 (100, $[M-i\text{-Pr}]^+$), 301 (17), 271 (6), 201 (6), 171 (13).

Tert-butyldimethyl((triethylgermyl)ethynyl)silane (6l)

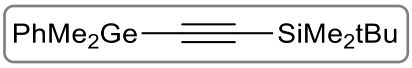

Tert-butyldimethyl((triethylgermyl)ethynyl)silane was obtained as oil in 85% yield. The title compound was previously unknown.

^{1}H NMR (400 MHz, C_6D_6) δ (ppm) = 0.00 (s, 6H), 0.30 (s, 6H), 0.89 (s, 9H), 7.01-7.09 (m, 3H), 7.42-7.51 (m, 2H).

^{13}C NMR (101 MHz, C_6D_6) δ (ppm) = -3.1, 0.2, 27.5, 113.8, 114.0, 129.7, 130.5, 134.6, 140.1.

^{29}Si NMR (79 MHz, $CDCl_3$) δ (ppm) = -9.3.

EI-MS m/z (rel. int.): 320 (4%, $[M]^+$), 305 (9, $[M-Me]^+$), 263 (100, $[M-t\text{Bu}]^+$), 233 (5), 159 (34), 145 (10), 135 (11), 83 (12).

Hex-1-yn-1-yldimethyl(phenyl)germane (6m)

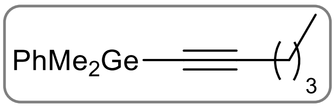

Hex-1-yn-1-yldimethyl(phenyl)germane was obtained as oil in 78% yield. The title compound was previously unknown.

^{1}H NMR (400 MHz, C_6D_6) δ (ppm) = 0.52 (s, 6H), 0.77 (t, J = 7.2 Hz, 3H), 1.27-1.44 (m, 4H), 2.14 (t, J = 7.0 Hz, 2H), 7.18-7.26 (m, 3H), 7.64-7.70 (m, 2H).

^{13}C NMR (101 MHz, C_6D_6) δ (ppm) = -0.8, 13.7, 19.9, 22.2, 31.2, 81.9, 107.9, 128.5, 129.1, 133.5, 139.7.

EI-MS m/z (rel. int.): 247 (100%, $[M-Me]^+$), 220 (5), 189 (10), 157 (25), 151 (12), 115 (14), 89 (8).

SPECTRA FOR ALL PRODUCTS

Tert-butyldimethyl((triethylgermyl)oxy)silane (3a)

t-BuMe_2SiO—GeEt_3

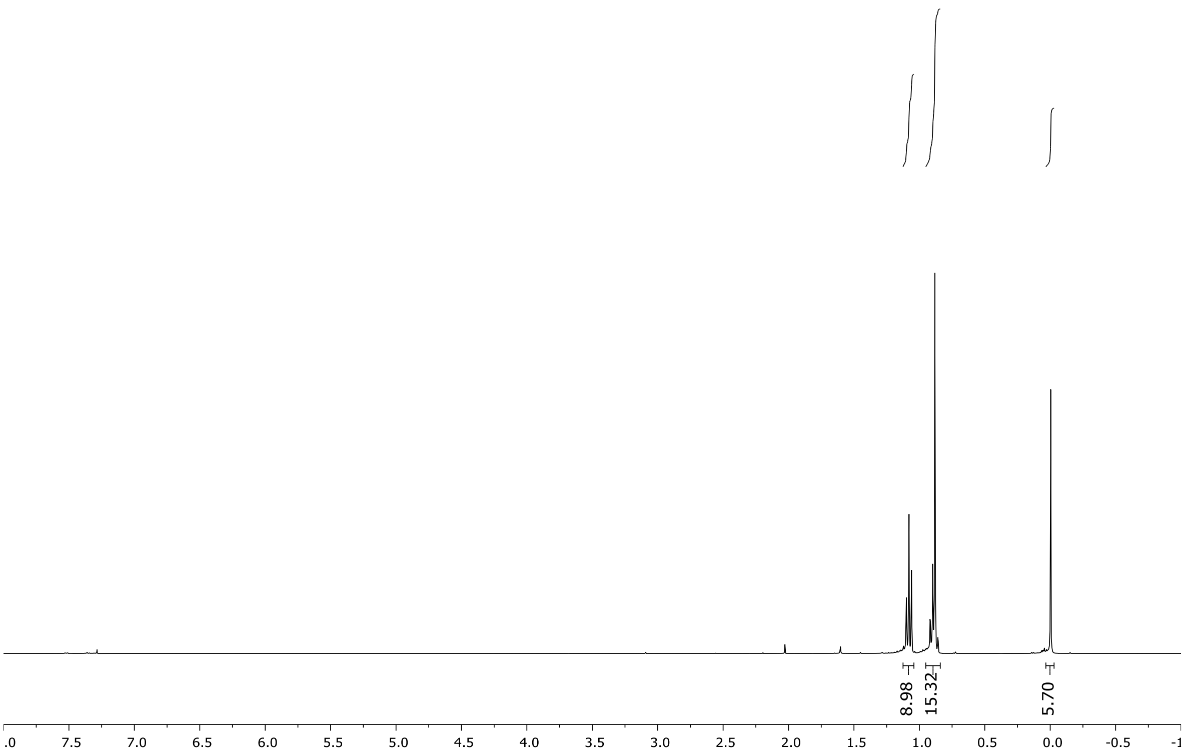

Figure S1. ^{1}H NMR Spectra of tert-butyldimethyl((triethylgermyl)oxy)silane (**3a**)

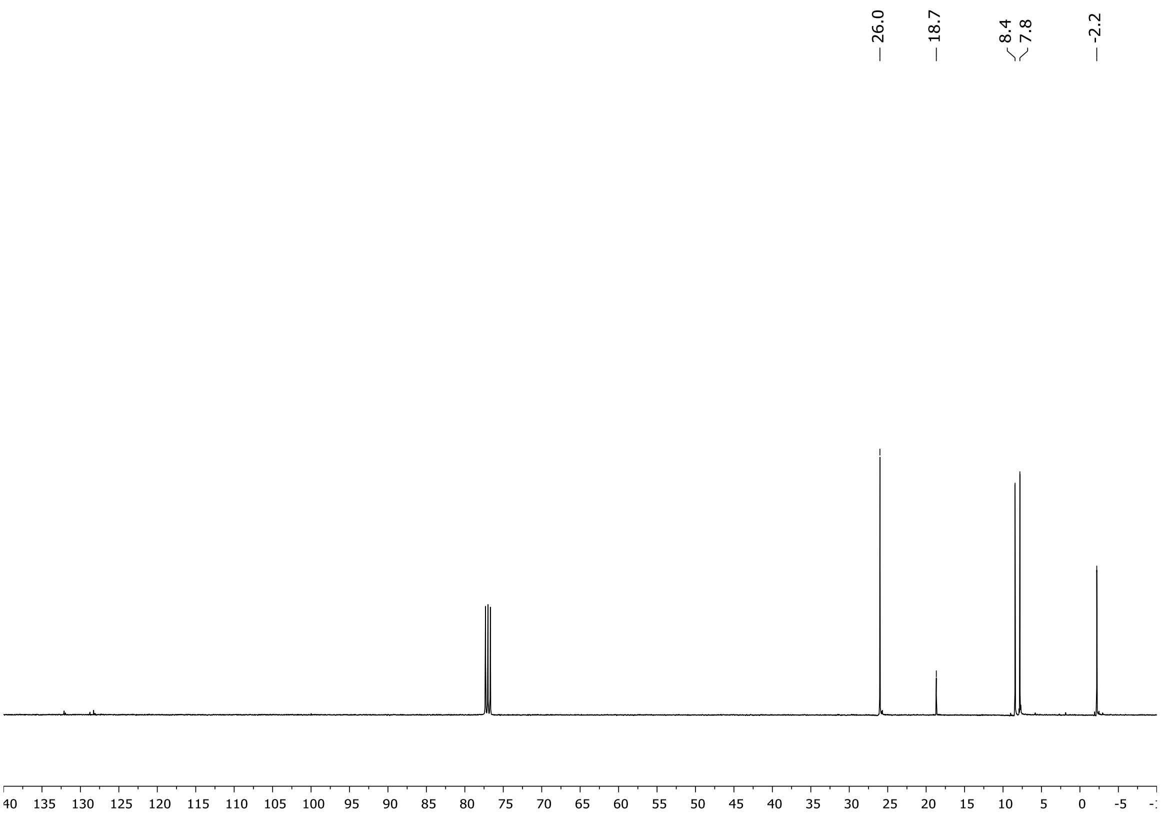

Figure S2. ^{13}C NMR Spectra of tert-butyldimethyl((triethylgermyl)oxy)silane (**3a**)

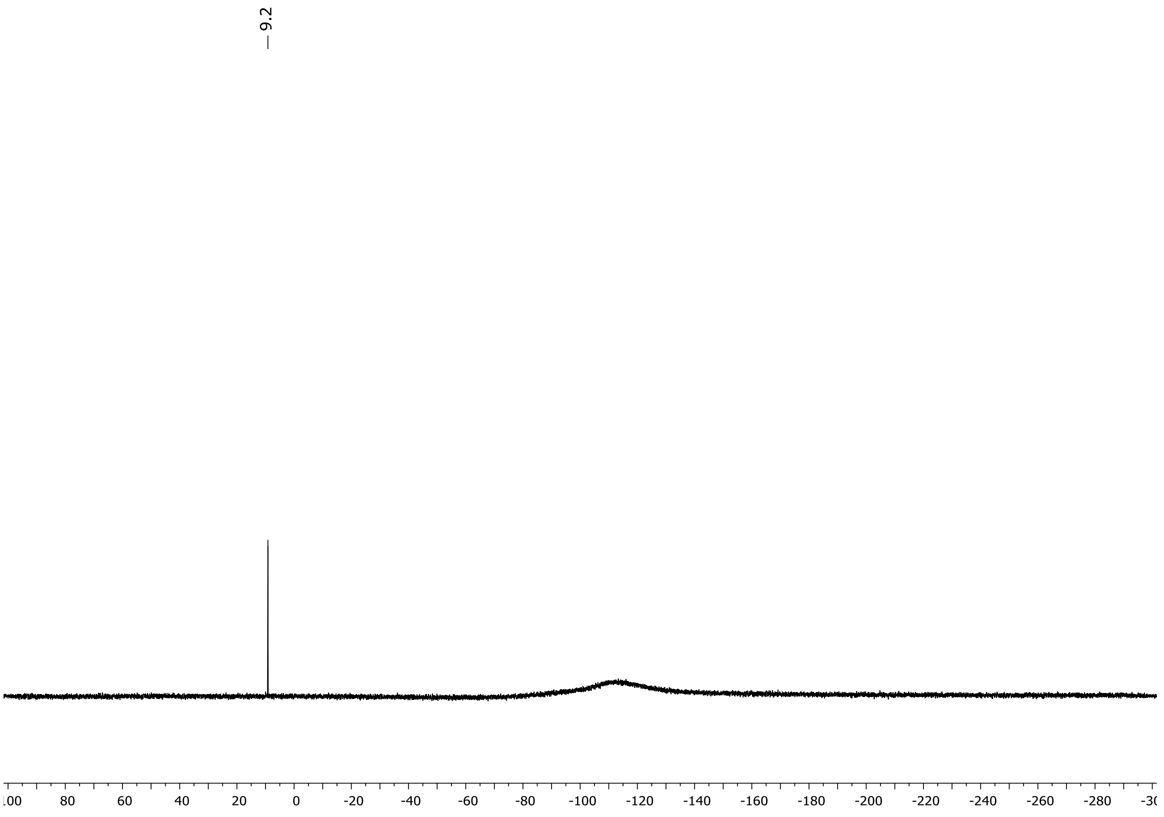

Figure S3. ^{29}Si NMR Spectra of tert-butyldimethyl((triethylgermyl)oxy)silane (**3a**)

Ethyldiisopropyl((triethylgermyl)oxy)silane (3b)

$Et(i\text{-}Pr)_2SiO-GeEt_3$

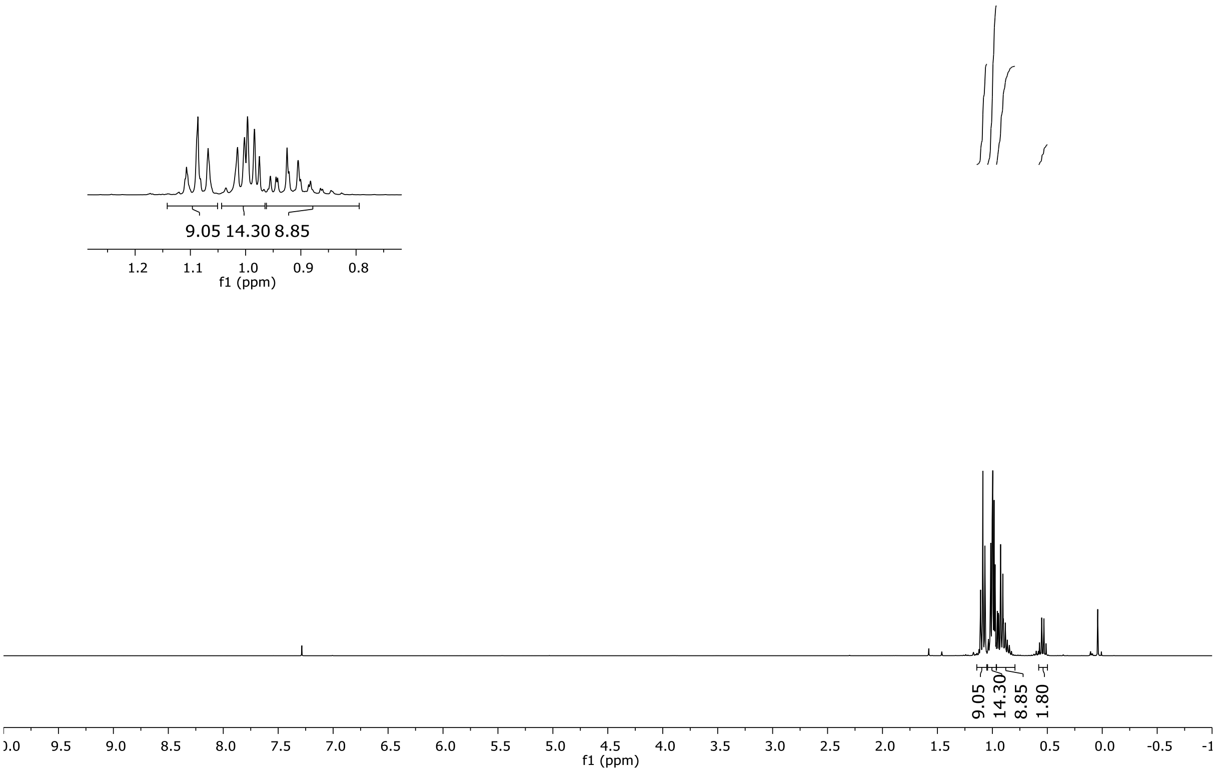

Figure S4. ^{1}H NMR Spectra of ethyldiisopropyl((triethylgermyl)oxy)silane (**3b**)

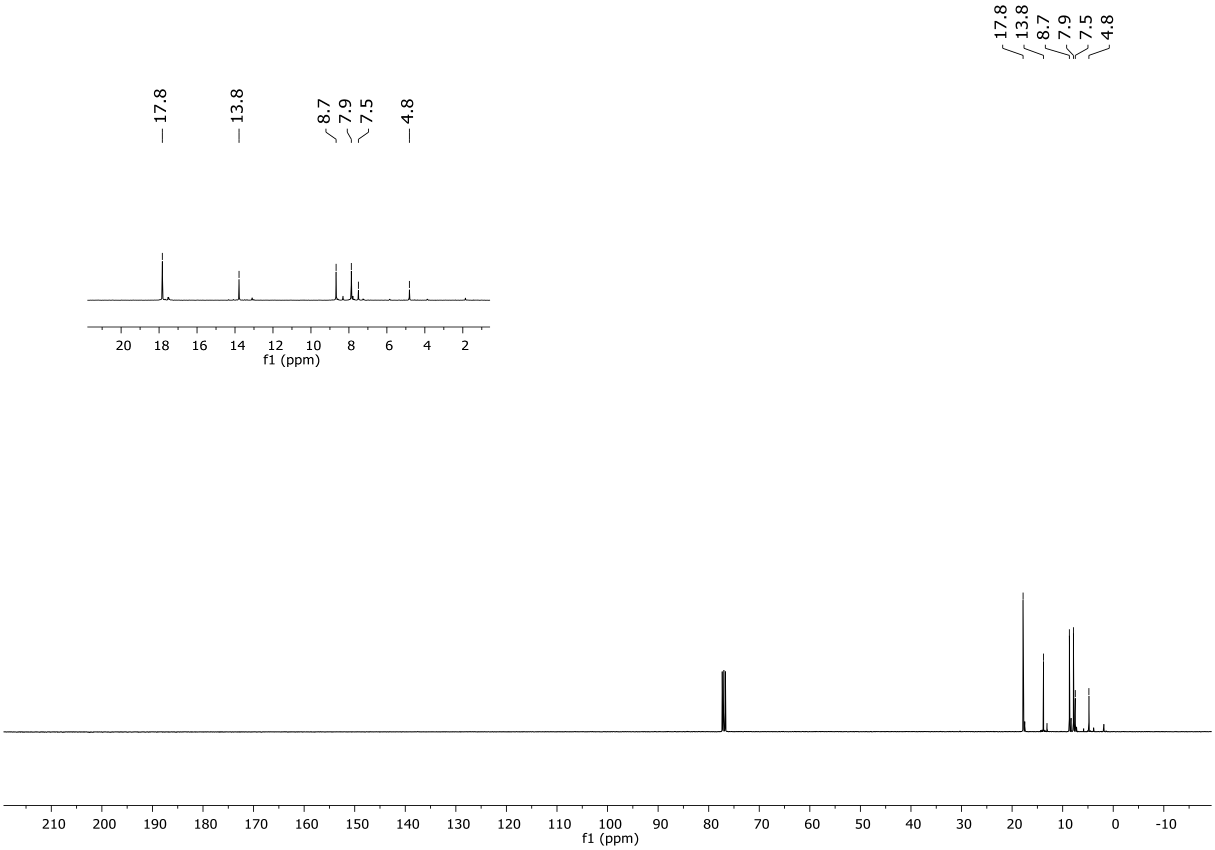

Figure S5. ^{13}C NMR Spectra of ethyldiisopropyl((triethylgermyl)oxy)silane (**3b**)

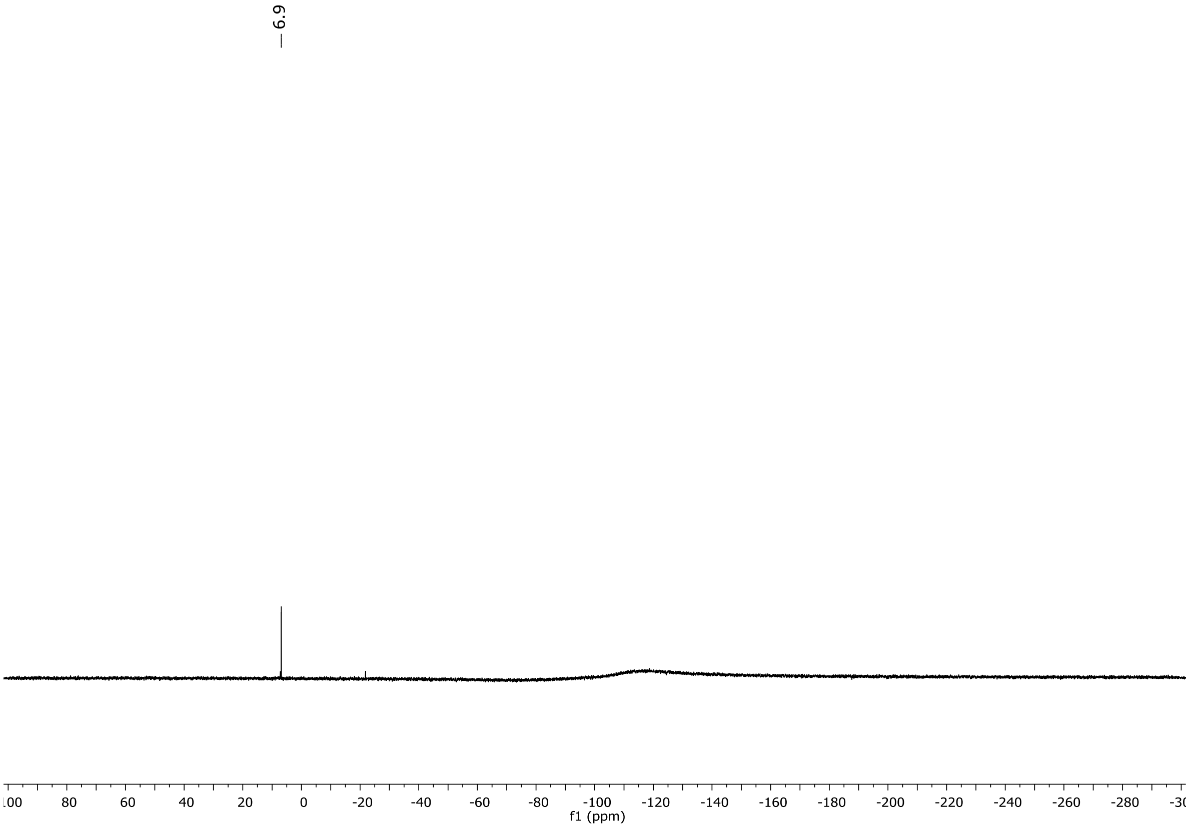

Figure S6. ^{29}Si NMR Spectra of ethyldiisopropyl((triethylgermyl)oxy)silane (**3b**)

Triisopropyl((triethylgermyl)oxy)silane (3c)

$(i\text{-}Pr)_3SiO-GeEt_3$

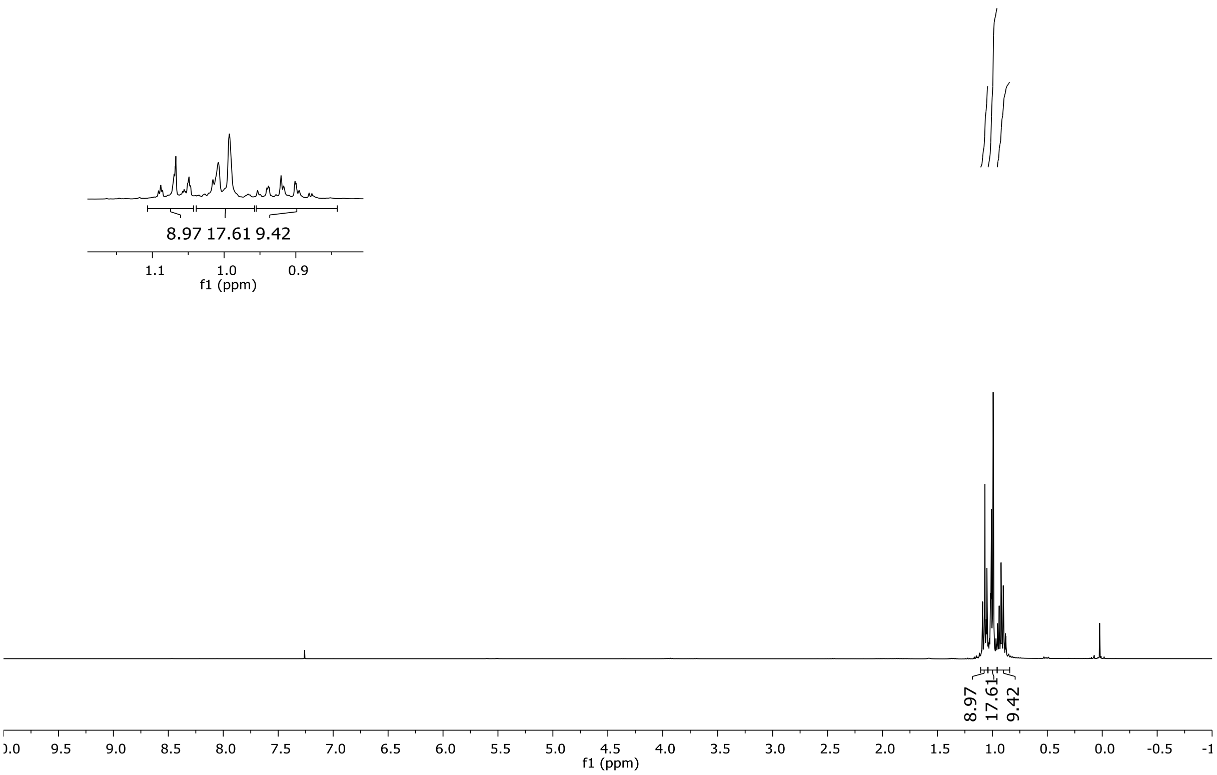

Figure S7. ^{1}H NMR Spectra of triisopropyl((triethylgermyl)oxy)silane (**3c**)

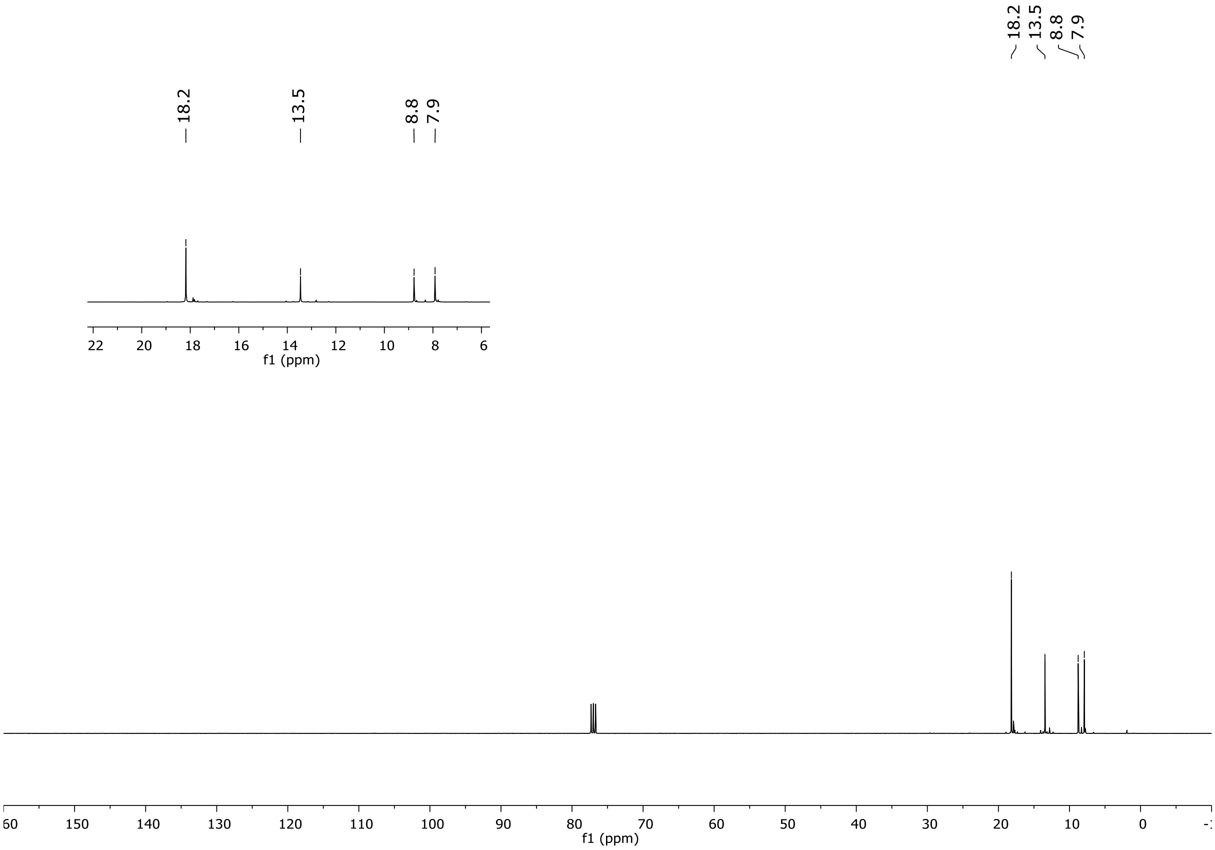

Figure S8. ^{13}C NMR Spectra of triisopropyl((triethylgermyl)oxy)silane (**3c**)

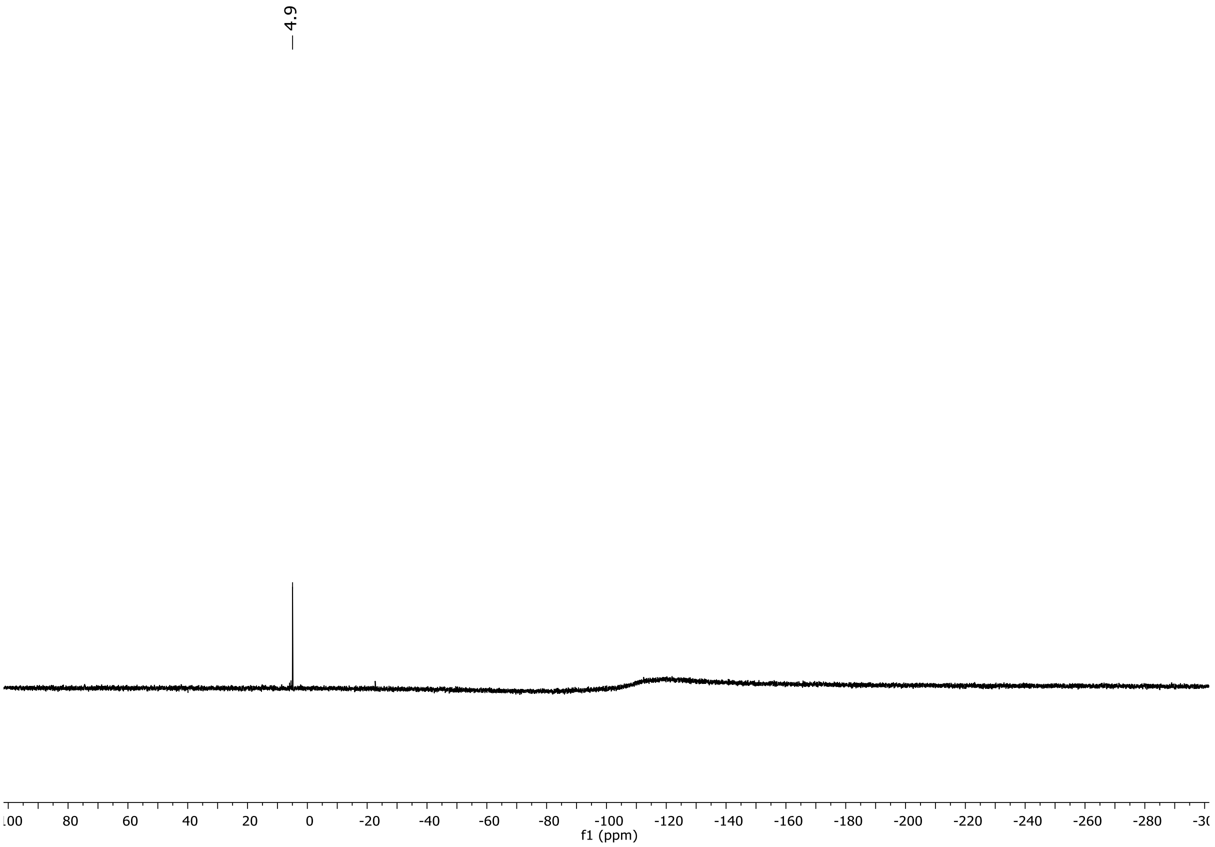

Figure S9. ^{29}Si NMR Spectra of triisopropyl((triethylgermyl)oxy)silane (**3c**)

Triisobutyl((triethylgermyl)oxy)silane (3d)

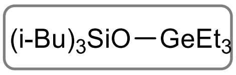

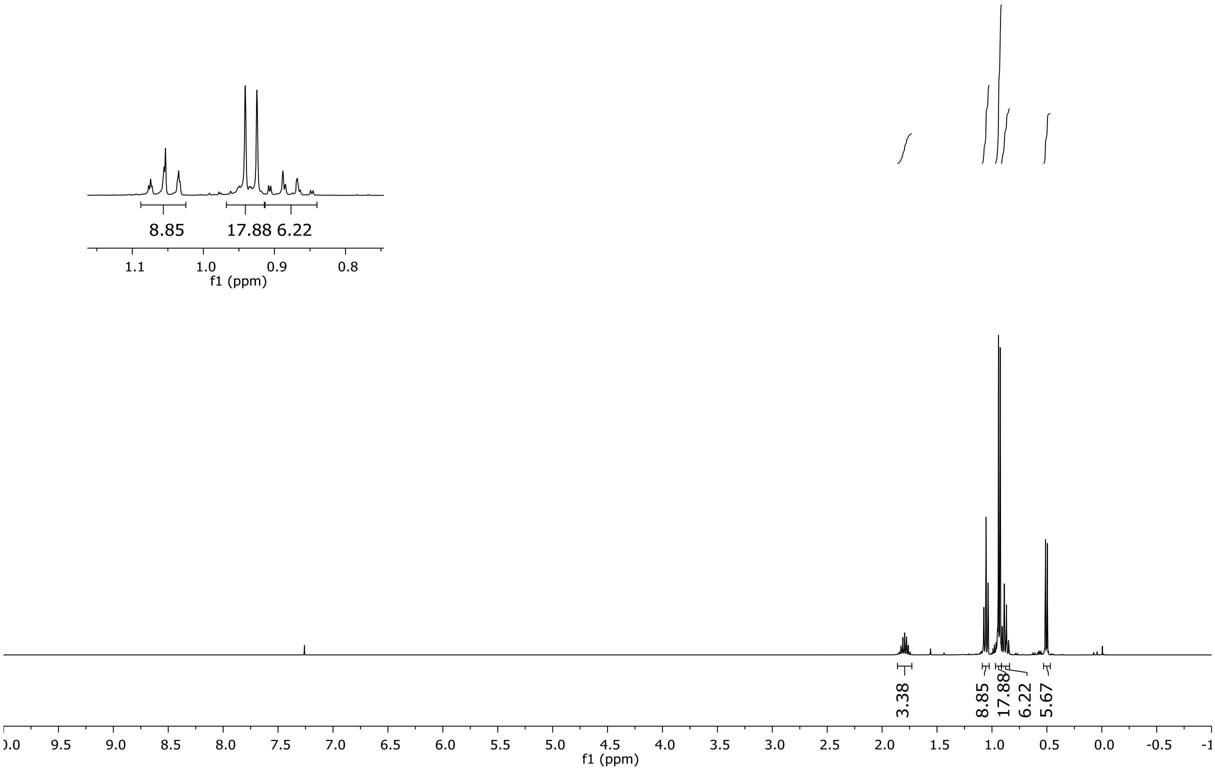

Figure S10. ^{1}H NMR Spectra of triisobutyl((triethylgermyl)oxy)silane (**3d**)

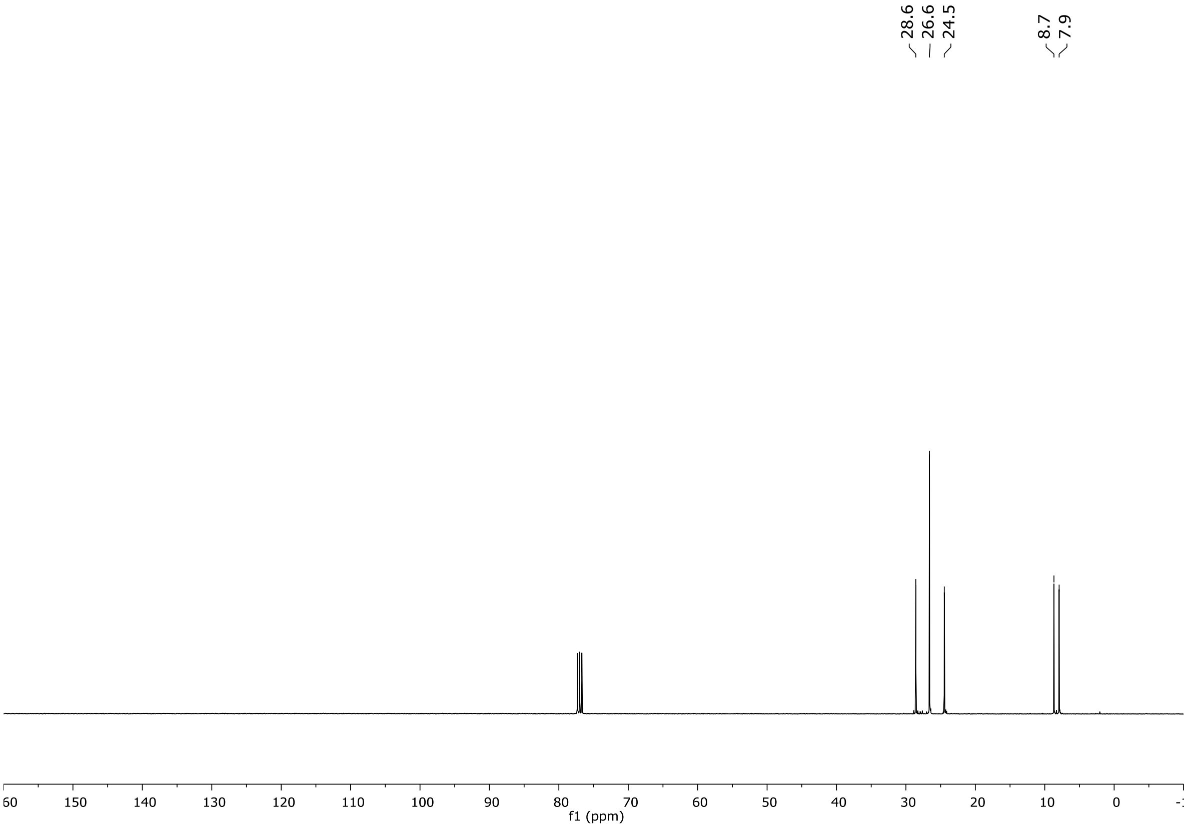

Figure S11. ^{13}C NMR Spectra of triisobutyl((triethylgermyl)oxy)silane (**3d**)

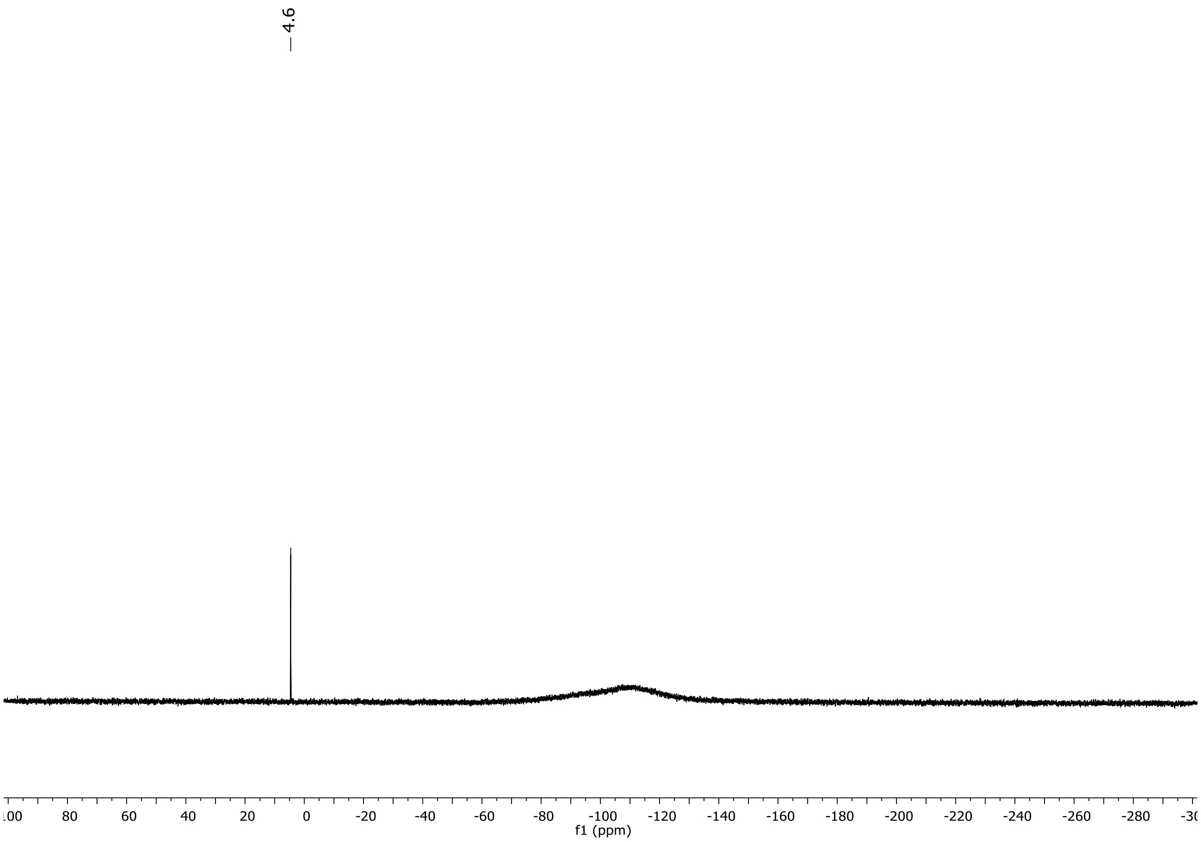

Figure S12. ^{29}Si NMR Spectra of triisobutyl((triethylgermyl)oxy)silane (**3d**)

Tributyl((triethylgermyl)oxy)silane (3e)

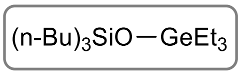

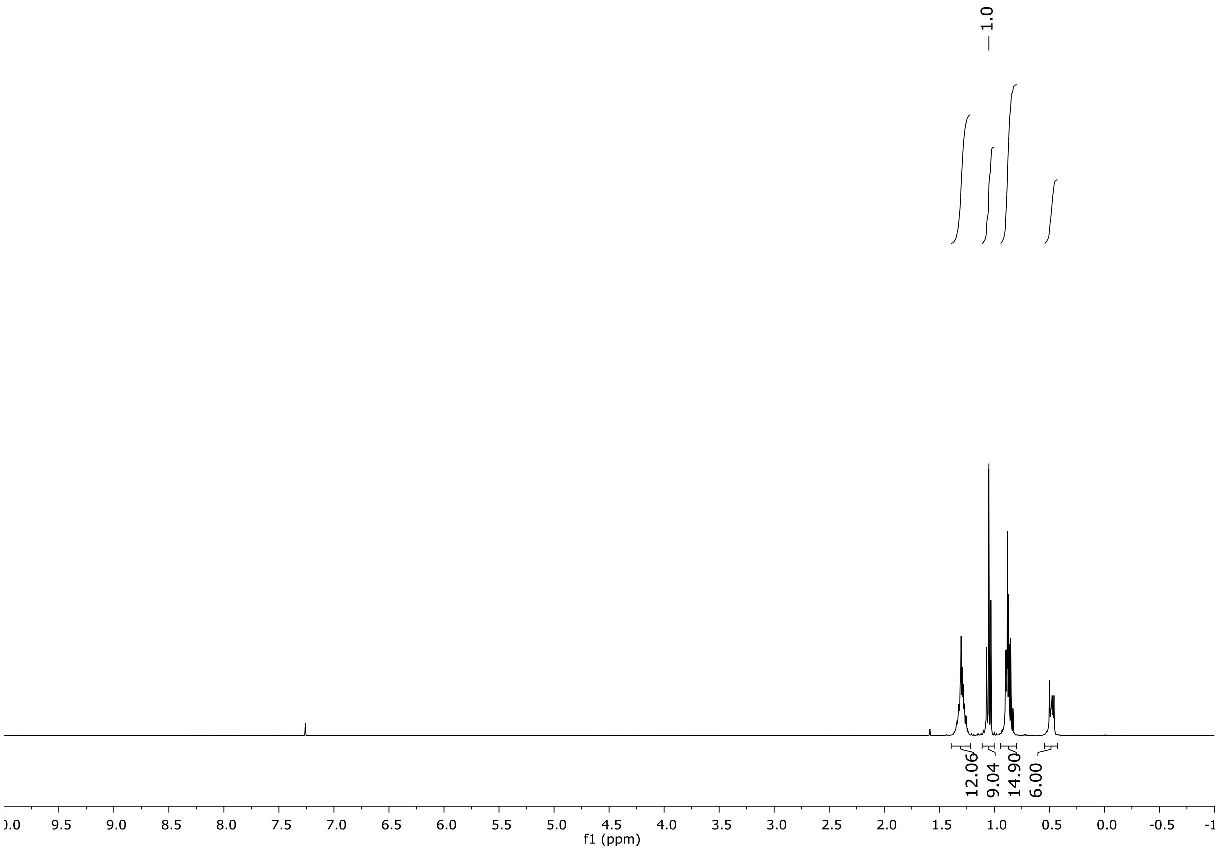

Figure S13. ^{1}H NMR Spectra of tributyl((triethylgermyl)oxy)silane (**3e**)

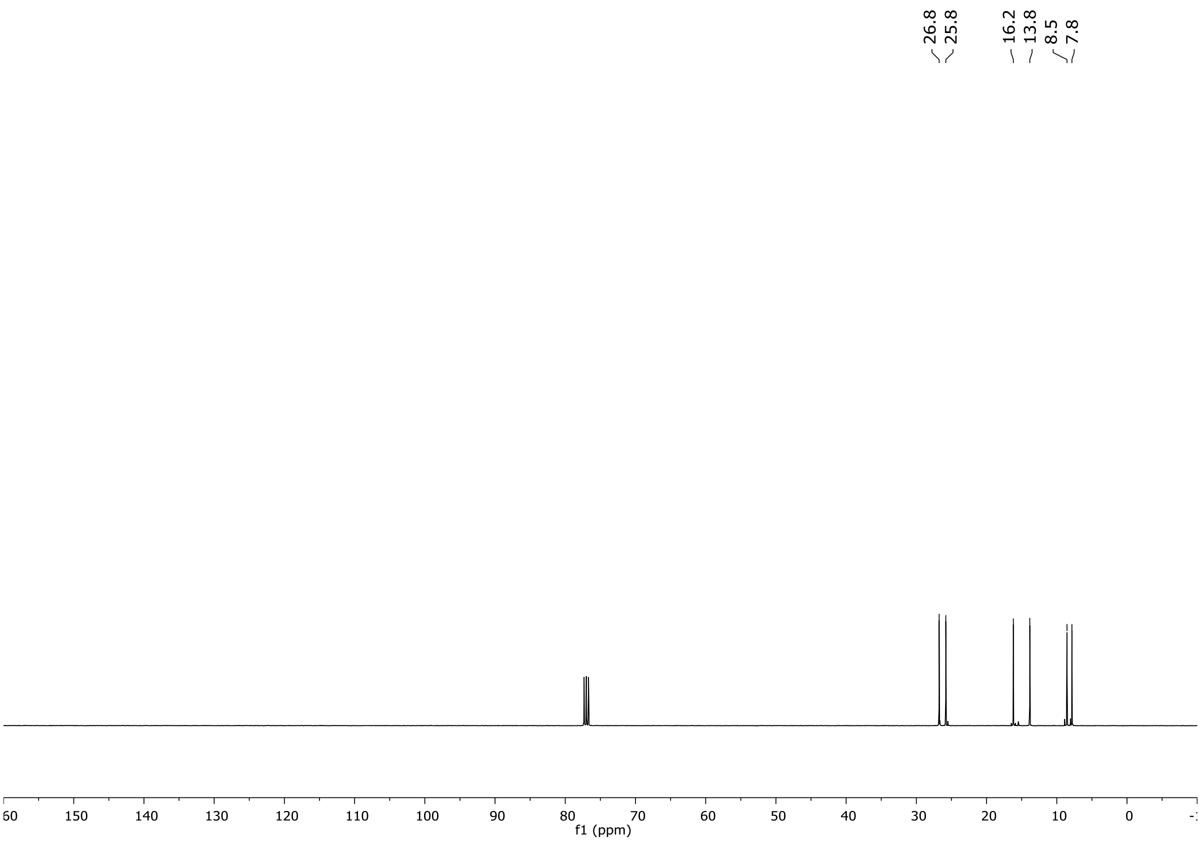

Figure S14. ^{13}C NMR Spectra of tributyl((triethylgermyl)oxy)silane (**3e**)

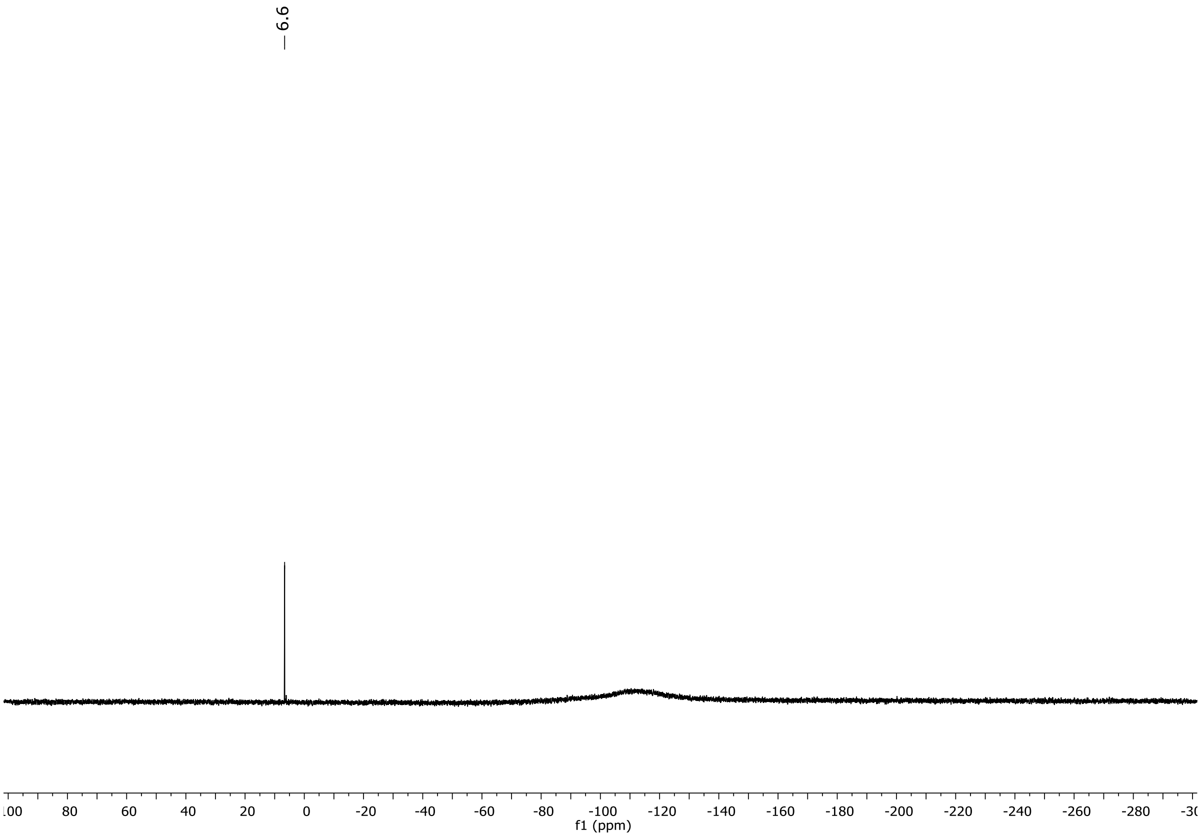

Figure S15. ^{29}Si NMR Spectra of tributyl((triethylgermyl)oxy)silane (**3e**)

Tri-tert-butyl(triethylgermyl)orthosilicate (3f)

$(t\text{-}BuO)_3SiO{-}GeEt_3$

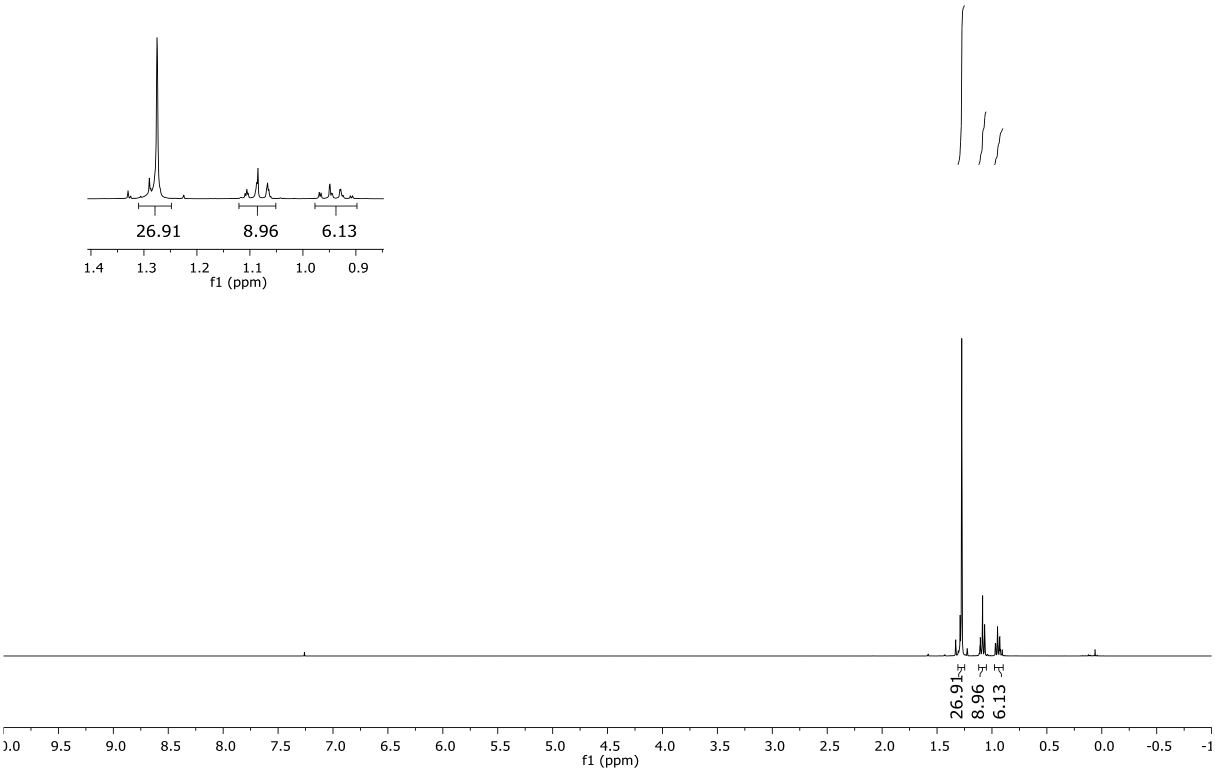

Figure S16. ^{1}H NMR Spectra of tri-tert-butyl(triethylgermyl)orthosilicate (**3f**)

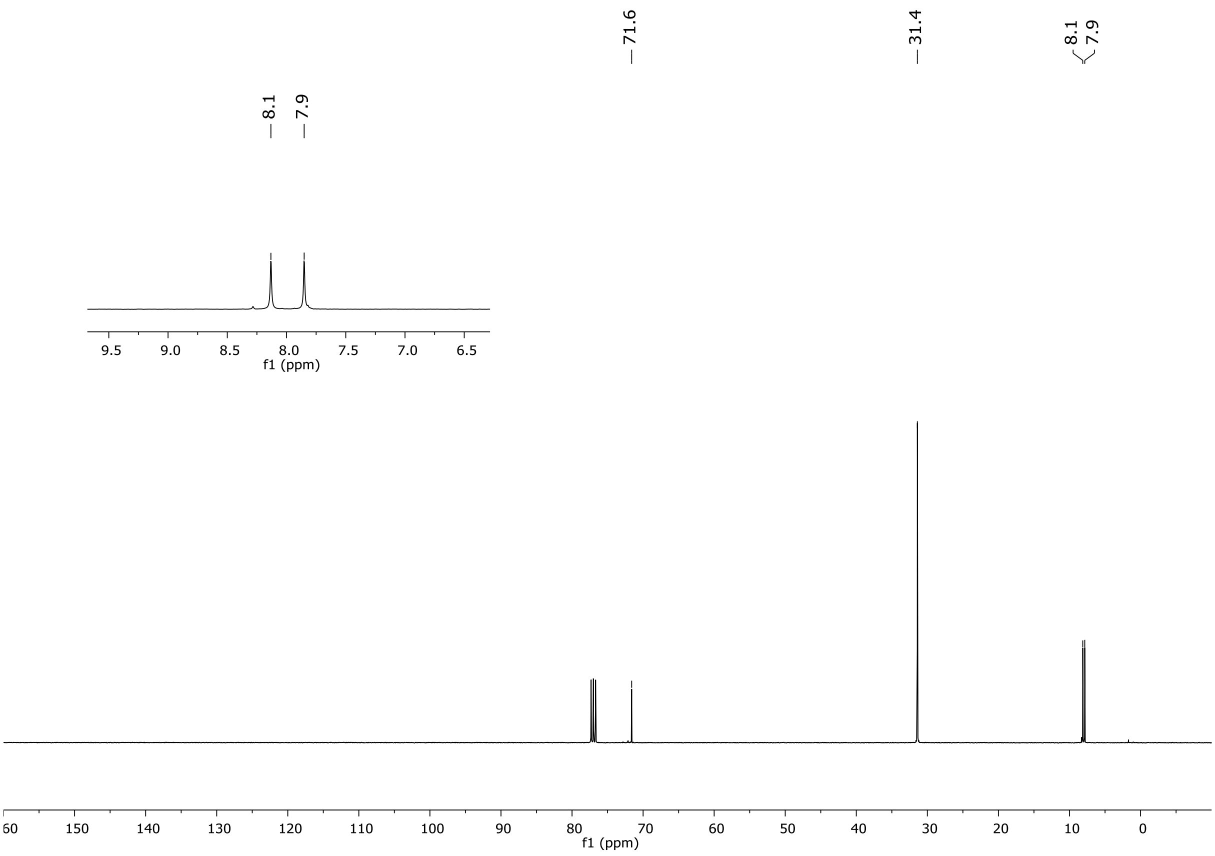

Figure S17. ^{13}C NMR Spectra of tri-tert-butyl(triethylgermyl)orthosilicate (**3f**)

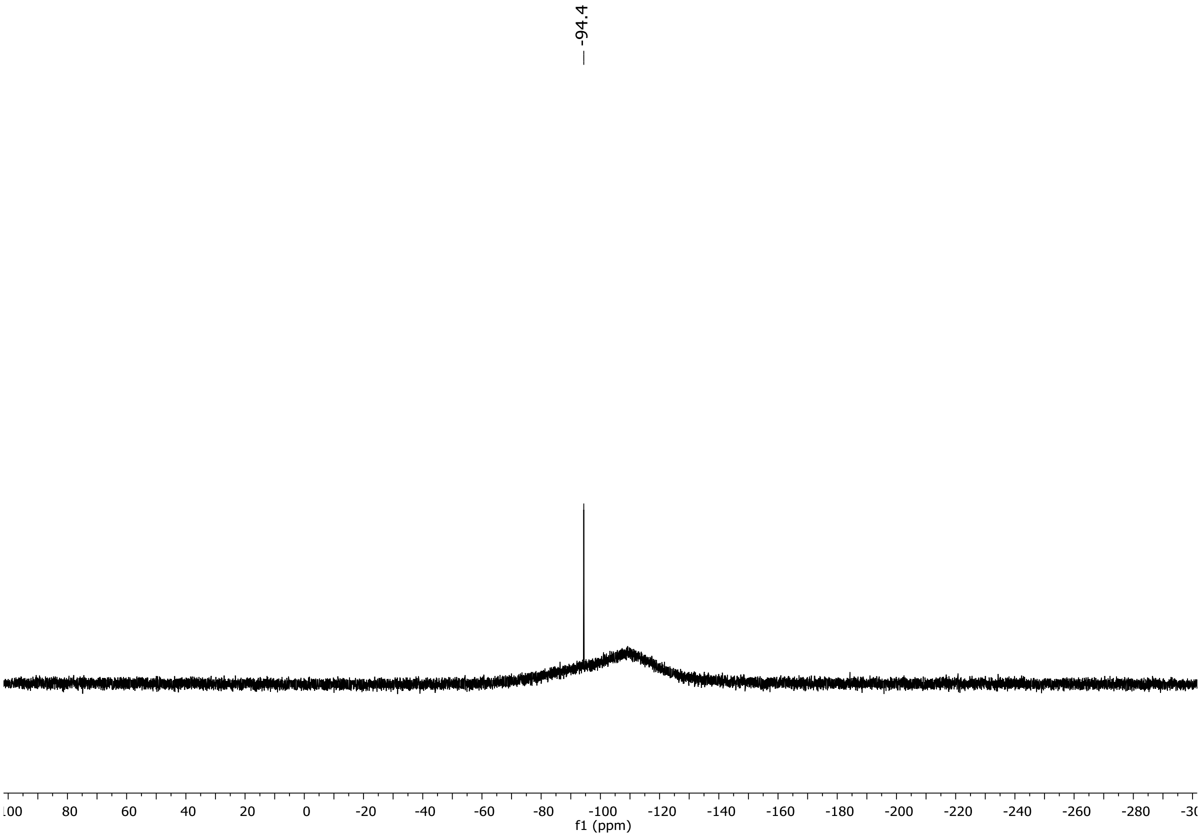

Figure S18. ^{29}Si NMR Spectra of tri-tert-butyl(triethylgermyl)orthosilicate (**3f**)

Tert-butyldiphenyl((triethylgermyl)oxy)silane (3g)

t-$BuPh_2SiO$—$GeEt_3$

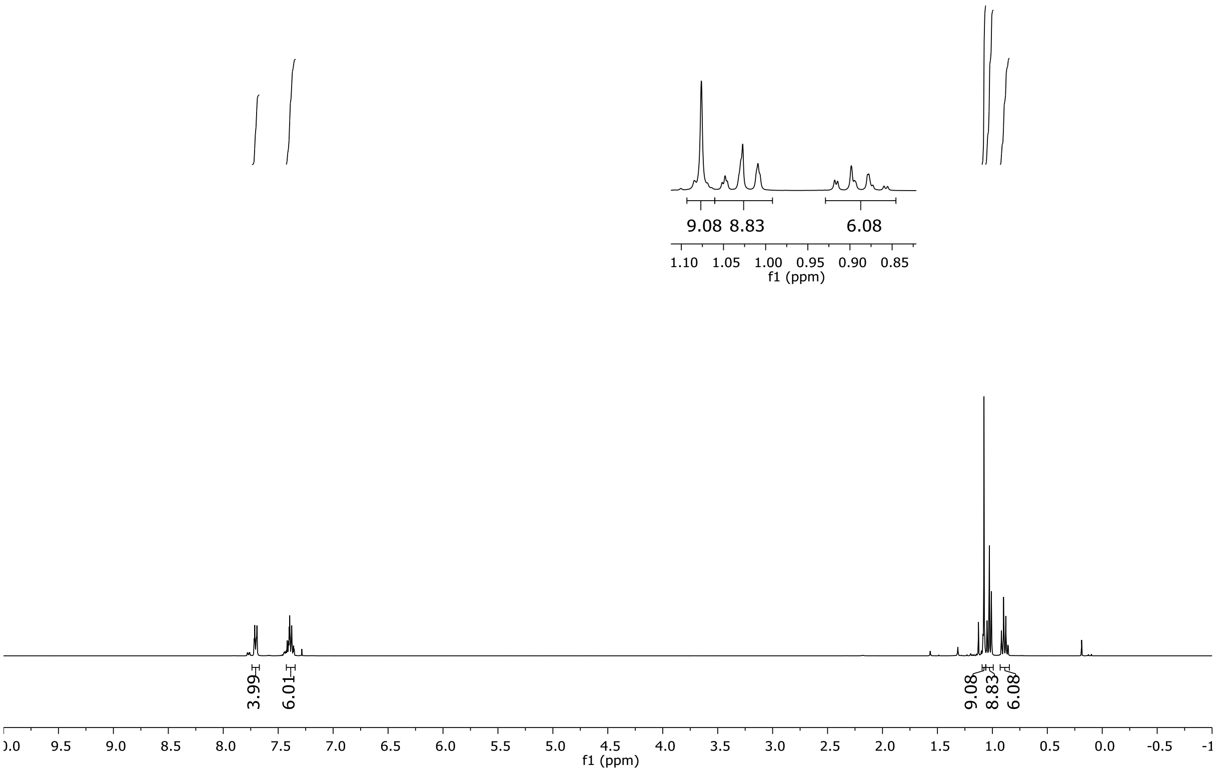

Figure S19. ^{1}H NMR Spectra of tert-butyldiphenyl((triethylgermyl)oxy)silane (**3g**)

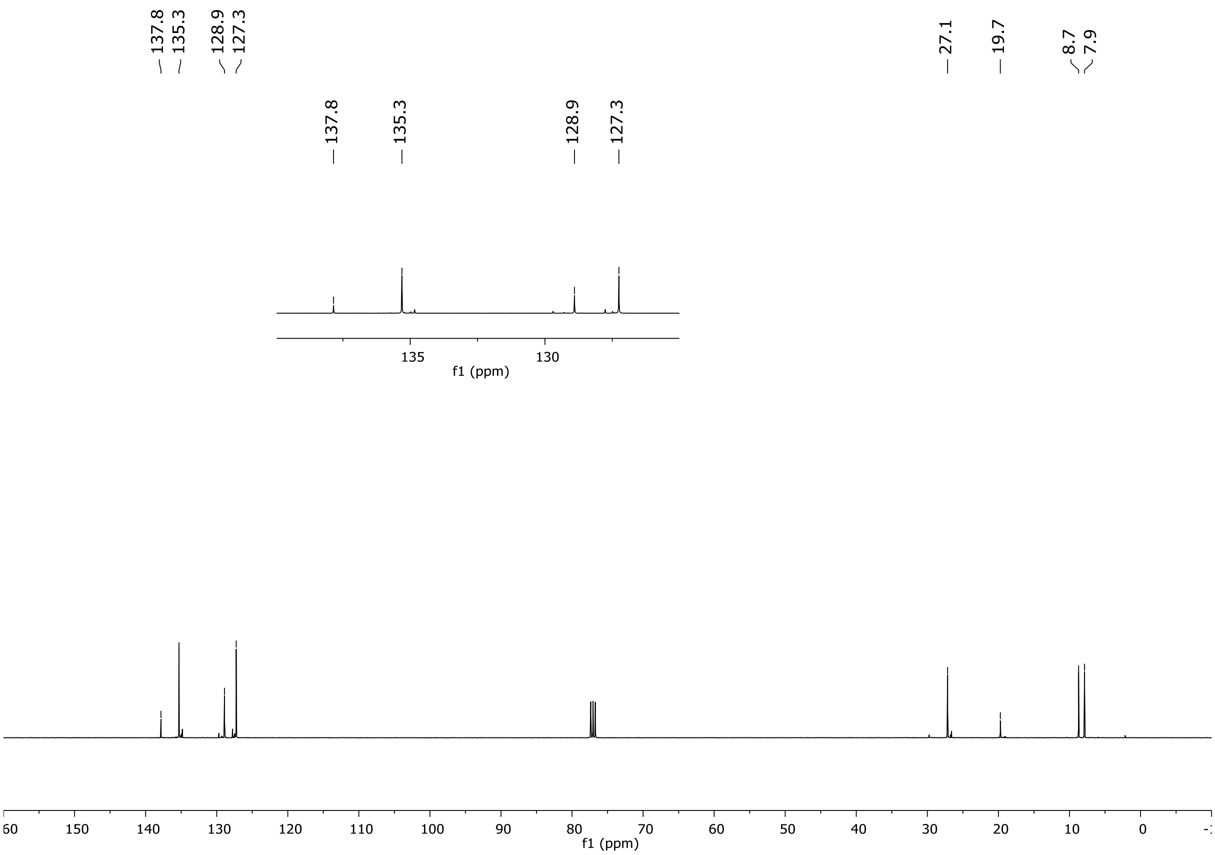

Figure S20. ^{13}C NMR Spectra of tert-butyldiphenyl((triethylgermyl)oxy)silane (**3g**)

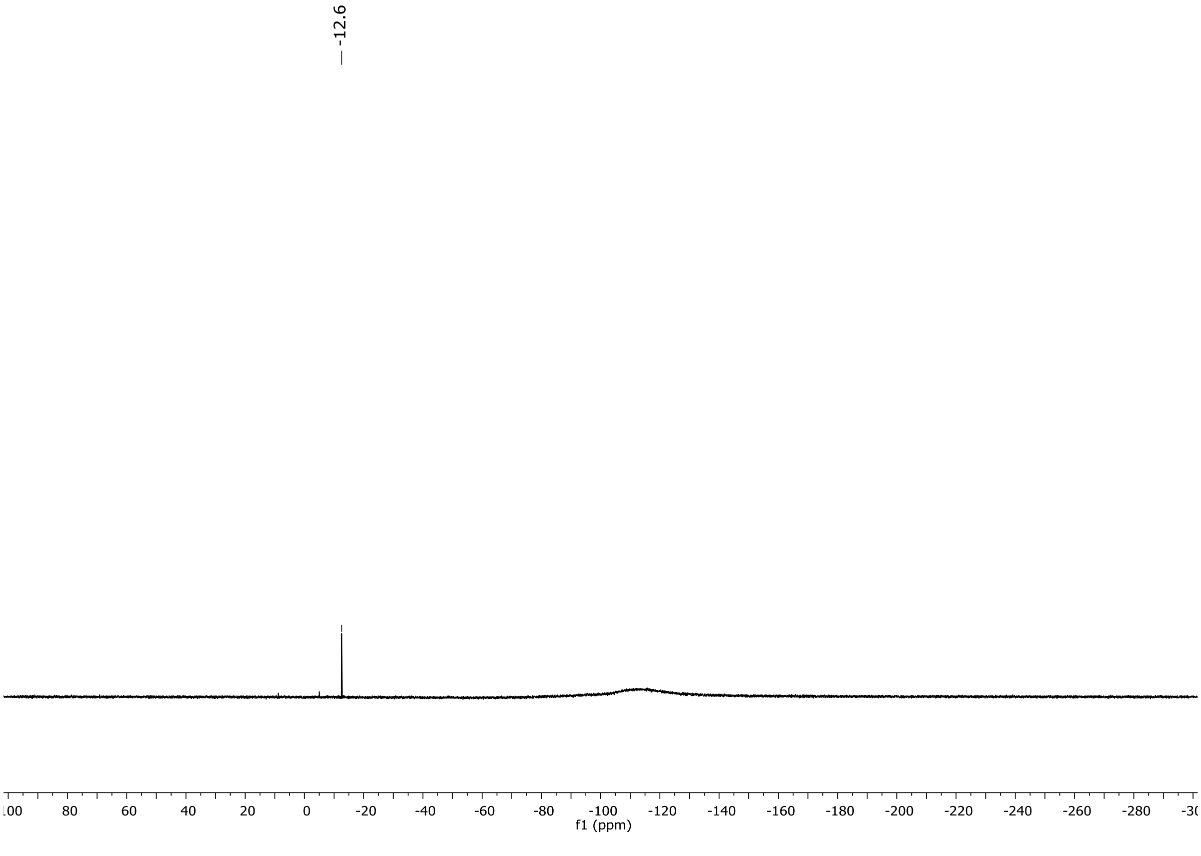

Figure S21. ^{29}Si NMR Spectra of tert-butyldiphenyl((triethylgermyl)oxy)silane (**3g**)

Triphenyl((triethylgermyl)oxy)silane (3h)

$Ph_3SiO—GeEt_3$

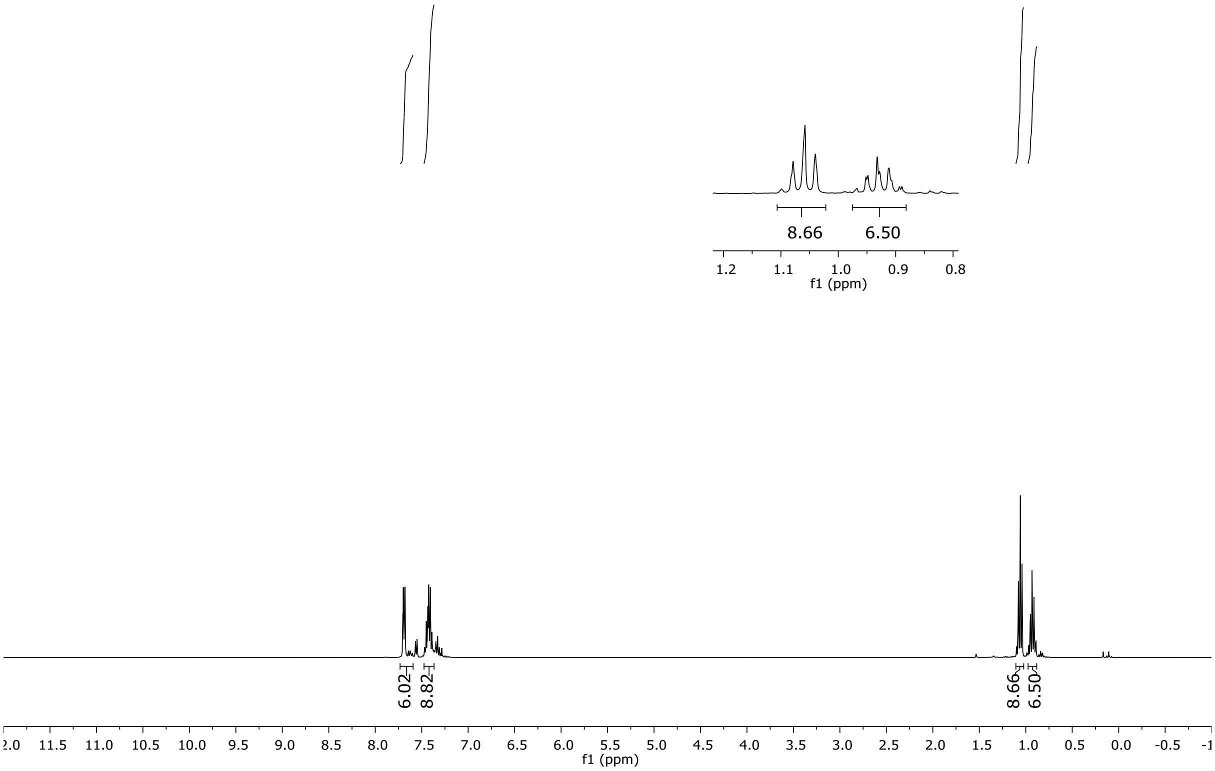

Figure S22. ^{1}H NMR Spectra of triphenyl((triethylgermyl)oxy)silane (**3h**)

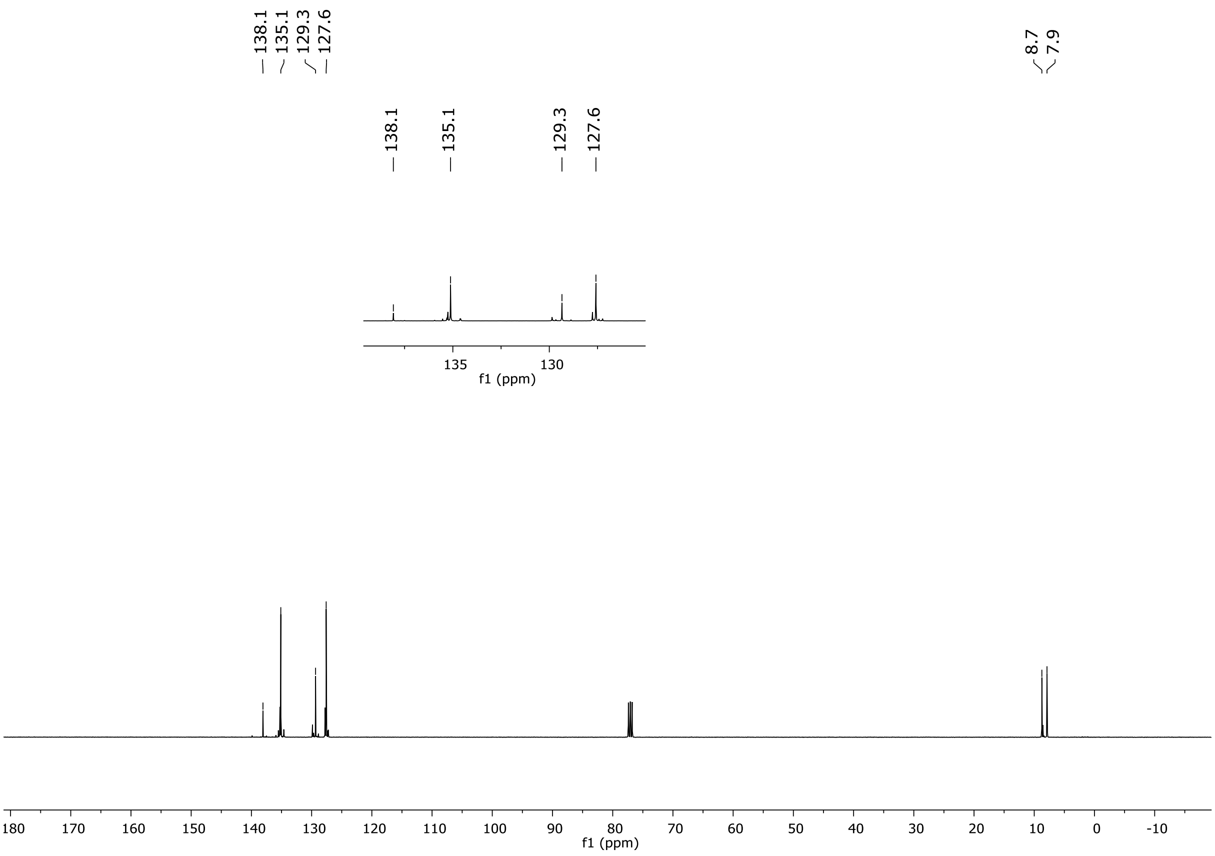

Figure S23. ^{13}C NMR Spectra of triphenyl((triethylgermyl)oxy)silane (**3h**)

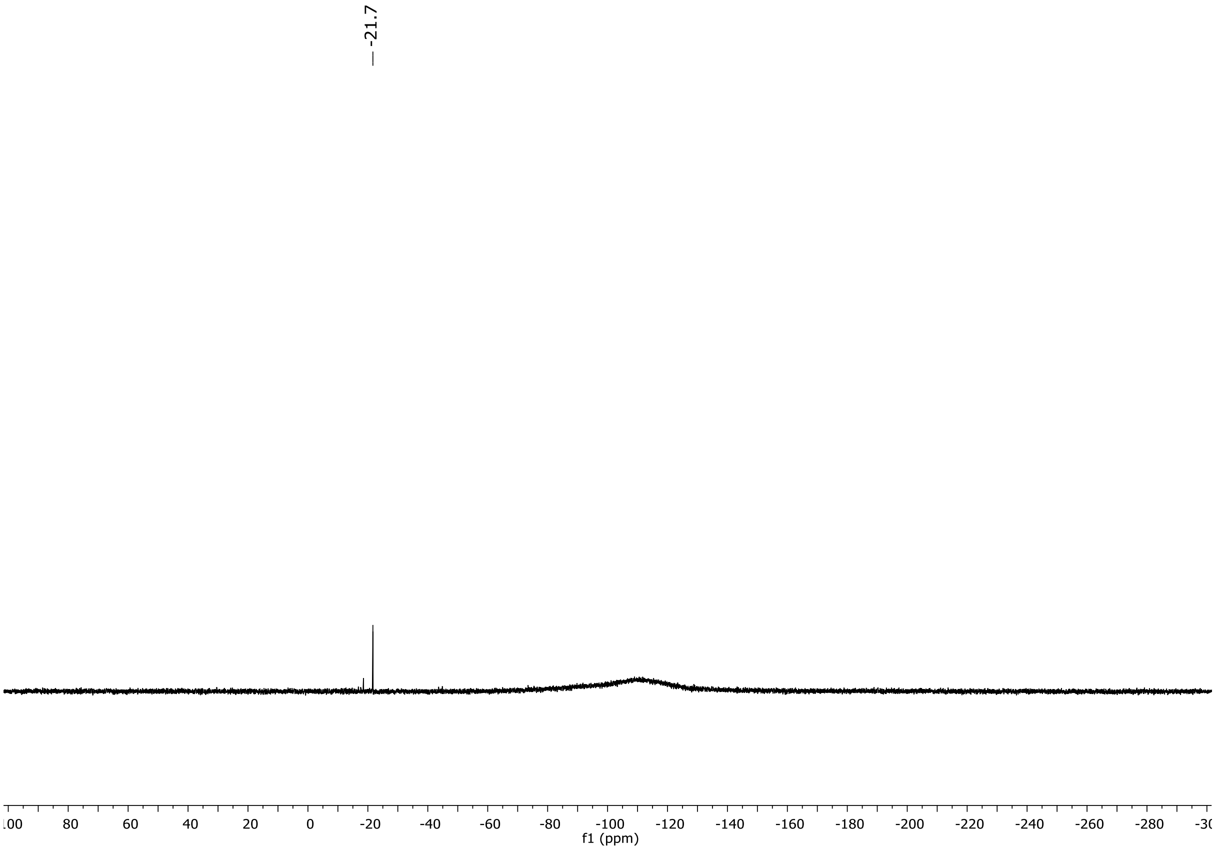

Figure S24. ^{29}Si NMR Spectra of triphenyl((triethylgermyl)oxy)silane (**3h**)

Triethyl((tributylgermyl)oxy)silane (3i)

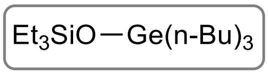

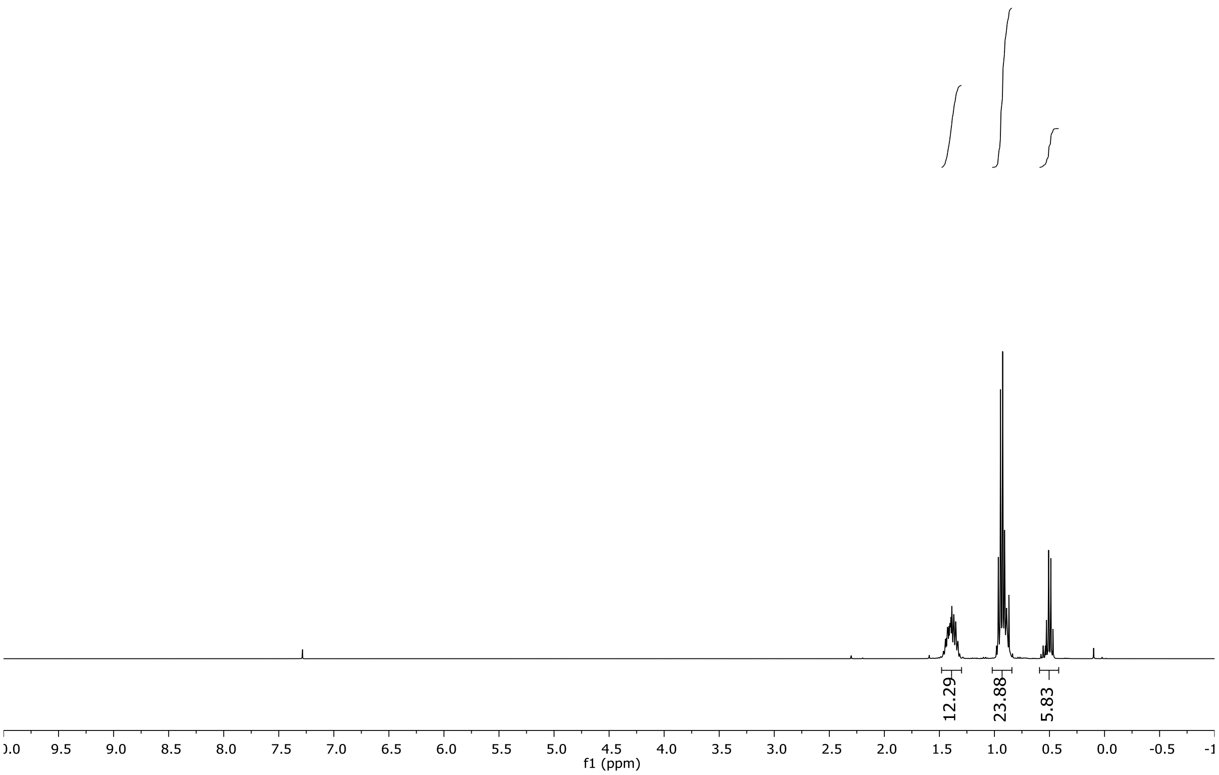

Figure S25. ^{1}H NMR Spectra of triethyl((tributylgermyl)oxy)silane (**3i**)

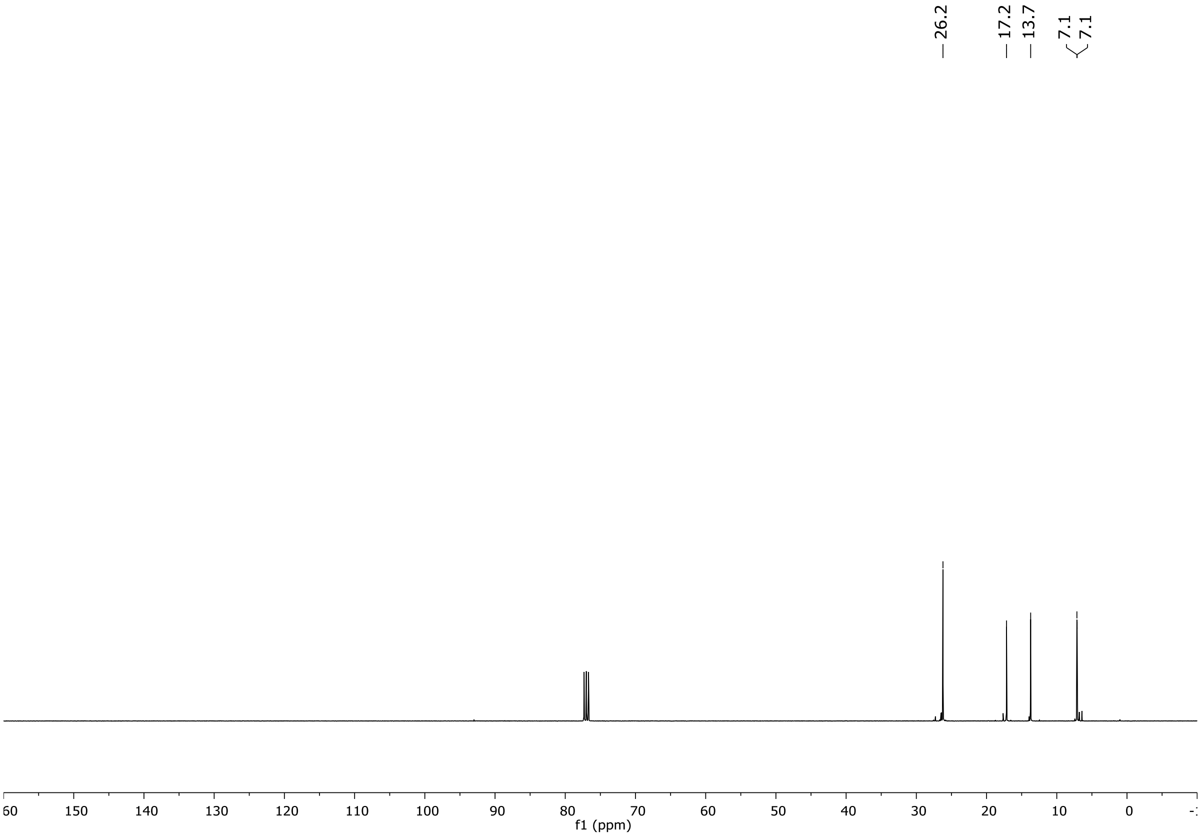

Figure S26. ^{13}C NMR Spectra of triethyl((tributylgermyl)oxy)silane (**3i**)

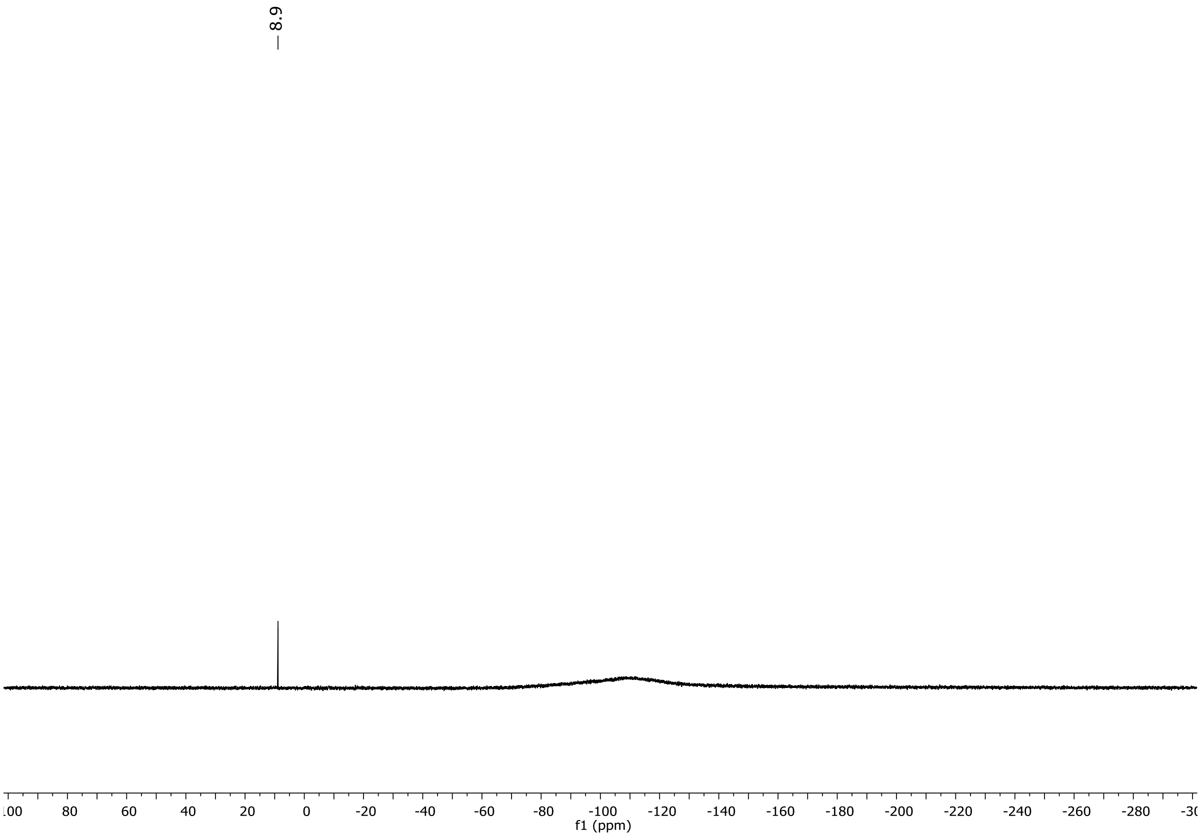

Figure S27. ^{29}Si NMR Spectra of triethyl((tributylgermyl)oxy)silane (**3i**)

Tert-butyldimethyl((tributylgermyl)oxy)silane (3j)

t-BuMe$_2$SiO—Ge(n-Bu)$_3$

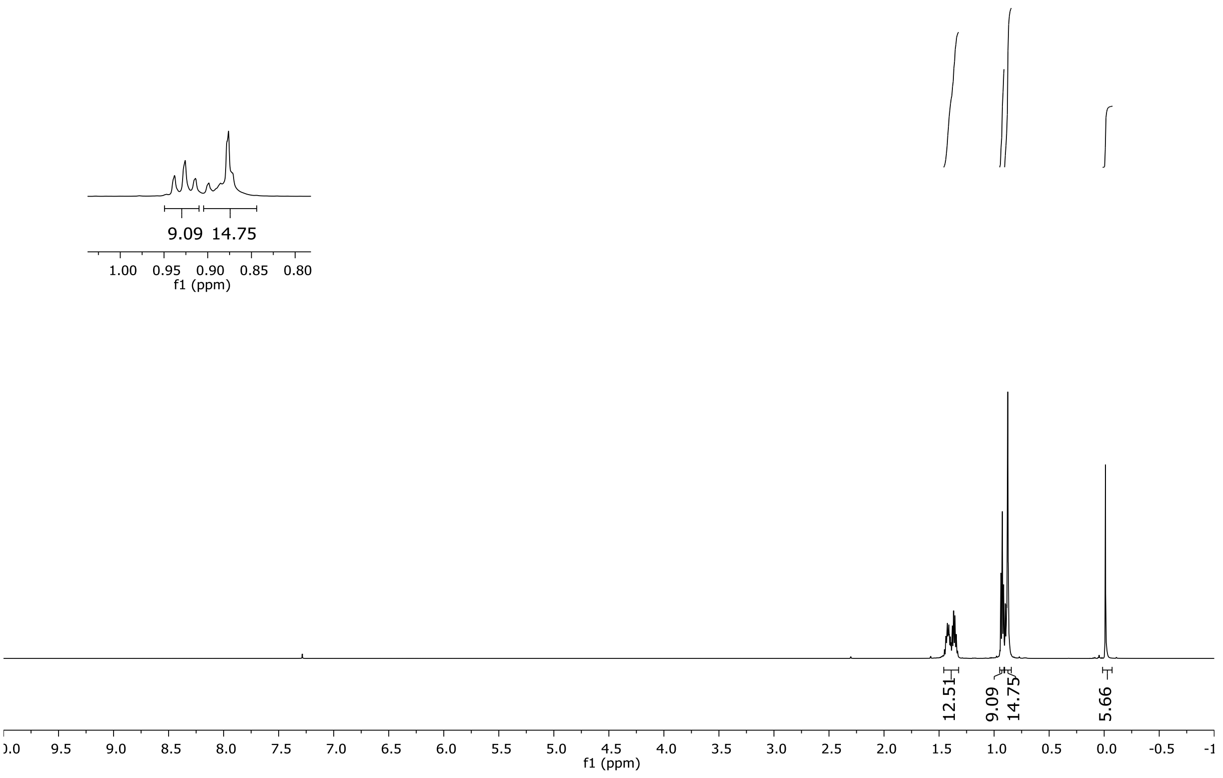

Figure S28. ^{1}H NMR Spectra of tert-butyldimethyl((tributylgermyl)oxy)silane (**3j**)

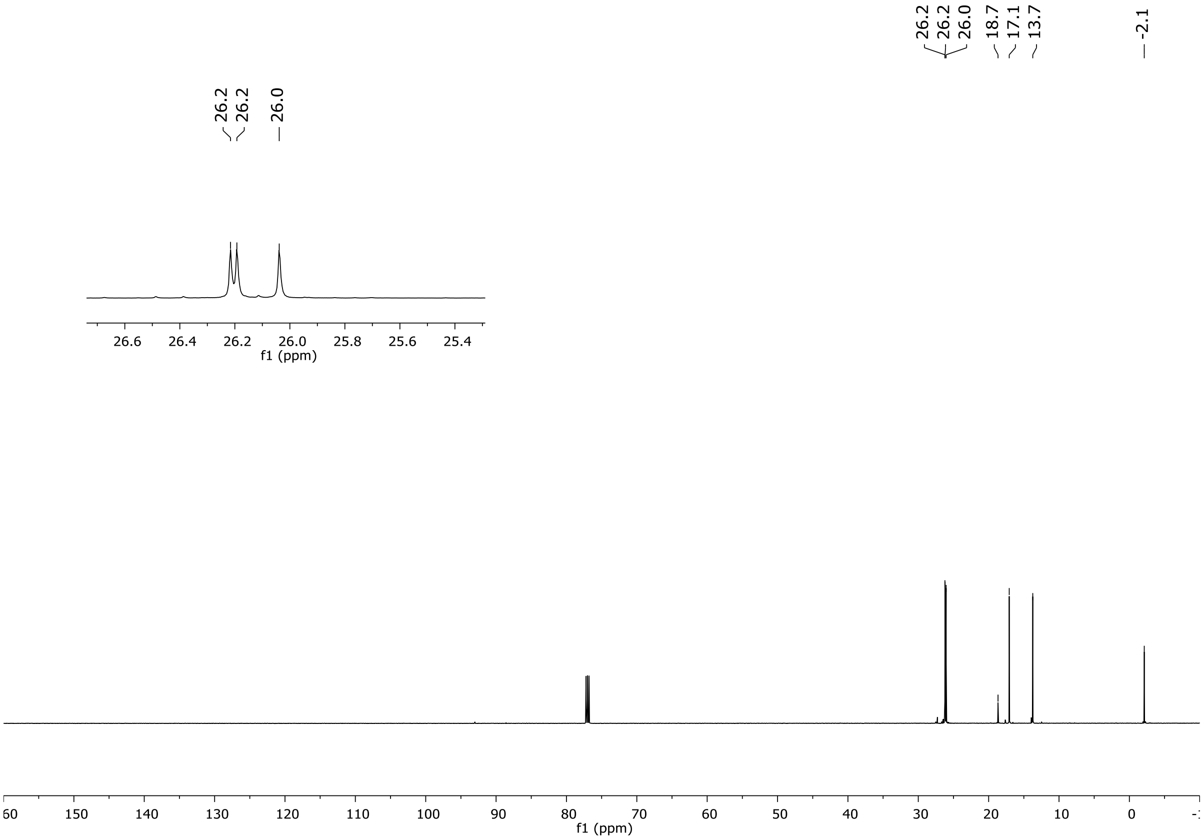

Figure S29. ^{13}C NMR Spectra of tert-butyldimethyl((tributylgermyl)oxy)silane (**3j**)

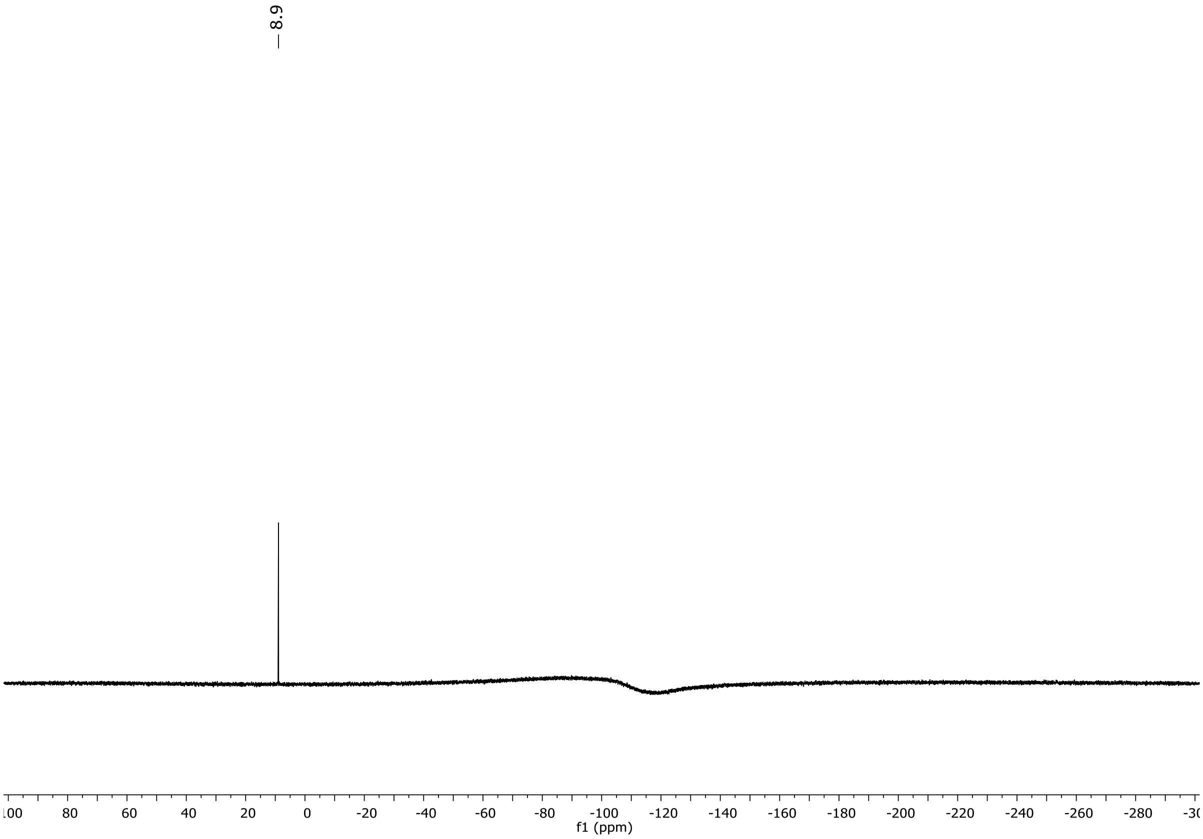

Figure S30. ^{29}Si NMR Spectra of tert-butyldimethyl((tributylgermyl)oxy)silane (**3j**)

Triisopropyl((tributylgermyl)oxy)silane (3k)

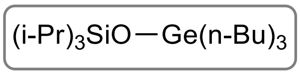

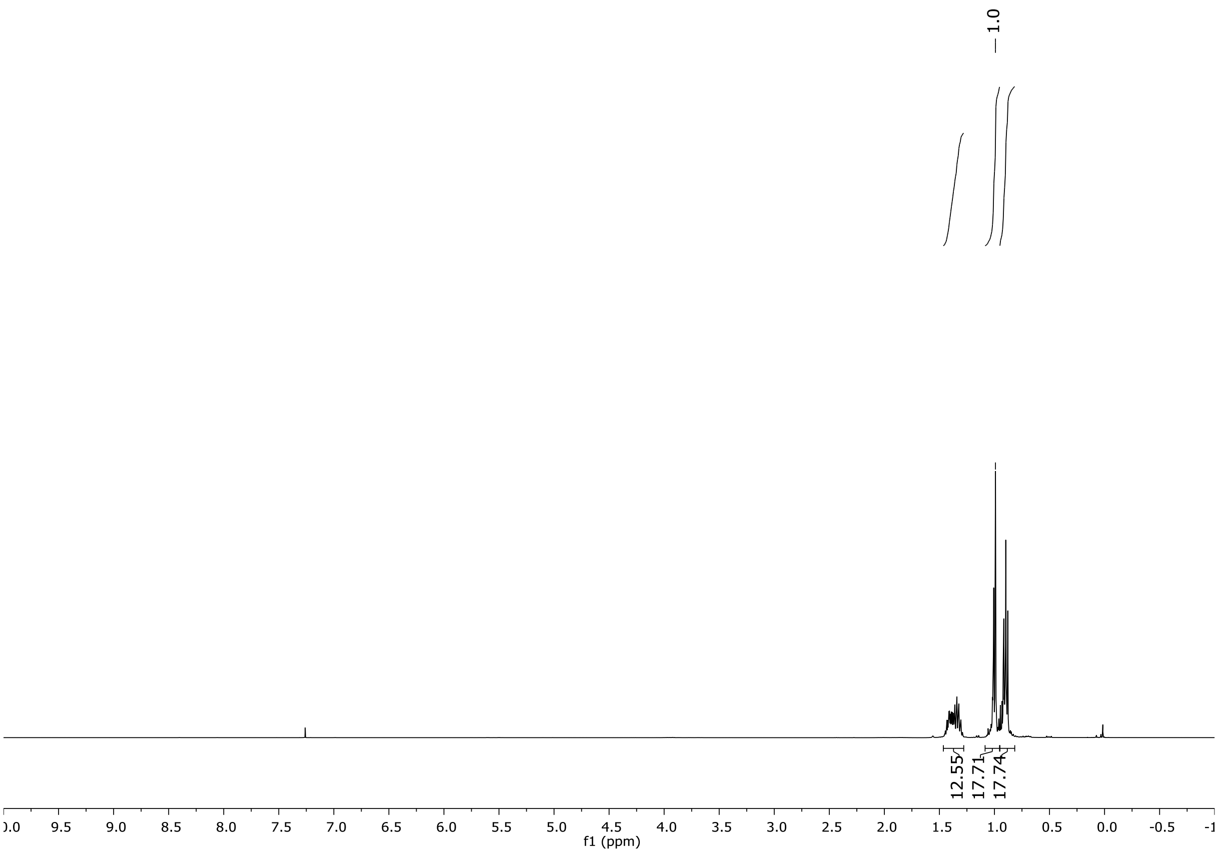

Figure S31. ^{1}H NMR Spectra of triisopropyl((tributylgermyl)oxy)silane (**3k**)

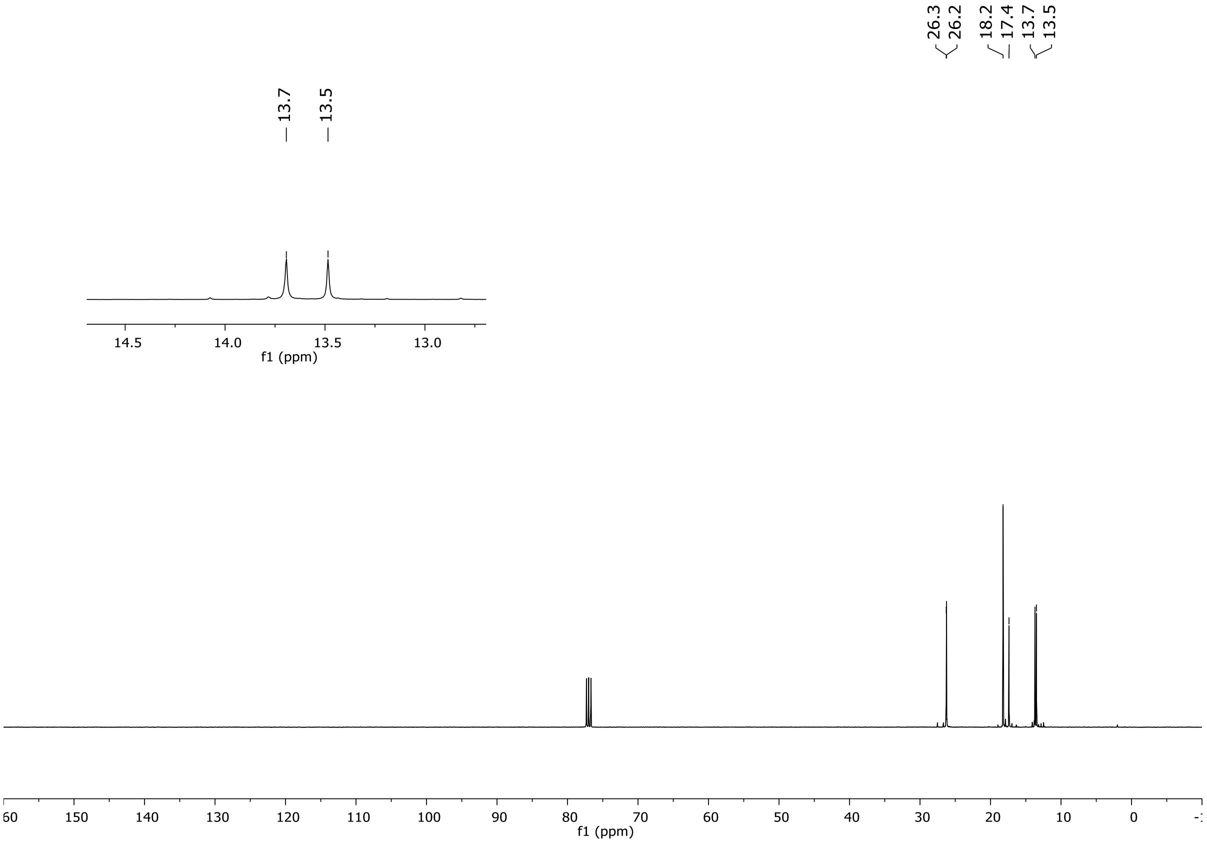

Figure S32. ^{13}C NMR Spectra of triisopropyl((tributylgermyl)oxy)silane (**3k**)

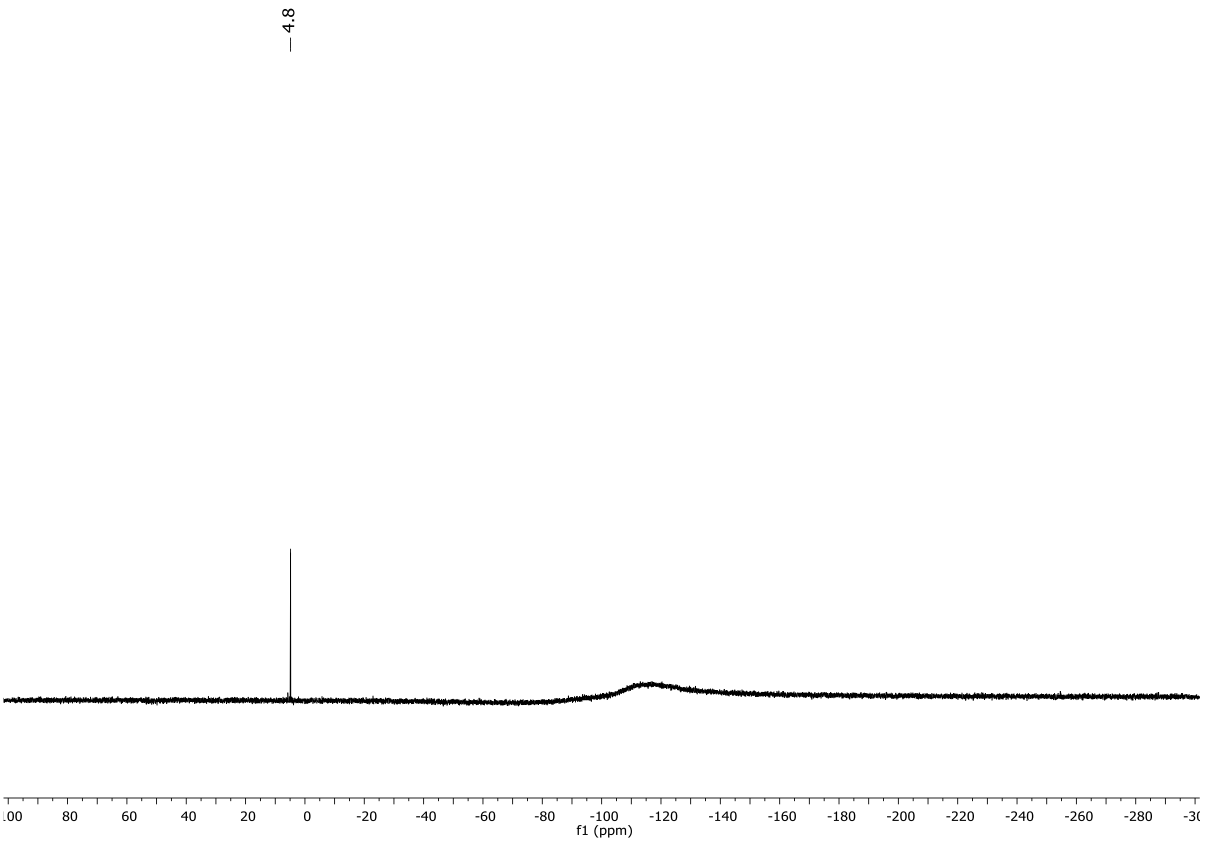

Figure S33. ^{29}Si NMR Spectra of triisopropyl((tributylgermyl)oxy)silane (**3k**)

((Dimethyl(phenyl)germyl)oxy)triethylsilane (3l)

$Et_3SiO-GeMe_2Ph$

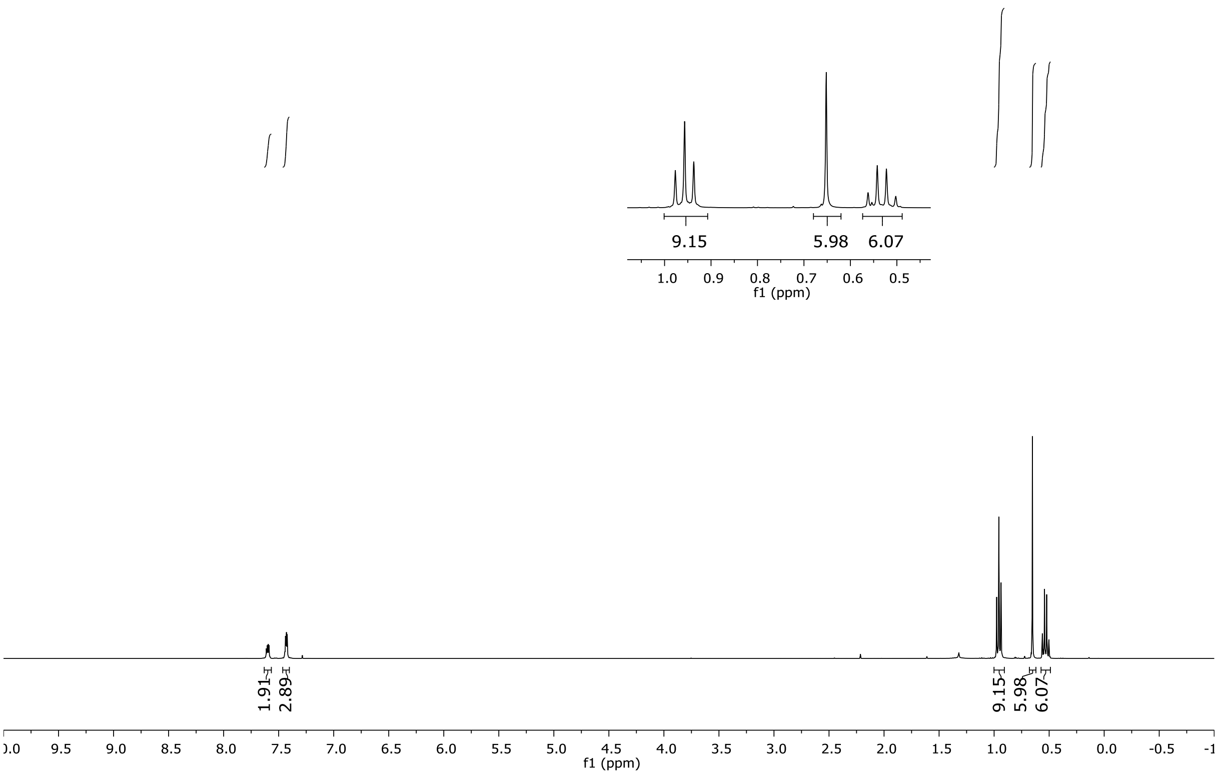

Figure S34. ^{1}H NMR Spectra of ((dimethyl(phenyl)germyl)oxy)triethylsilane (**3l**)

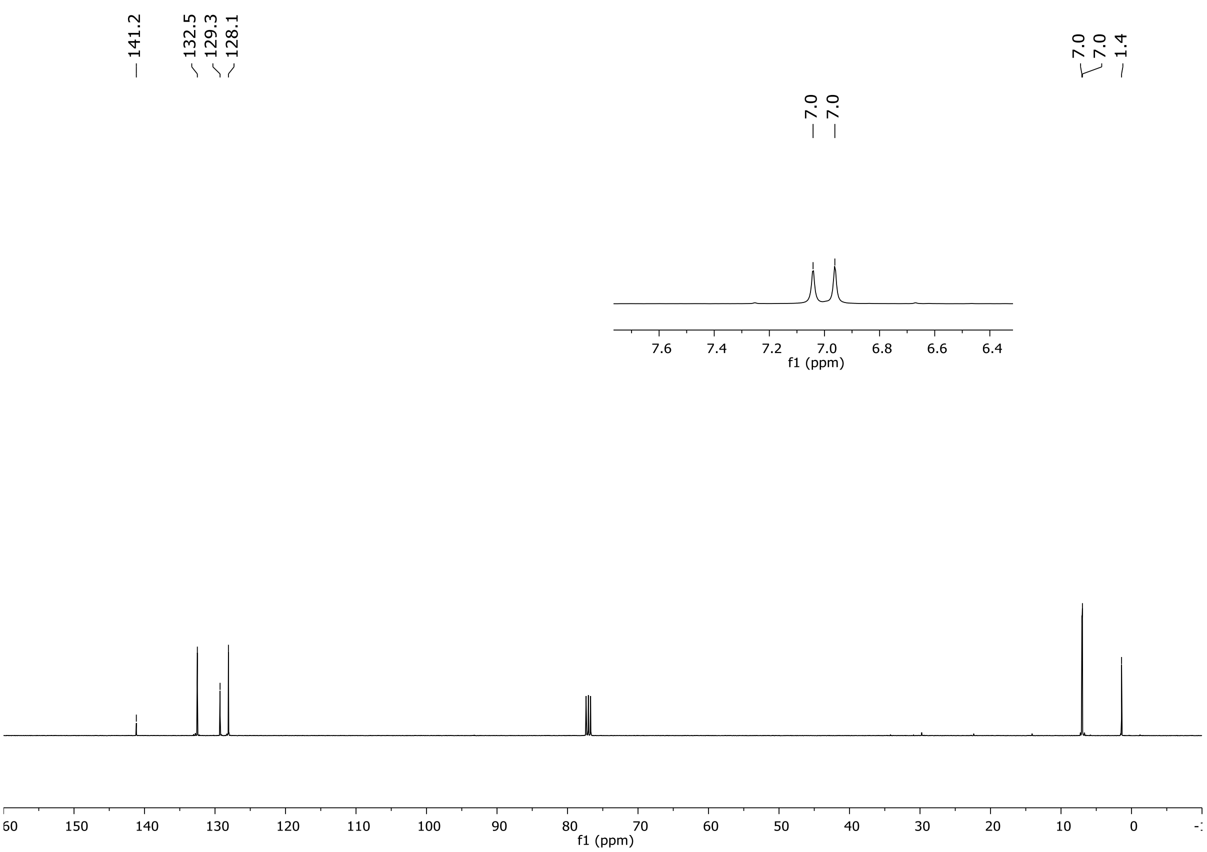

Figure S35. ^{13}C NMR Spectra of ((dimethyl(phenyl)germyl)oxy)triethylsilane (**3l**)

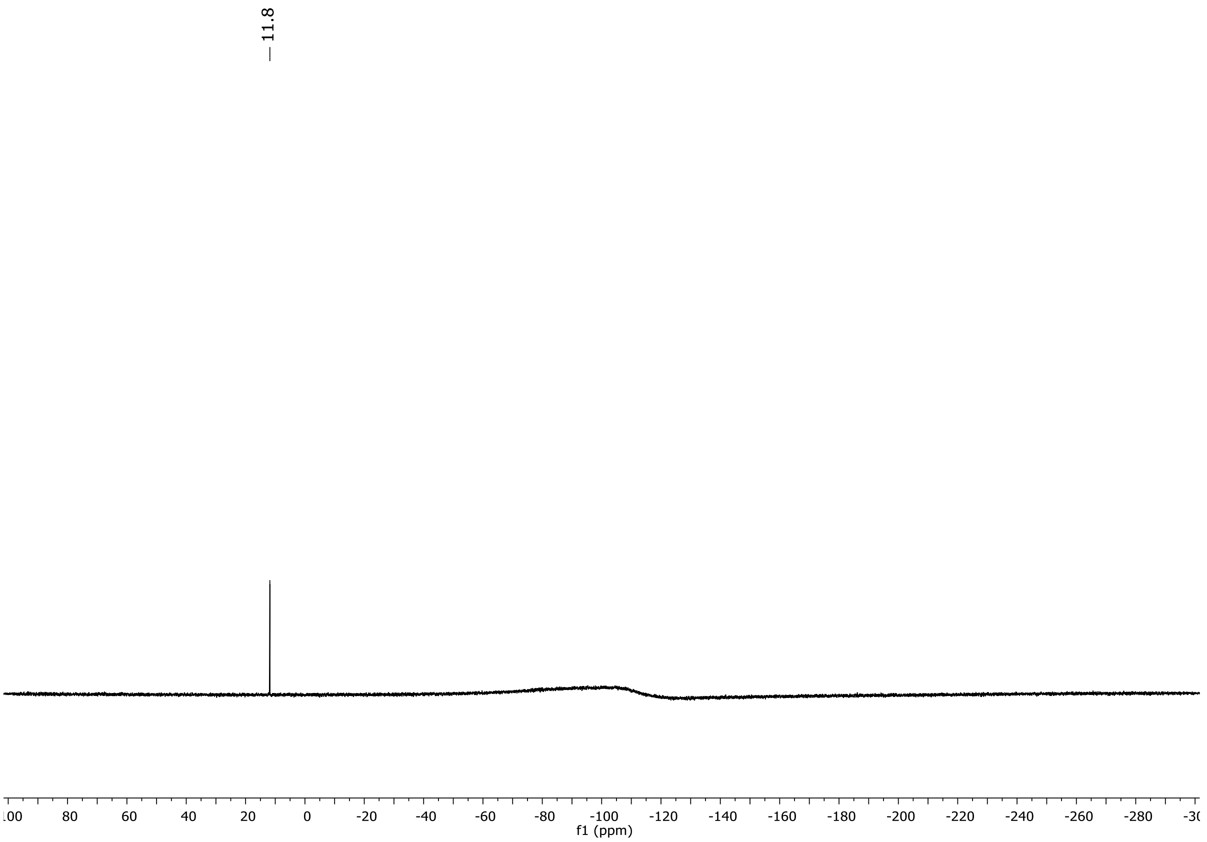

Figure S36. ^{29}Si NMR Spectra of ((dimethyl(phenyl)germyl)oxy)triethylsilane (**3l**)

Tert-butyl((dimethyl(phenyl)germyl)oxy)dimethylsilane (3m)

t-BuMe_2SiO—GeMe_2Ph

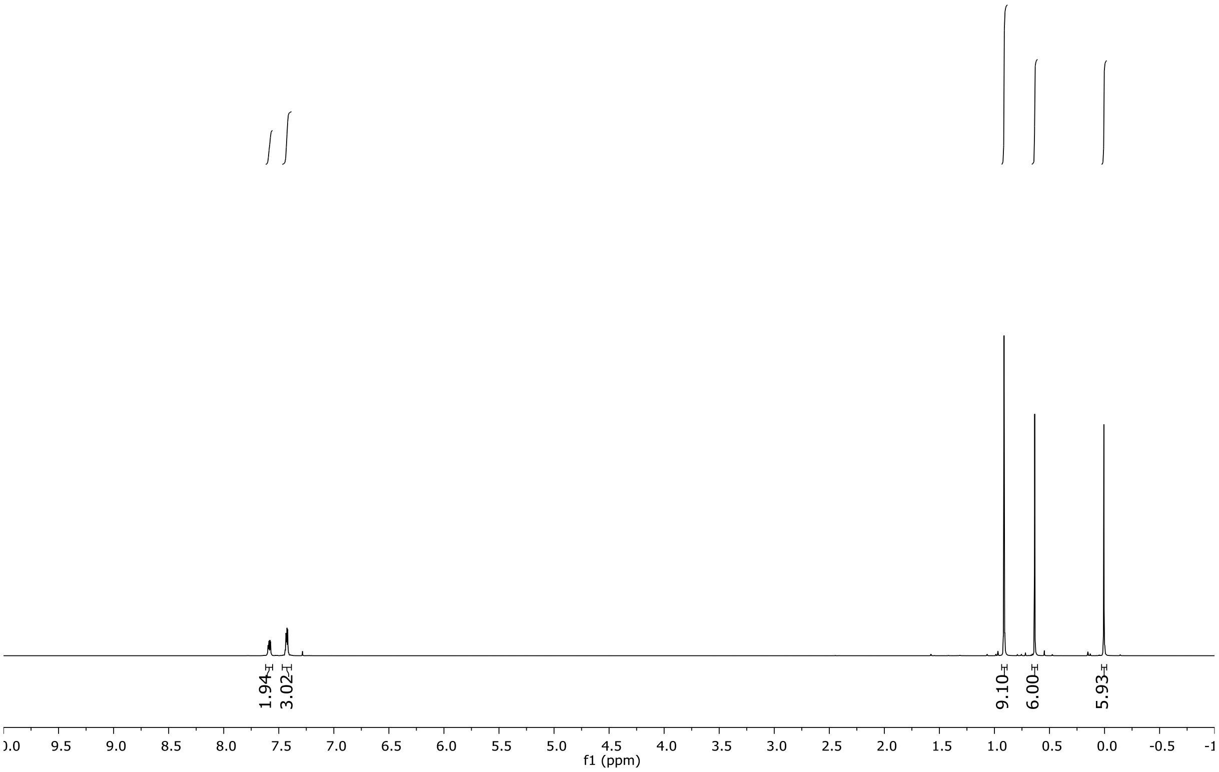

Figure S37. ^{1}H NMR Spectra of tert-butyl((dimethyl(phenyl)germyl)oxy)dimethylsilane (**3m**)

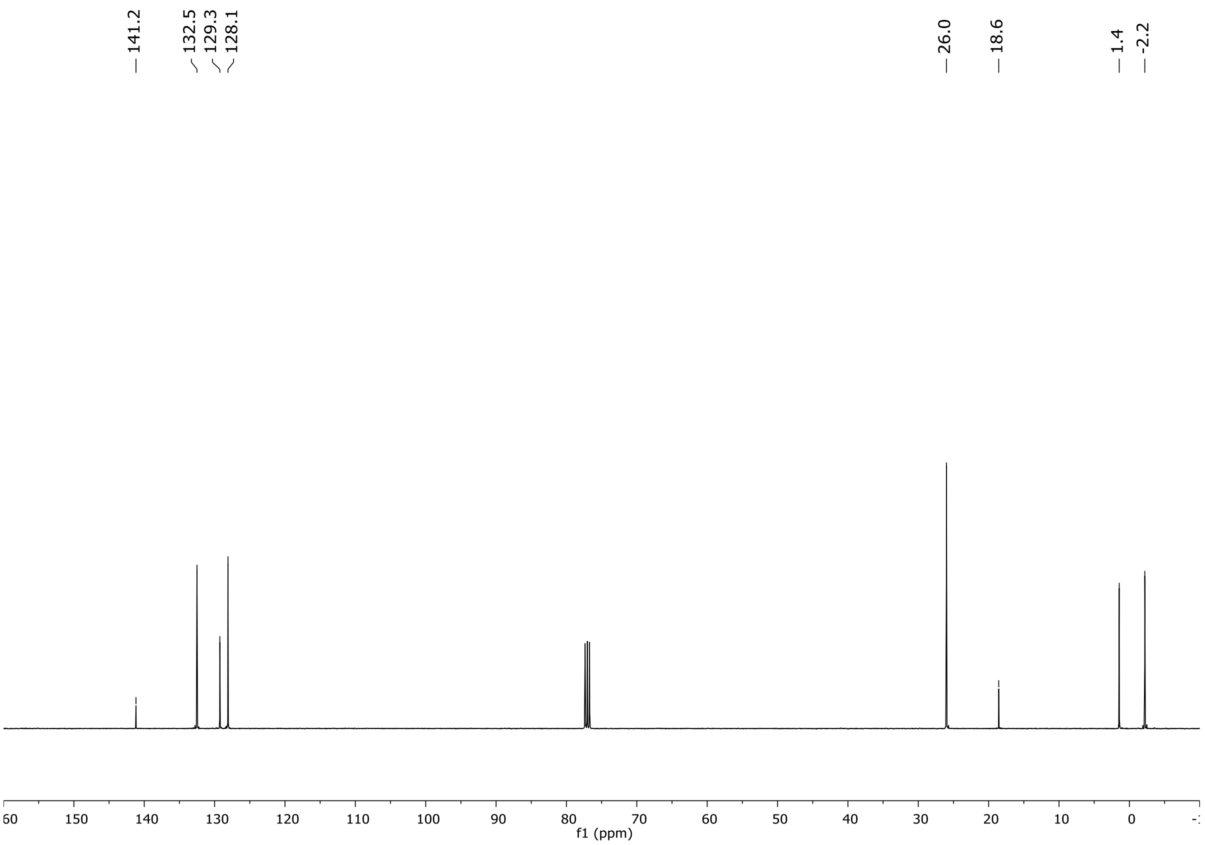

Figure S38. ^{13}C NMR Spectra of tert-butyl((dimethyl(phenyl)germyl)oxy)dimethylsilane (**3m**)

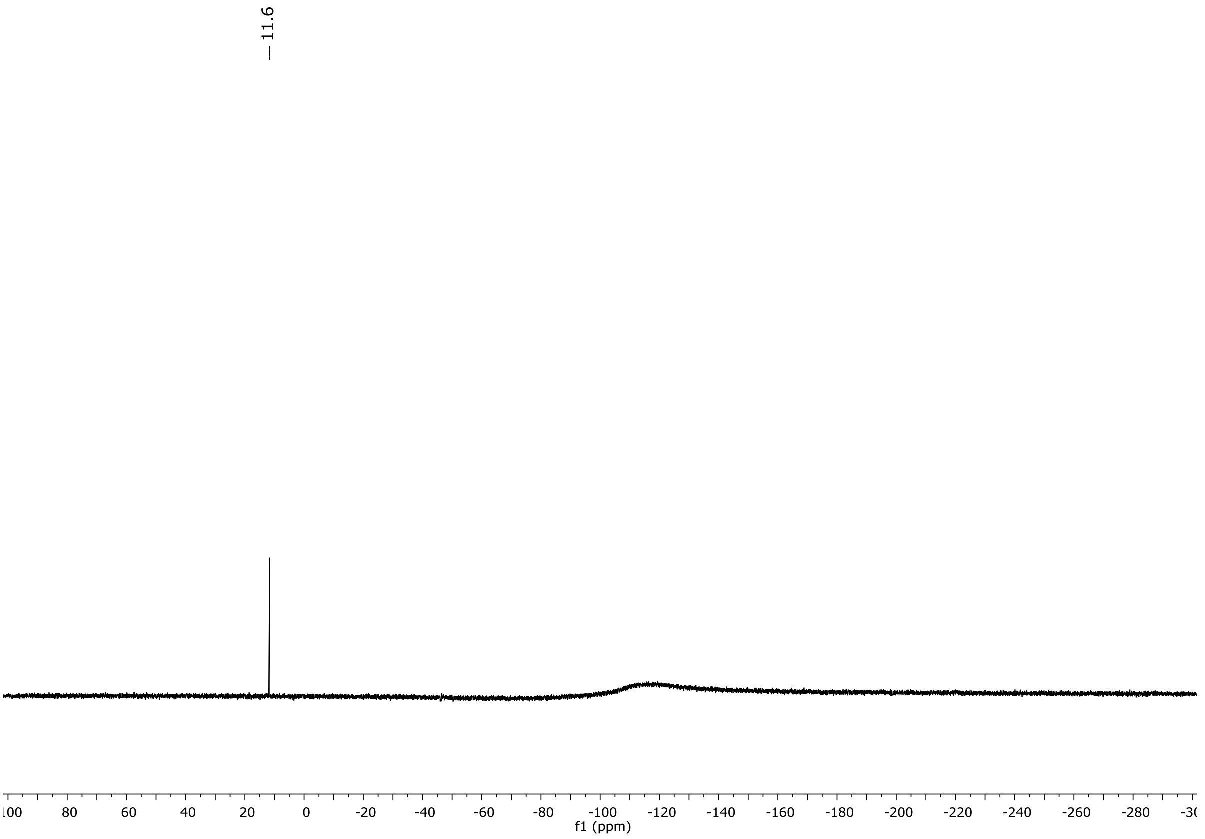

Figure S39. ^{29}Si NMR Spectra of tert-butyl((dimethyl(phenyl)germyl)oxy)dimethylsilane (**3m**)

((Dimethyl(phenyl)germyl)oxy)triisopropylsilane (3n)

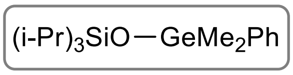

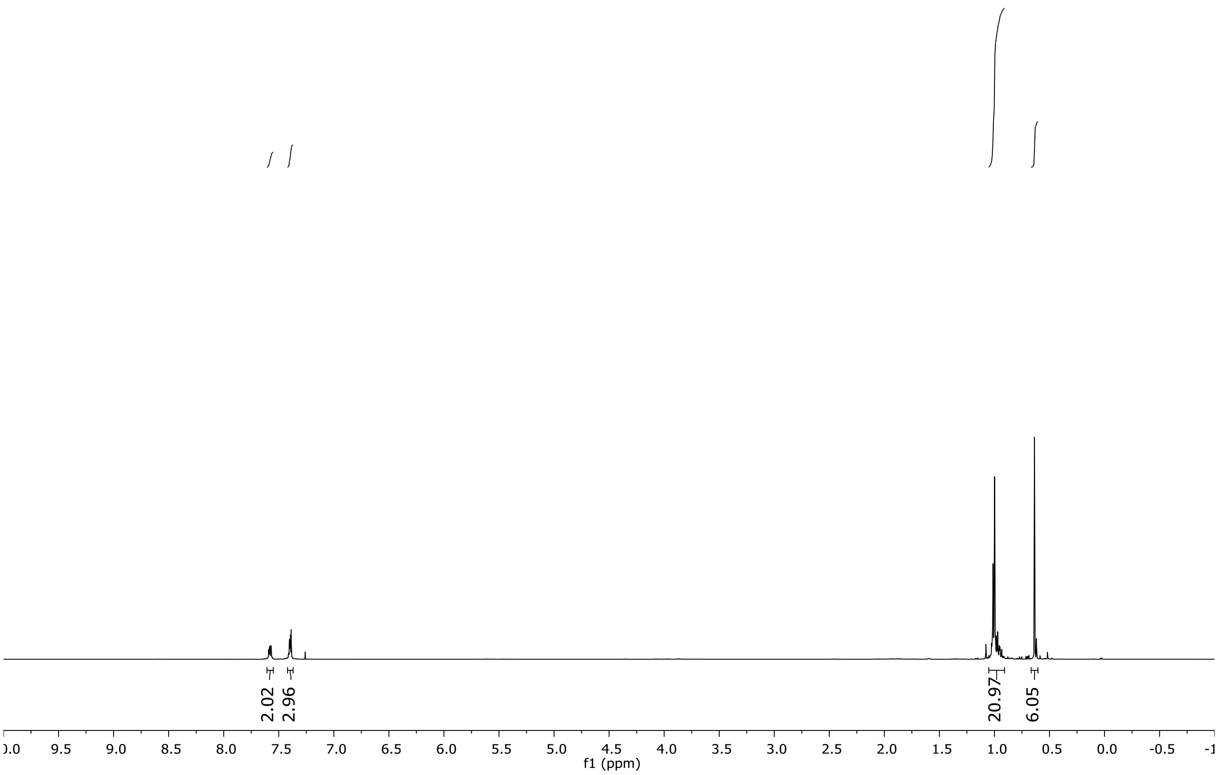

Figure S40. ^{1}H NMR Spectra of ((dimethyl(phenyl)germyl)oxy)triisopropylsilane (**3n**)

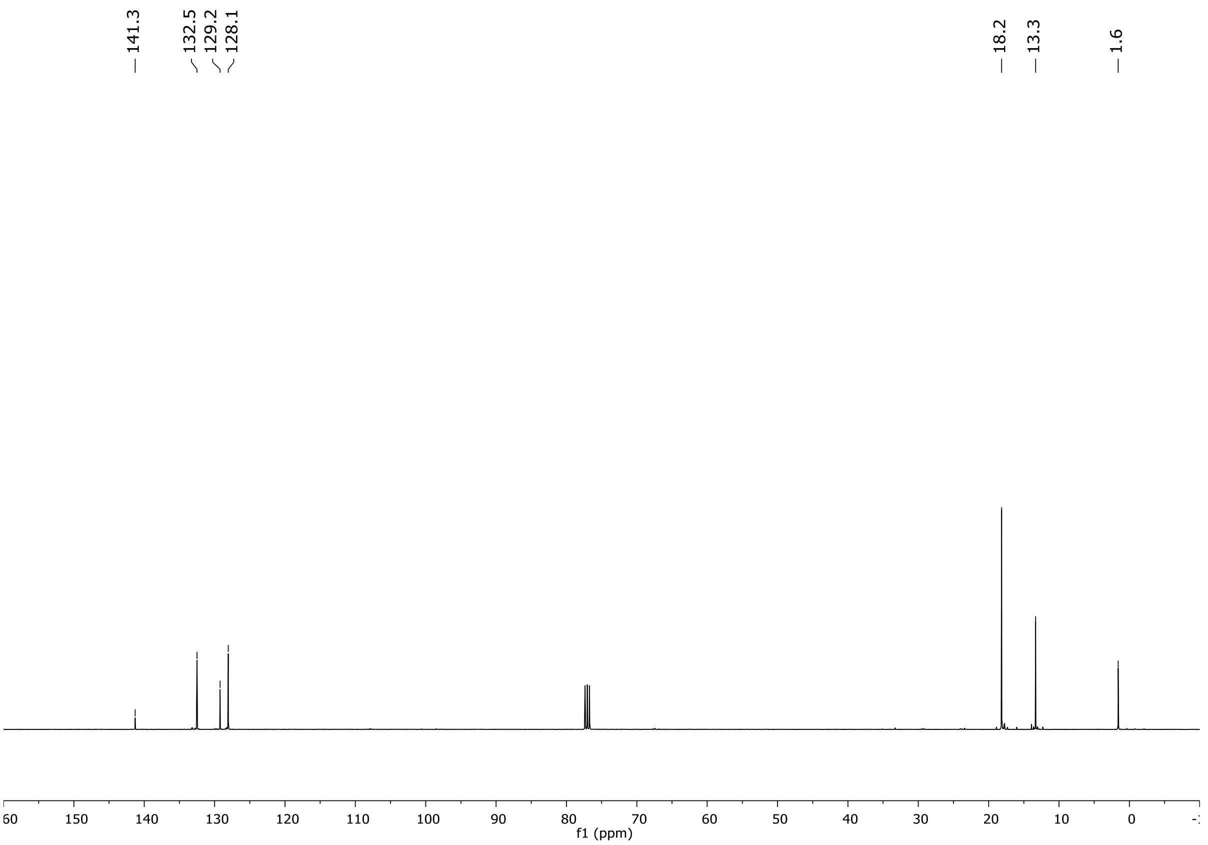

Figure S41. ^{13}C NMR Spectra of ((dimethyl(phenyl)germyl)oxy)triisopropylsilane (**3n**)

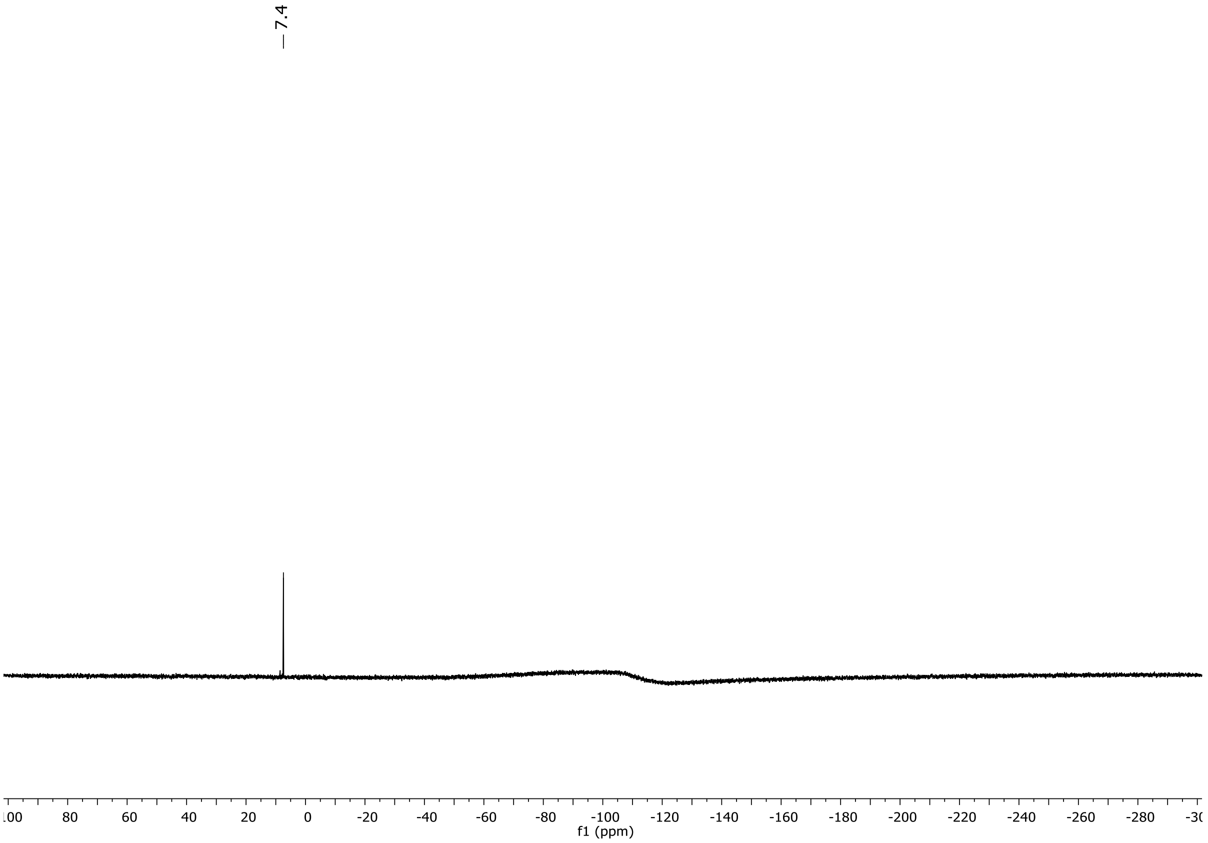

Figure S42. ^{29}Si NMR Spectra of ((dimethyl(phenyl)germyl)oxy)triisopropylsilane (**3n**)

Tert-butyldimethyl((triisopropylgermyl)oxy)silane (3o)

t-BuMe$_2$SiO—Ge(i-Pr)$_3$

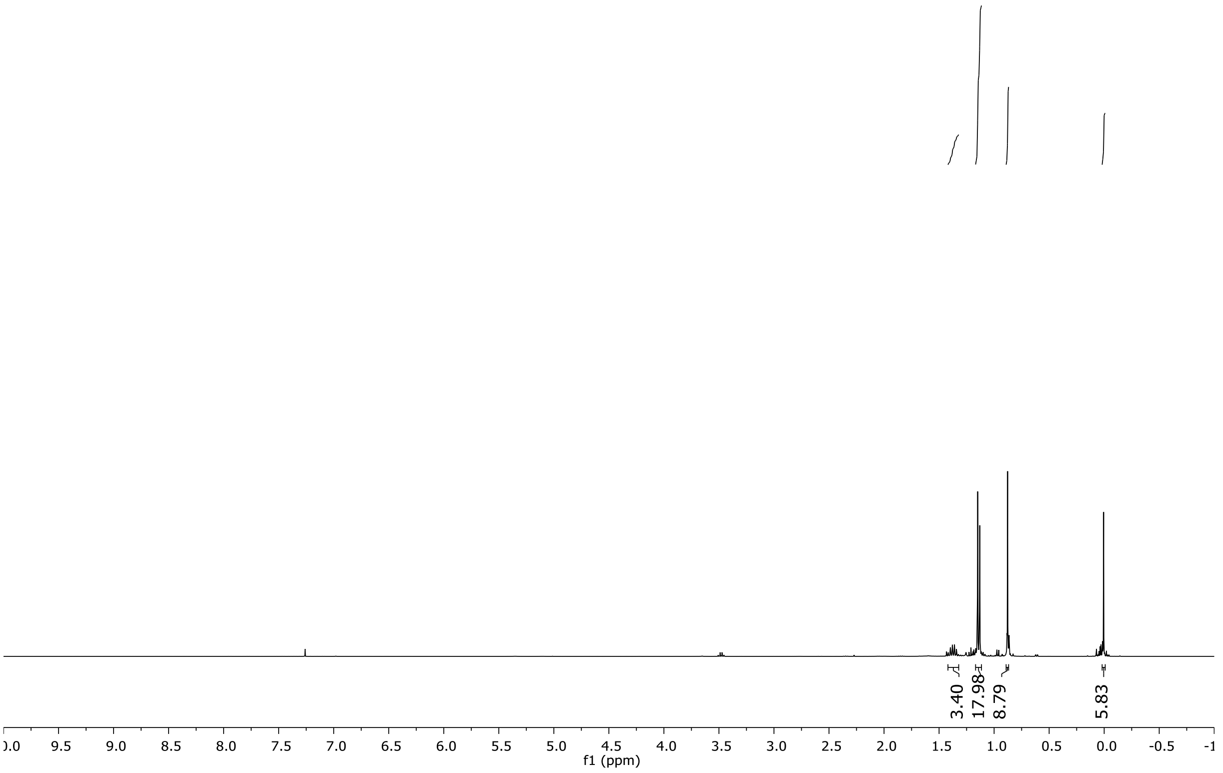

Figure S43. ^{1}H NMR Spectra of tert-butyldimethyl((triisopropylgermyl)oxy)silane (**3o**)

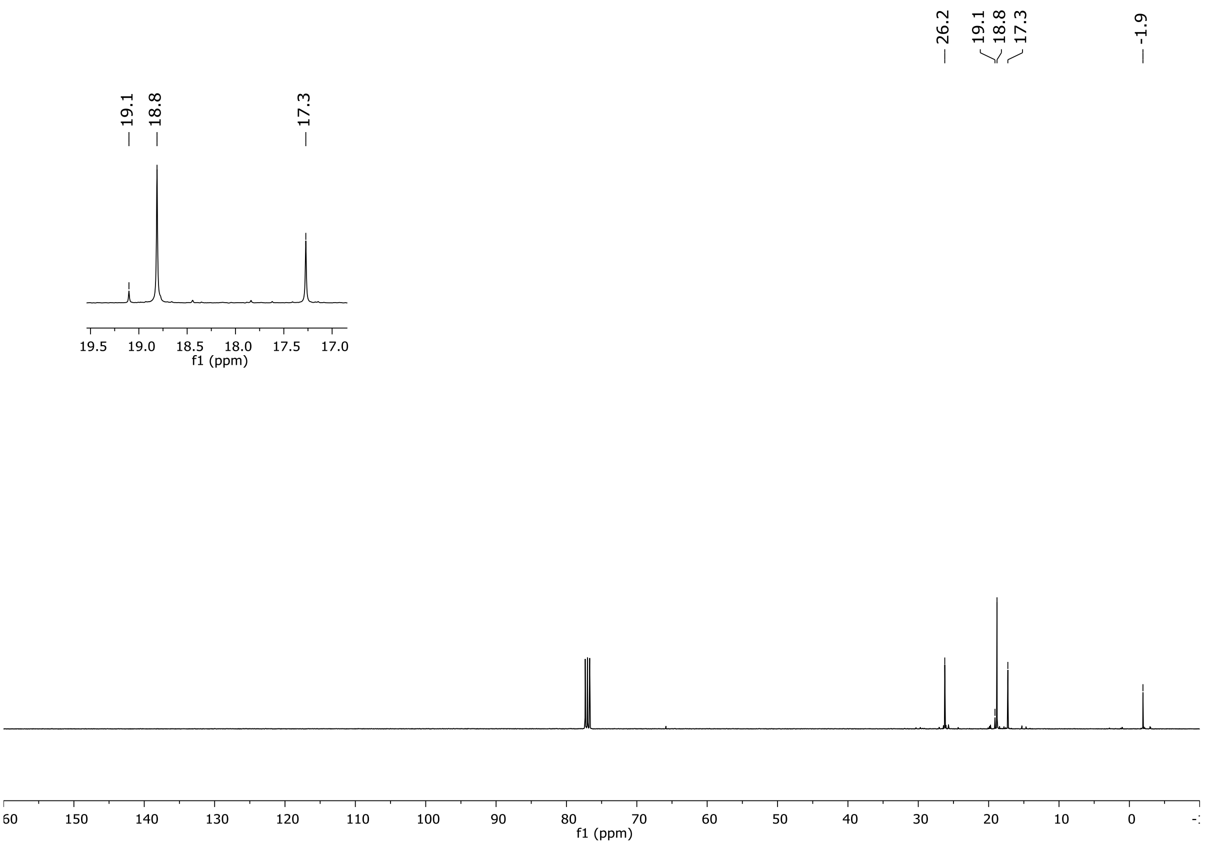

Figure S44. ^{13}C NMR Spectra of tert-butyldimethyl((triisopropylgermyl)oxy)silane (**3o**)

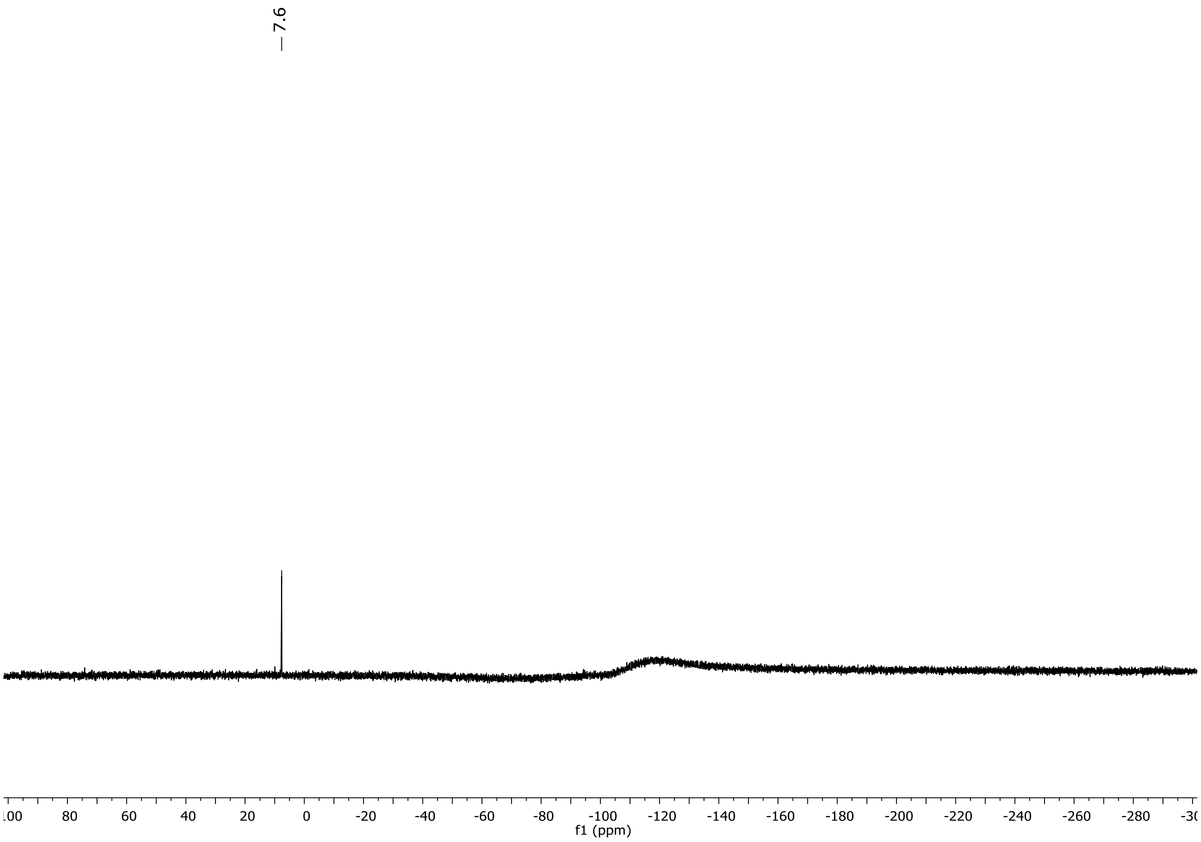

Figure S45. ^{29}Si NMR Spectra of tert-butyldimethyl((triisopropylgermyl)oxy)silane (**3o**)

Bis(tert-butyldimethylsiloxy)diethylgermane (3p)

t-BuMe$_2$SiO—GeEt$_2$—OSiMe$_2$t-Bu

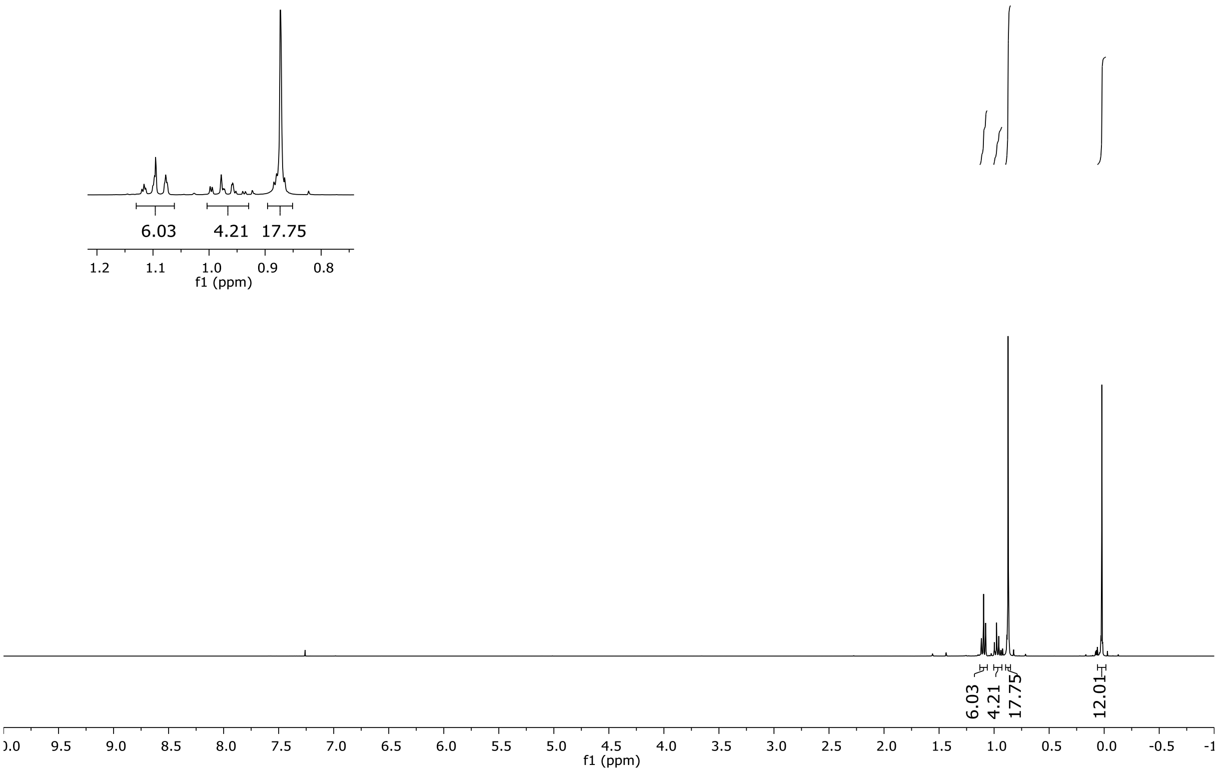

Figure S46. ^{1}H NMR Spectra of bis(tert-butyldimethylsiloxy)diethylgermane (**3p**)

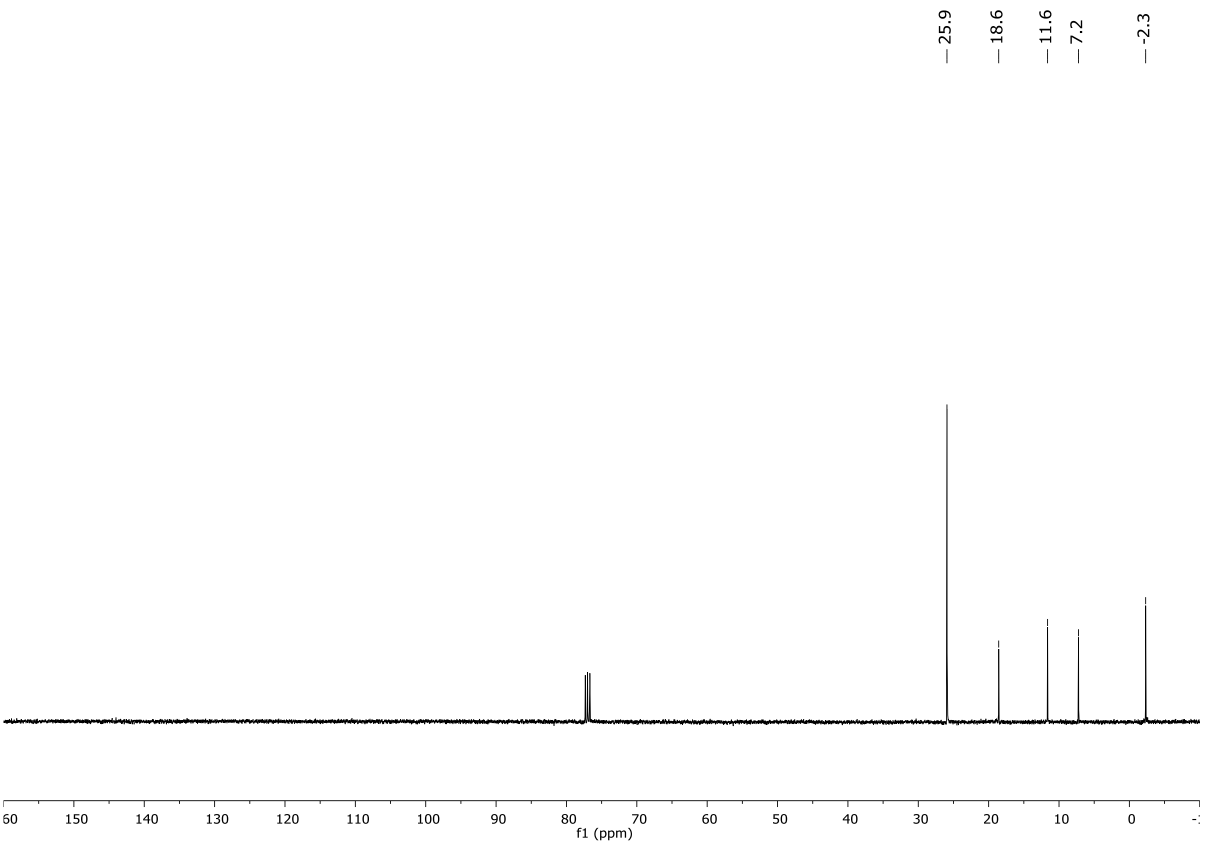

Figure S47. ^{13}C NMR Spectra of bis(tert-butyldimethylsiloxy)diethylgermane (**3p**)

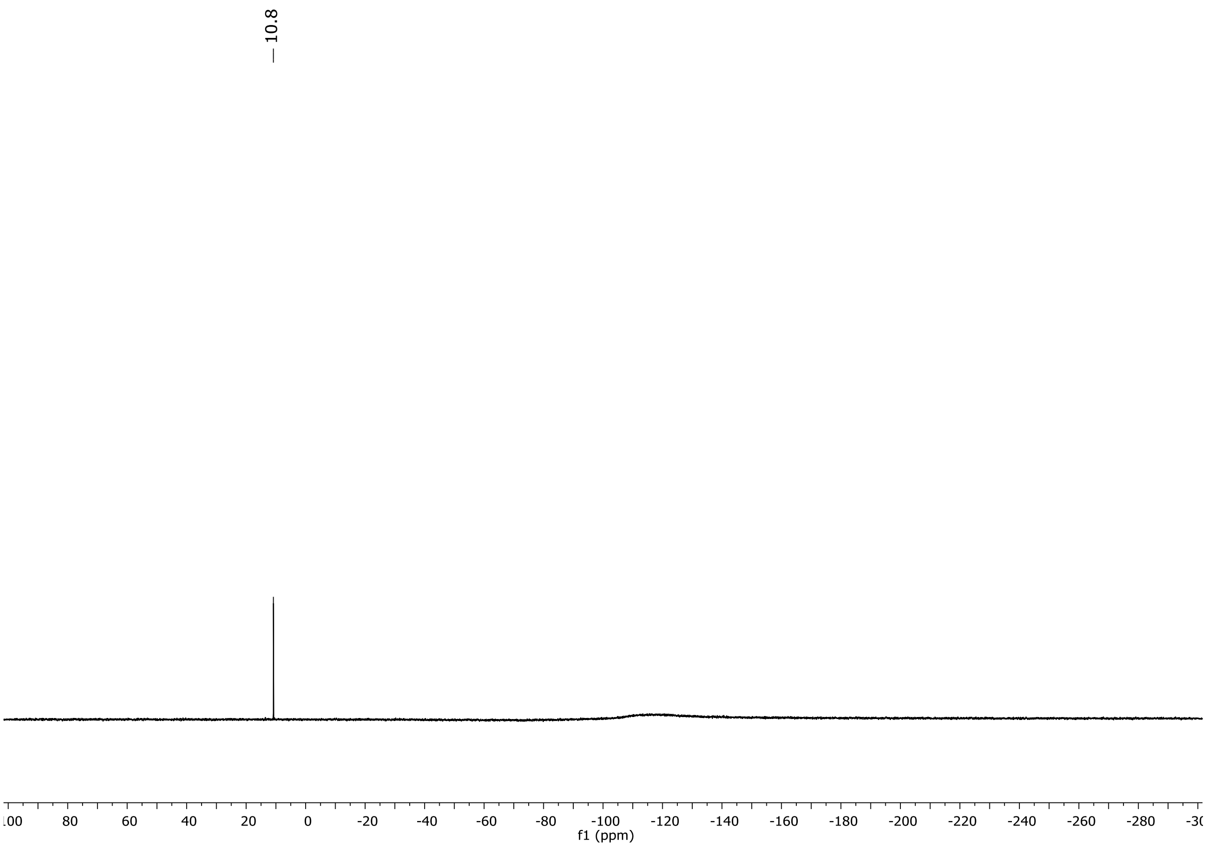

Figure S48. ^{29}Si NMR Spectra of bis(tert-butyldimethylsiloxy)diethylgermane (**3p**)

Tert-butyl((dibutyl(ethynyl)germyl)oxy)dimethylsilane (3q)

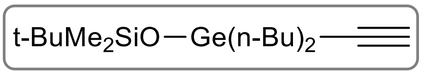

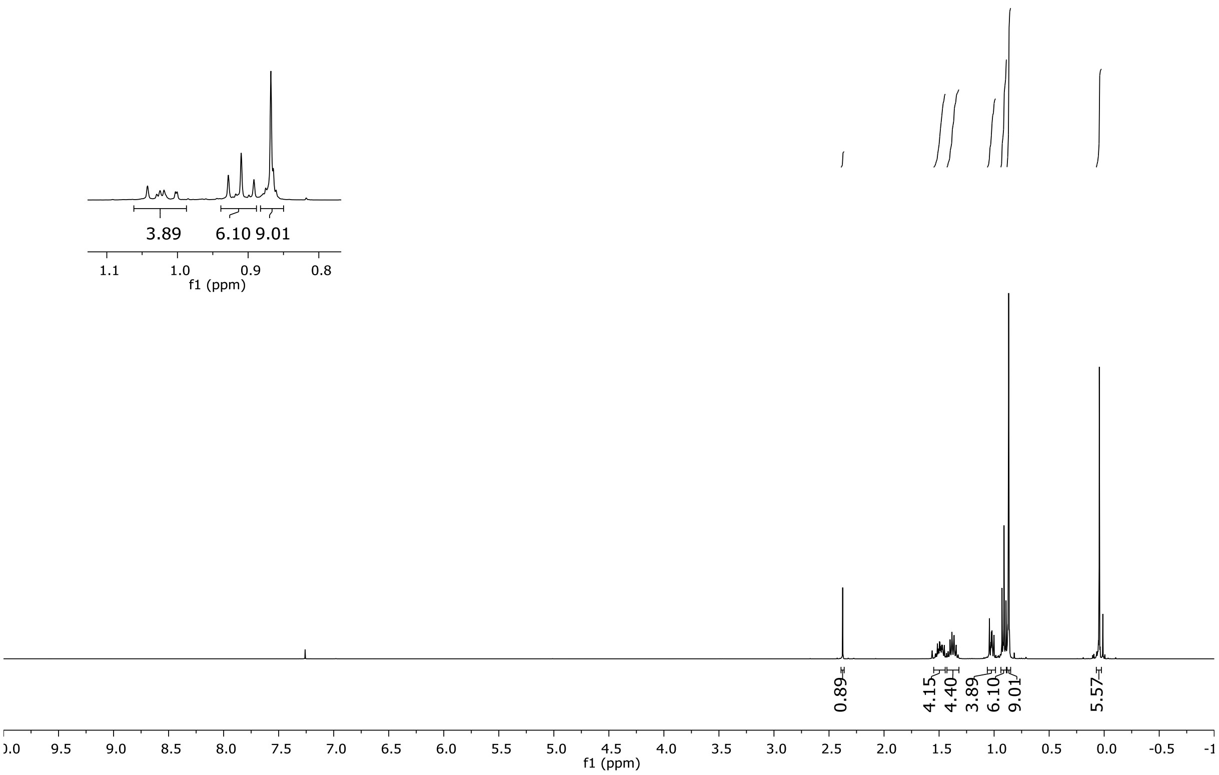

Figure S49. ^{1}H NMR Spectra of tert-butyl((dibutyl(ethynyl)germyl)oxy)dimethylsilane (**3q**)

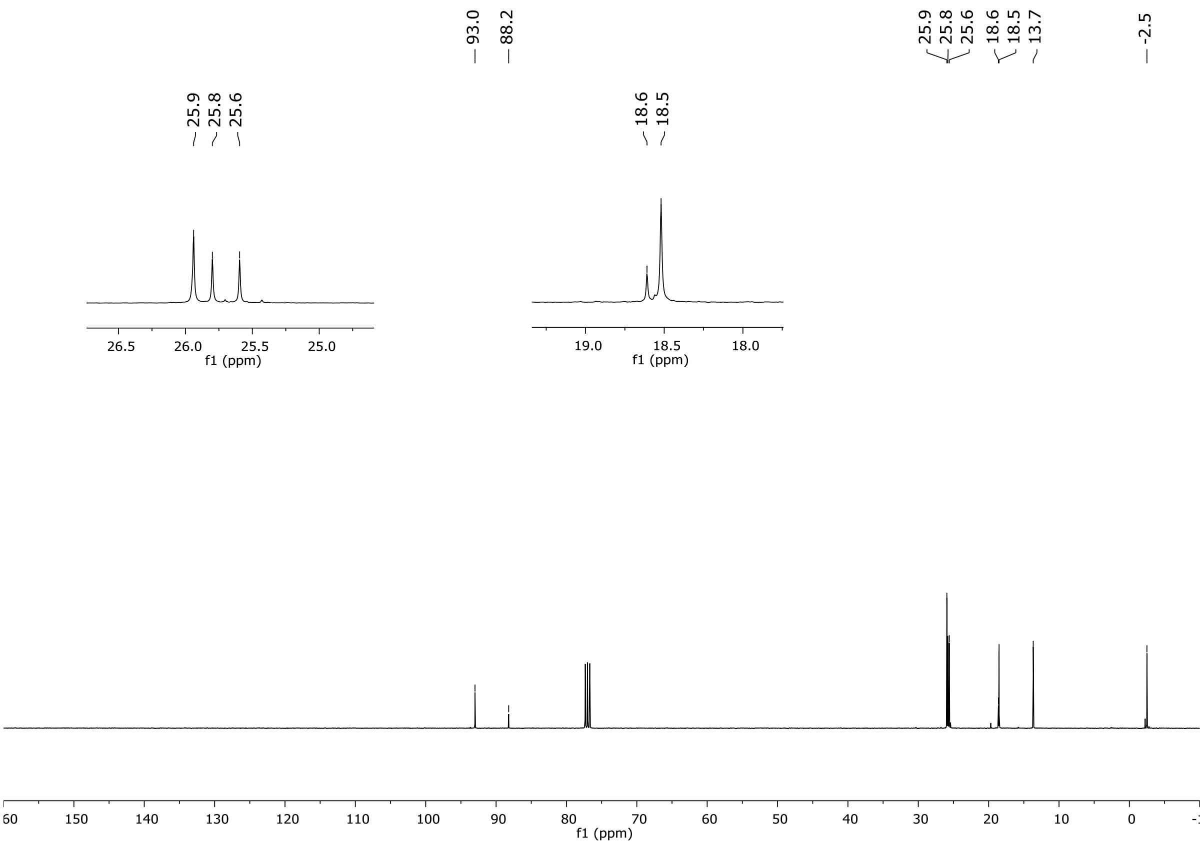

Figure S50. ^{13}C NMR Spectra of tert-butyl((dibutyl(ethynyl)germyl)oxy)dimethylsilane (**3q**)

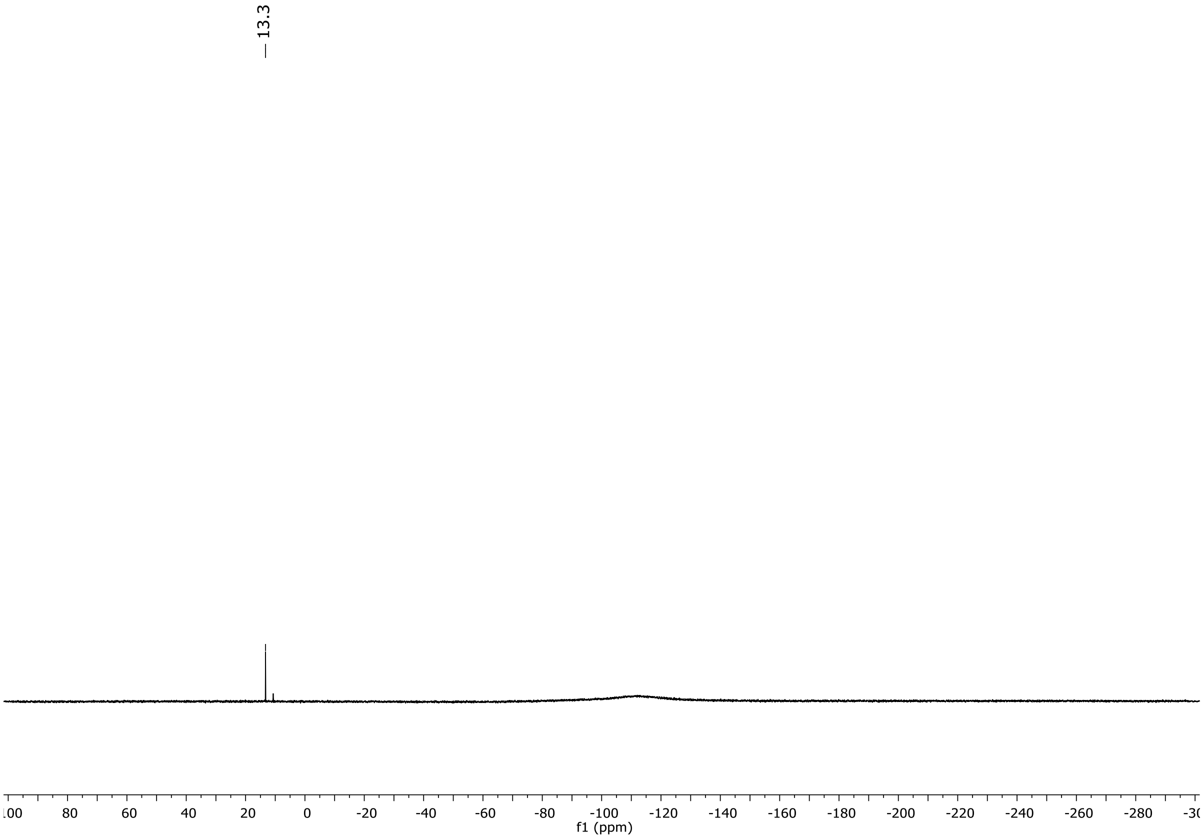

Figure S51. ^{29}Si NMR Spectra of tert-butyl((dibutyl(ethynyl)germyl)oxy)dimethylsilane (**3q**)

Bis(tert-butyldimethylsiloxy)dibutylgermane (3r)

$t\text{-BuMe}_2\text{SiO}—\text{Ge}(n\text{-Bu})_2—\text{OSiMe}_2t\text{-Bu}$

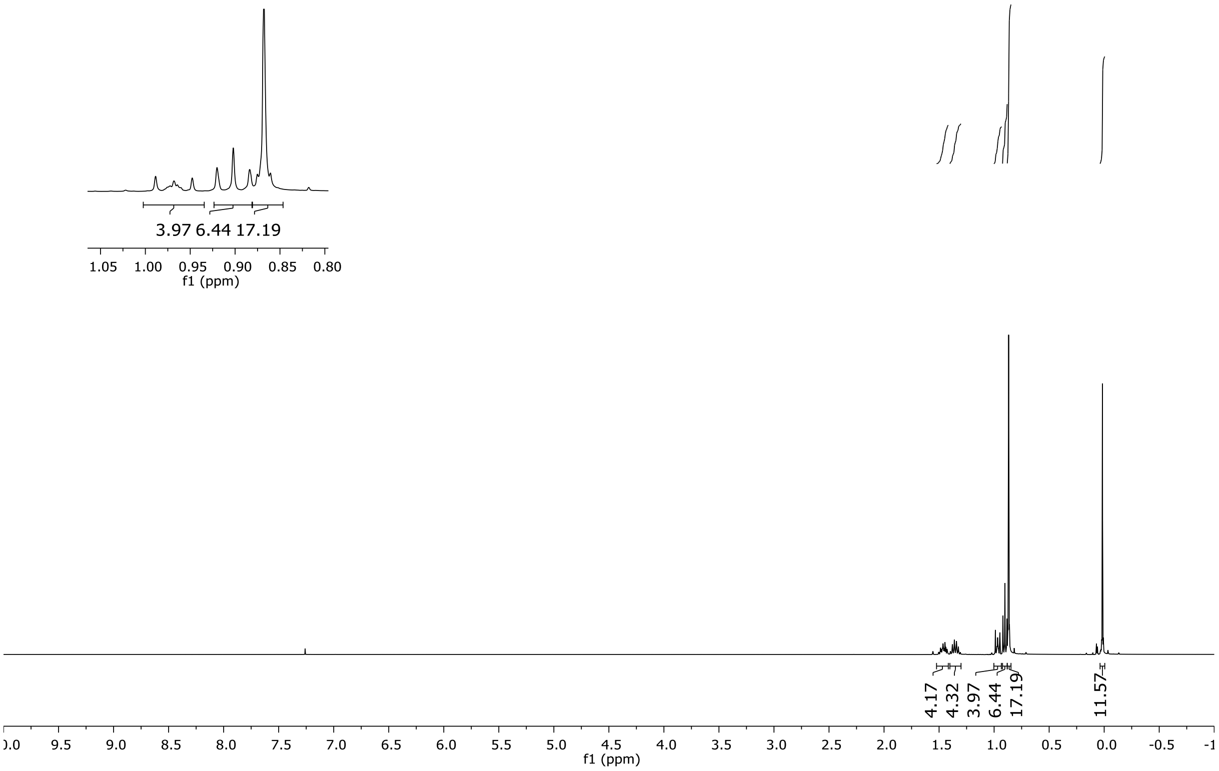

Figure S52. ^{1}H NMR Spectra of bis(tert-butyldimethylsiloxy)dibutylgermane (**3r**)

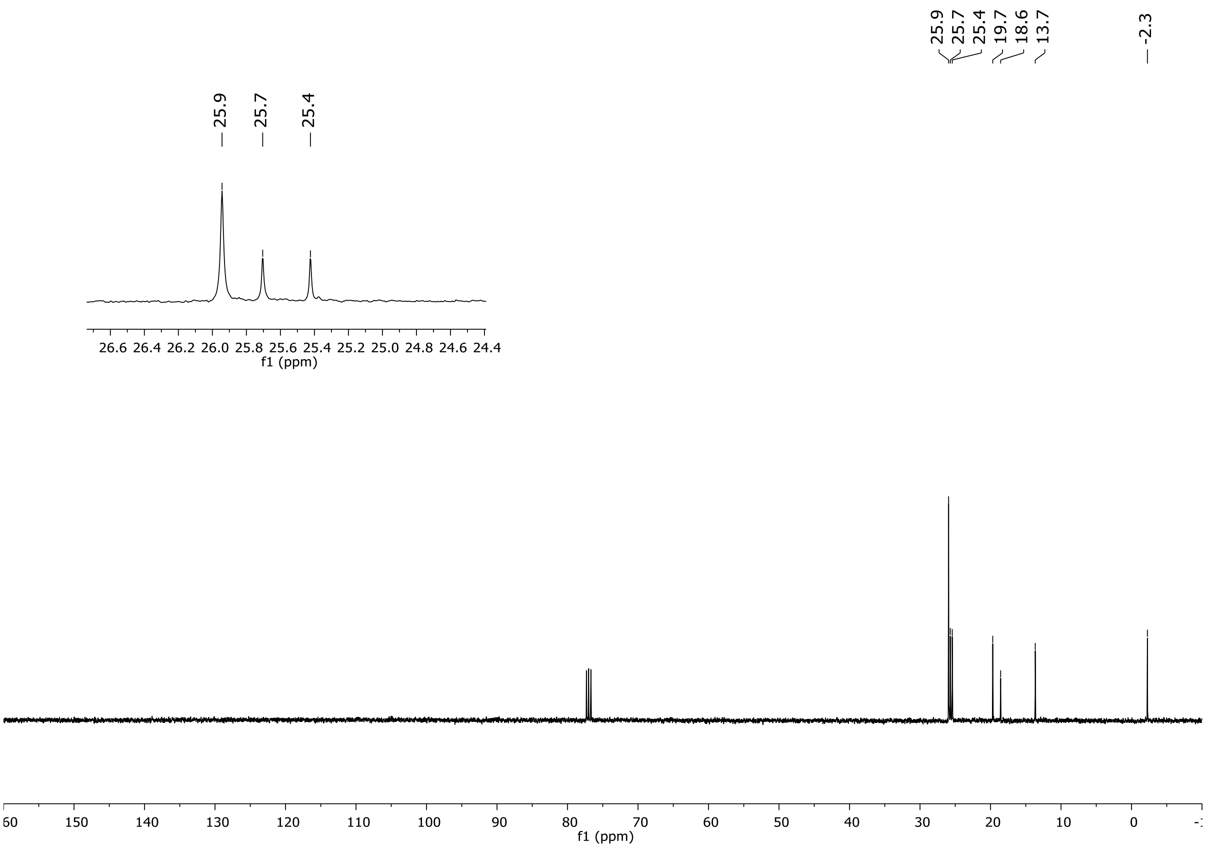

Figure S53. ^{13}C NMR Spectra of bis(tert-butyldimethylsiloxy)dibutylgermane (**3r**)

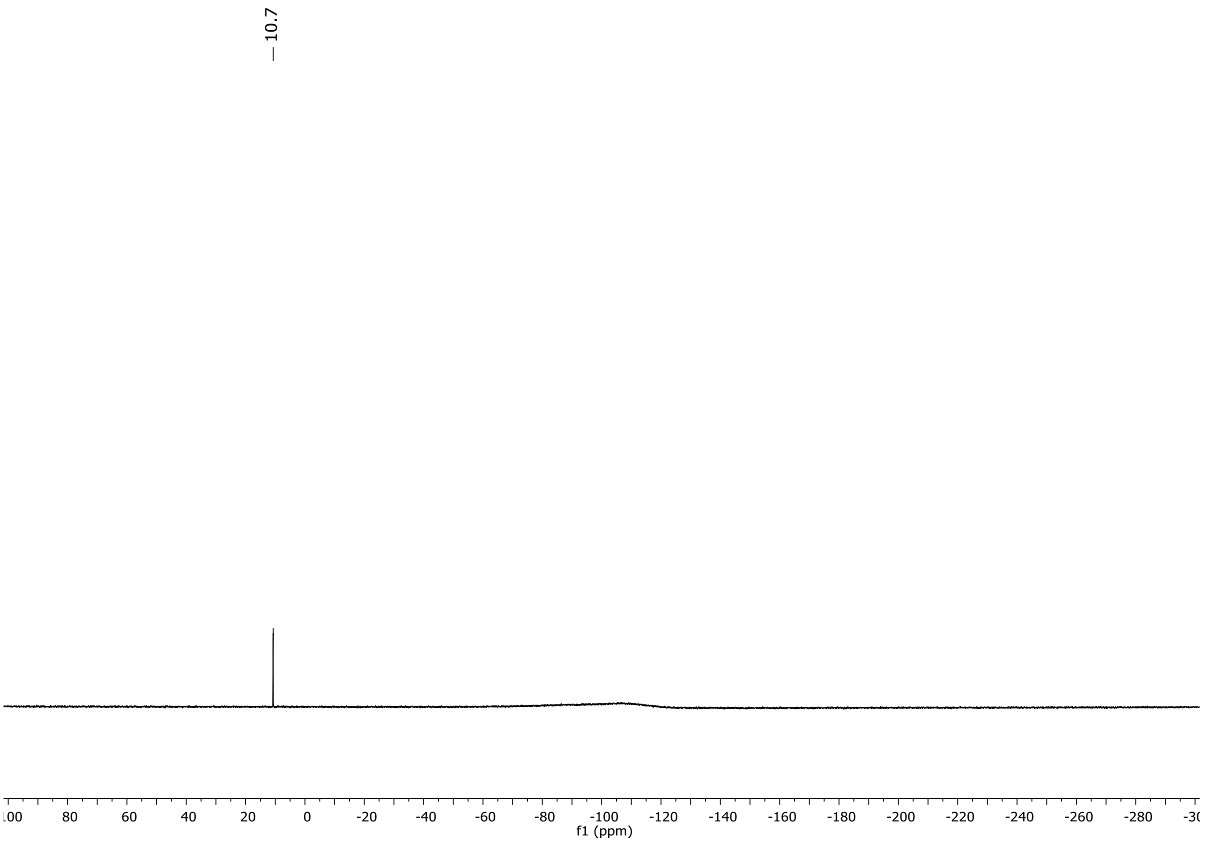

Figure S54. ^{29}Si NMR Spectra of bis(tert-butyldimethylsiloxy)dibutylgermane (**3r**)

(1-(4-Chlorophenyl)ethoxy)triethylgermane (3s)

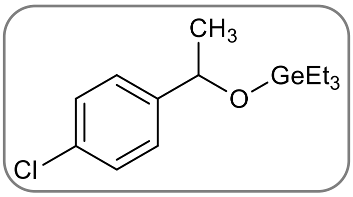

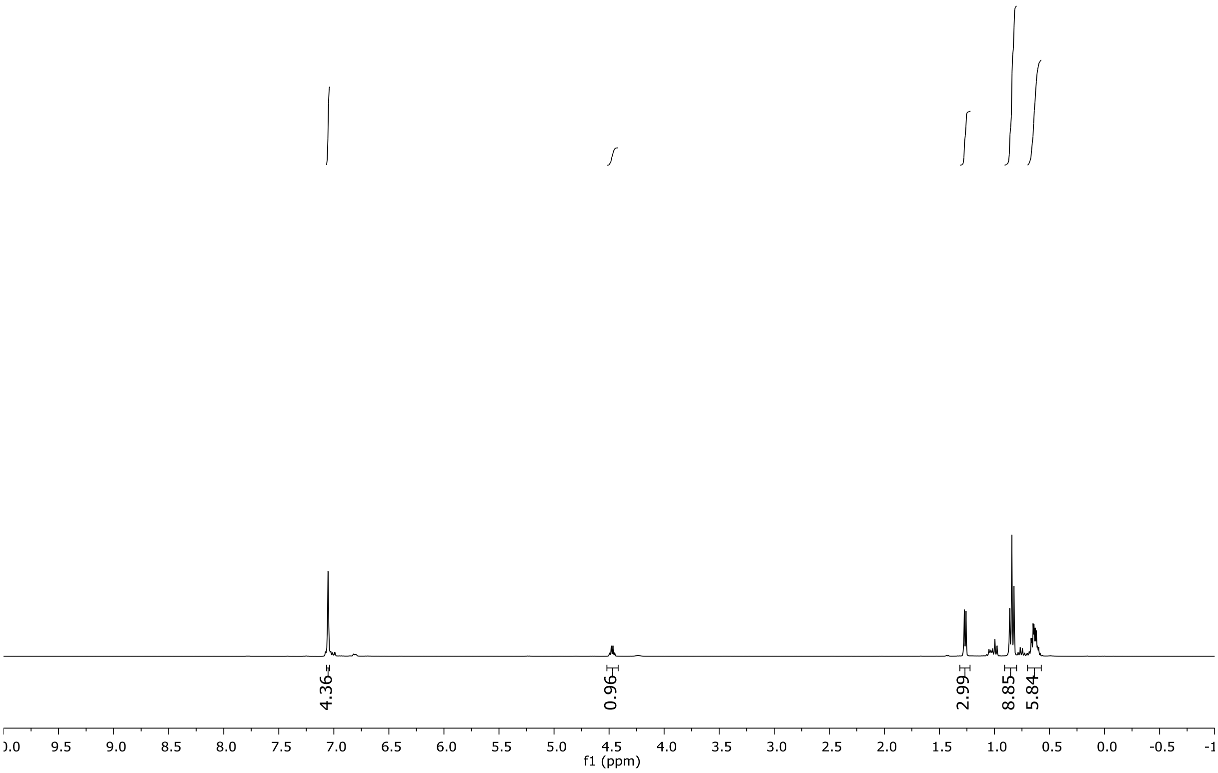

Figure S55. ^{1}H NMR Spectra of (1-(4-chlorophenyl)ethoxy)triethylgermane (**3s**)

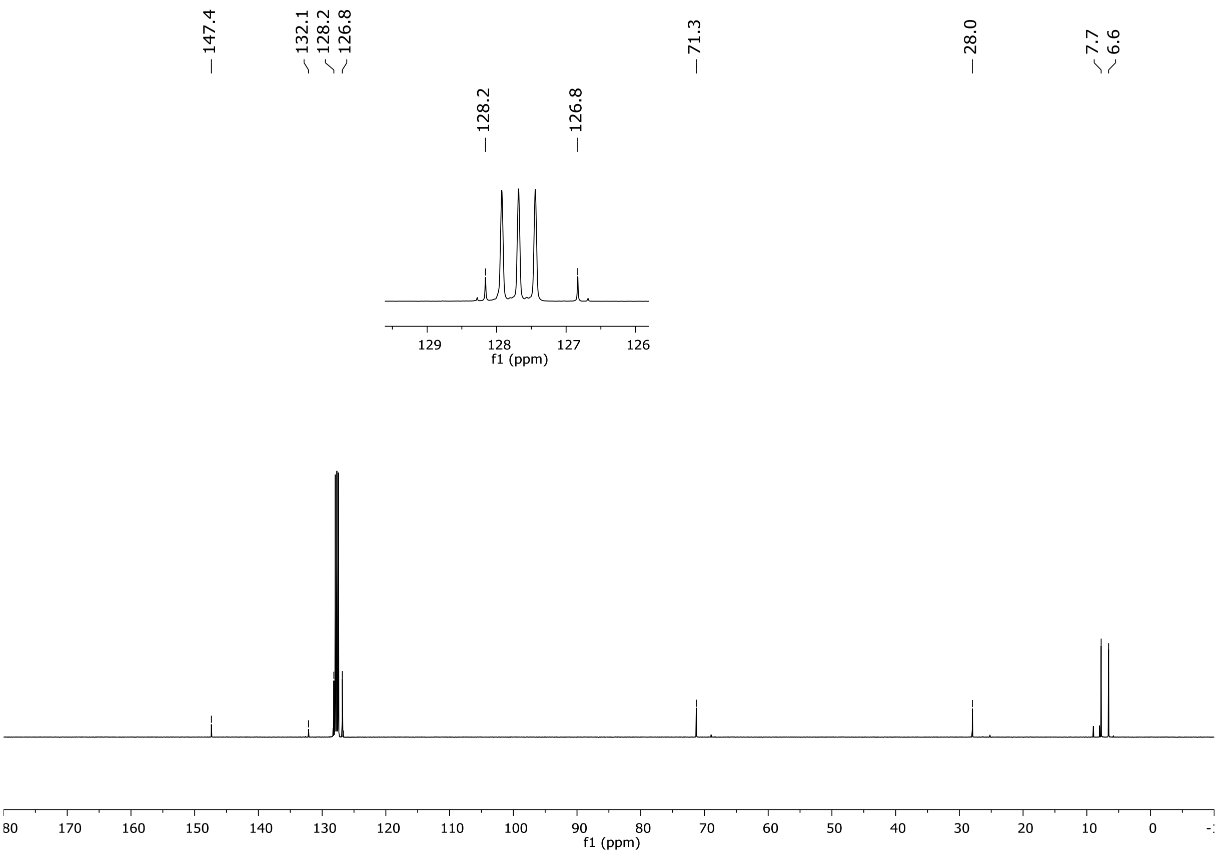

Figure S56. ^{13}C NMR Spectra of (1-(4-chlorophenyl)ethoxy)triethylgermane (**3s**)

Tributyl(isopropoxy)germane (3t)

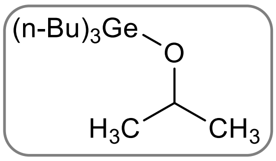

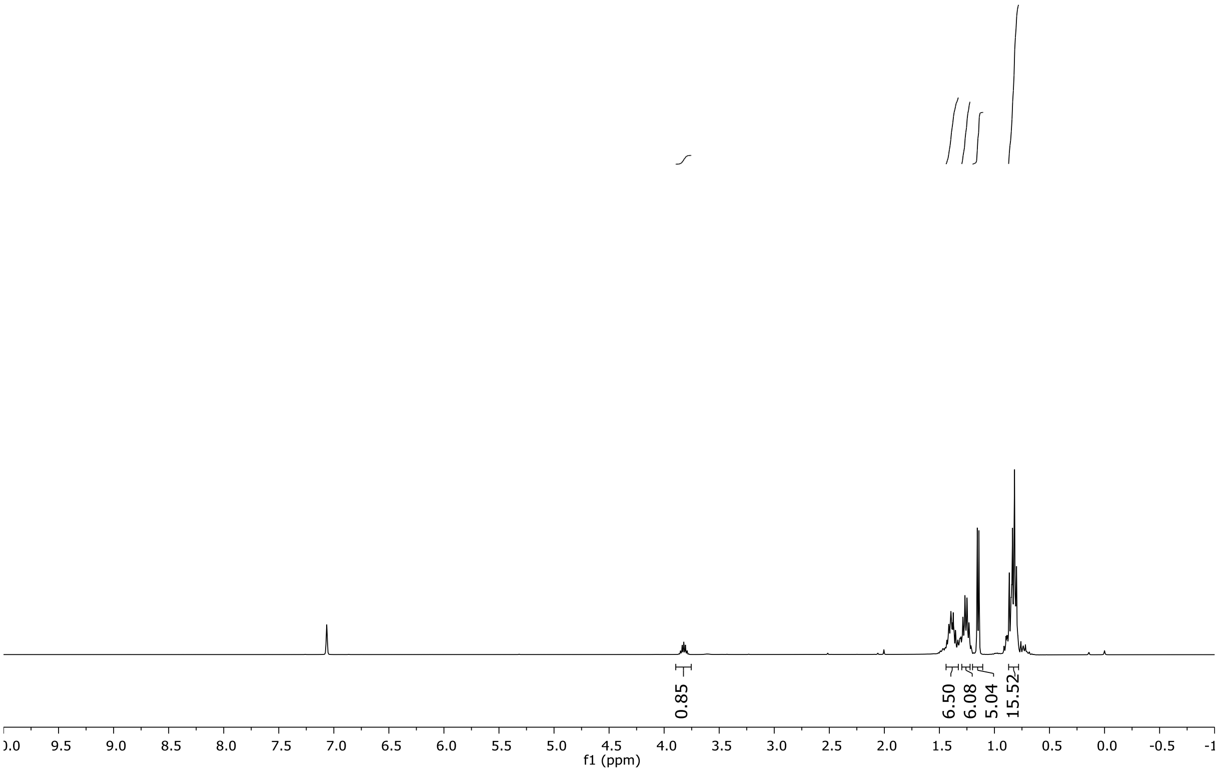

Figure S57. ^{1}H NMR Spectra of tributyl(isopropoxy)germane (**3t**)

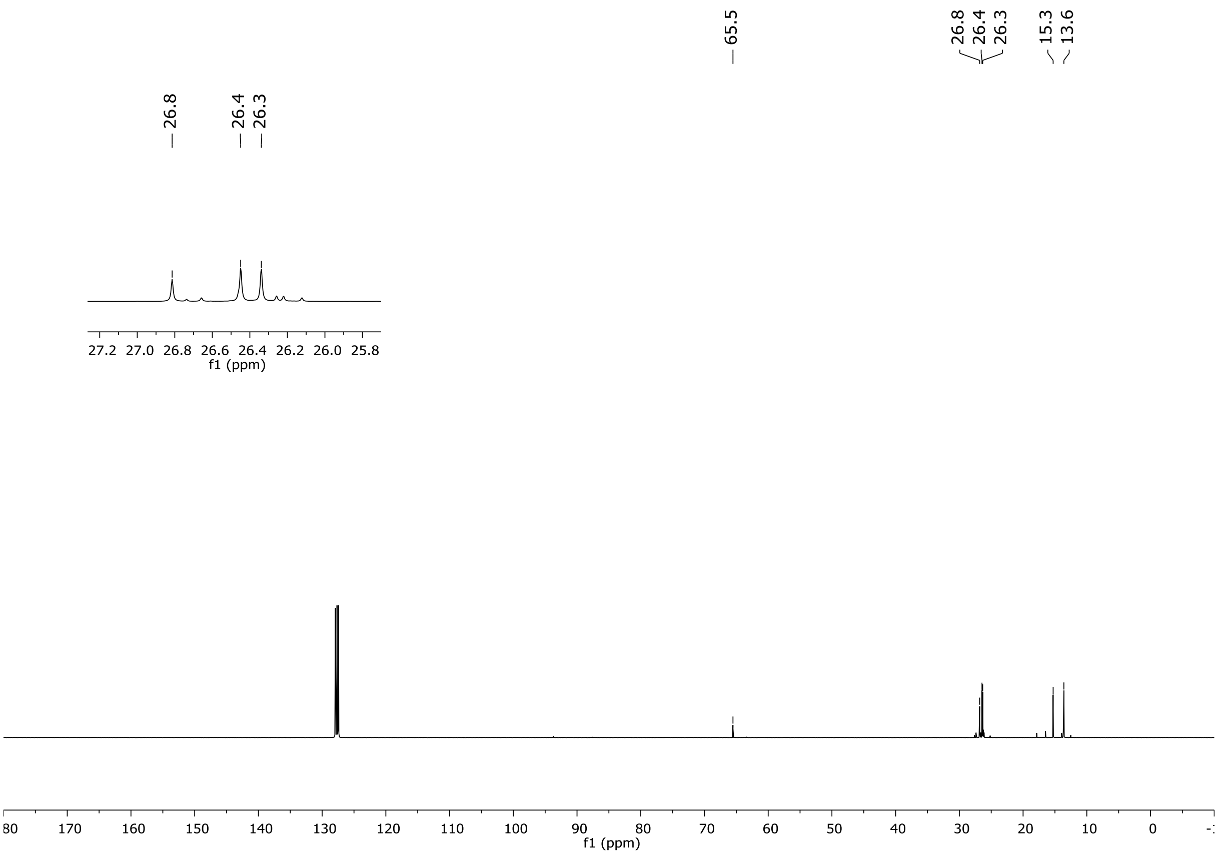

Figure S58. ^{13}C NMR Spectra of tributyl(isopropoxy)germane (**3t**)

1,2-Bis(triethylgermyl)ethyne (4a)

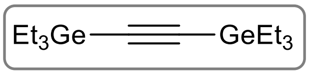

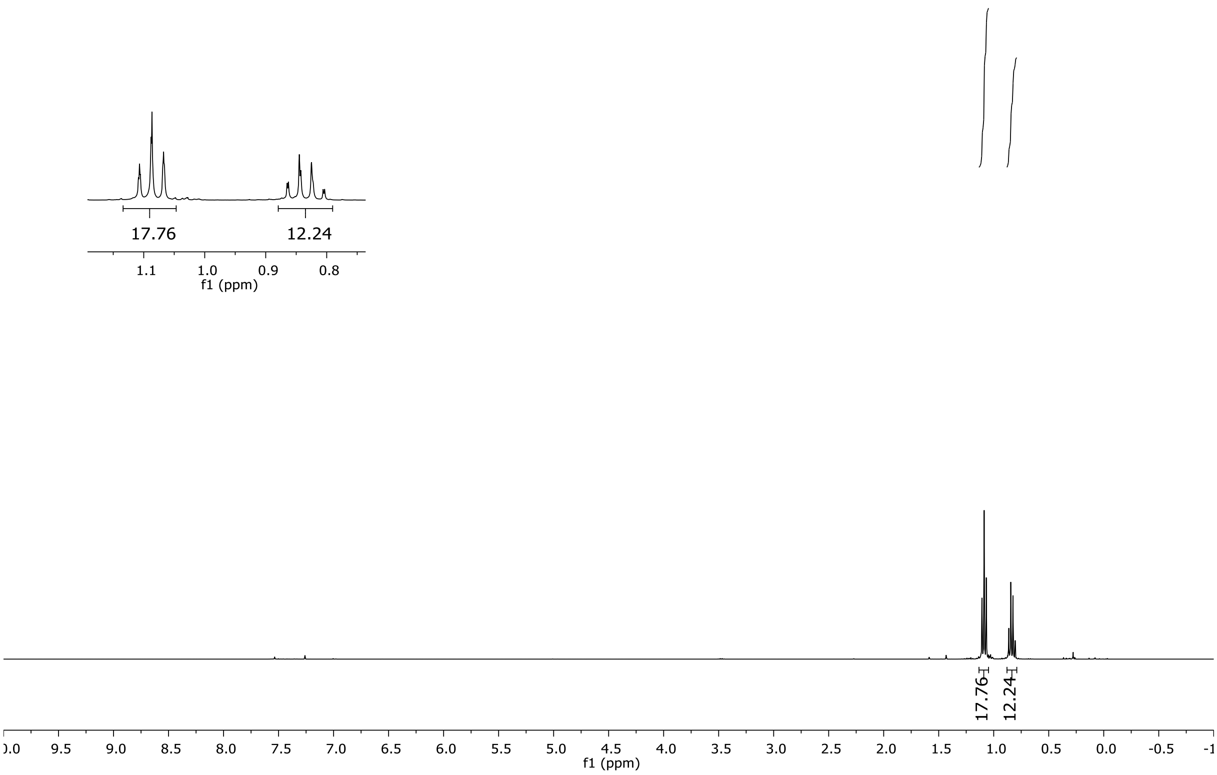

Figure S59. ^{1}H NMR Spectra of 1,2-bis(triethylgermyl)ethyne (**4a**)

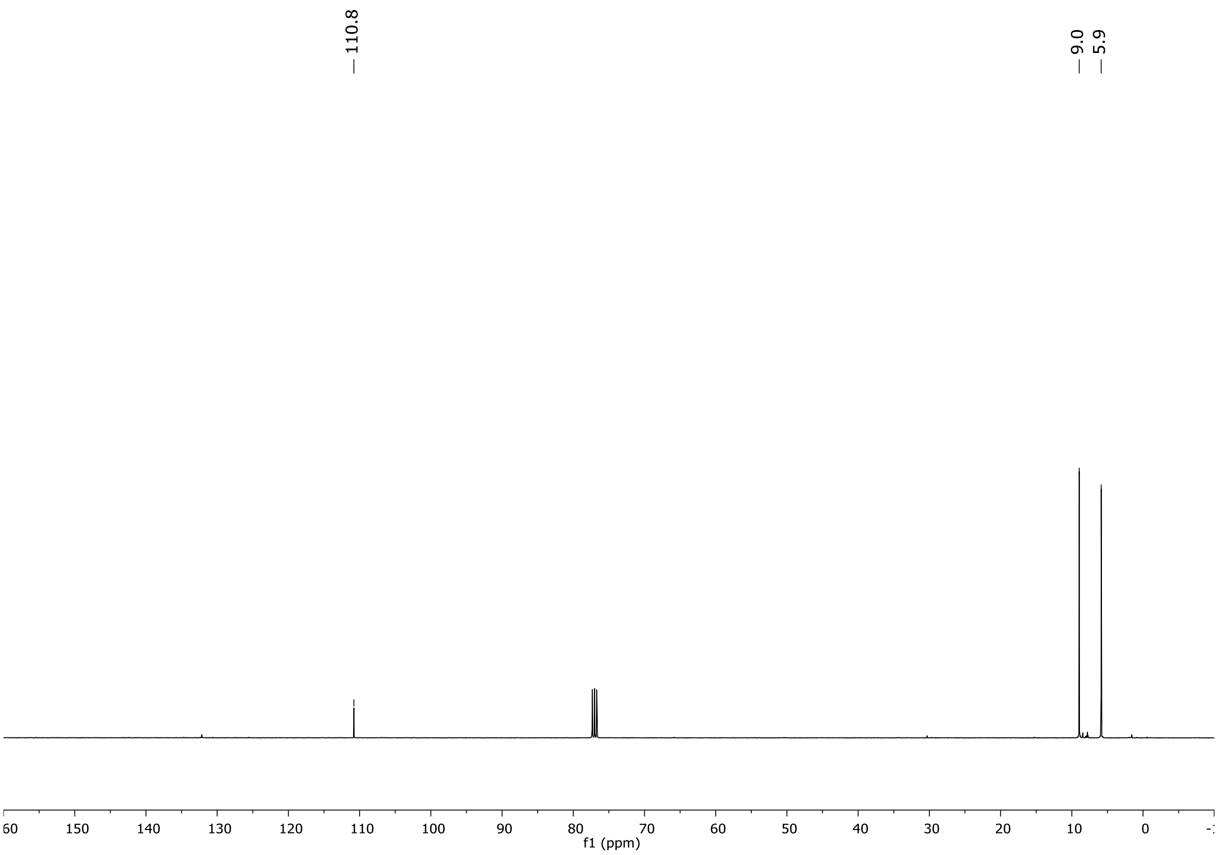

Figure S60. ^{13}C NMR Spectra of 1,2-bis(triethylgermyl)ethyne (**4a**)

1,2-Bis(tributylgermyl)ethyne (4b)

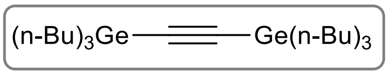

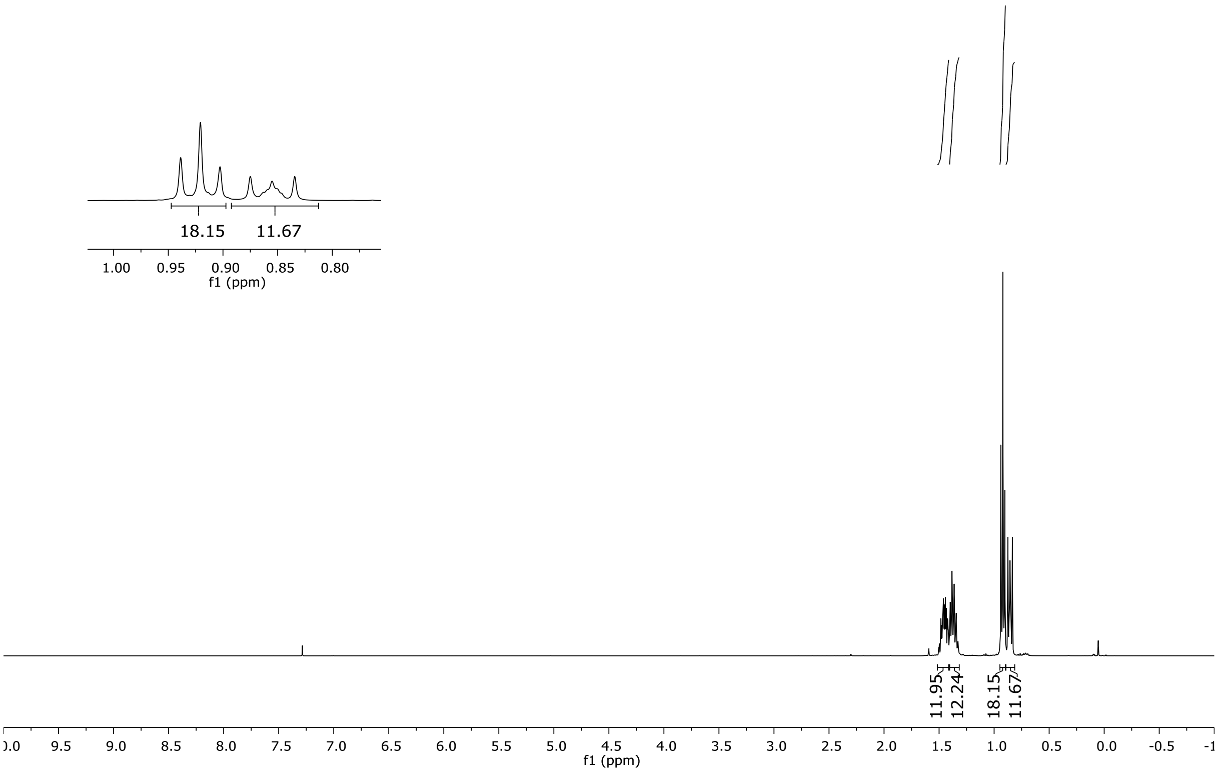

Figure S61. ^{1}H NMR Spectra of 1,2-bis(tributylgermyl)ethyne (**4b**)

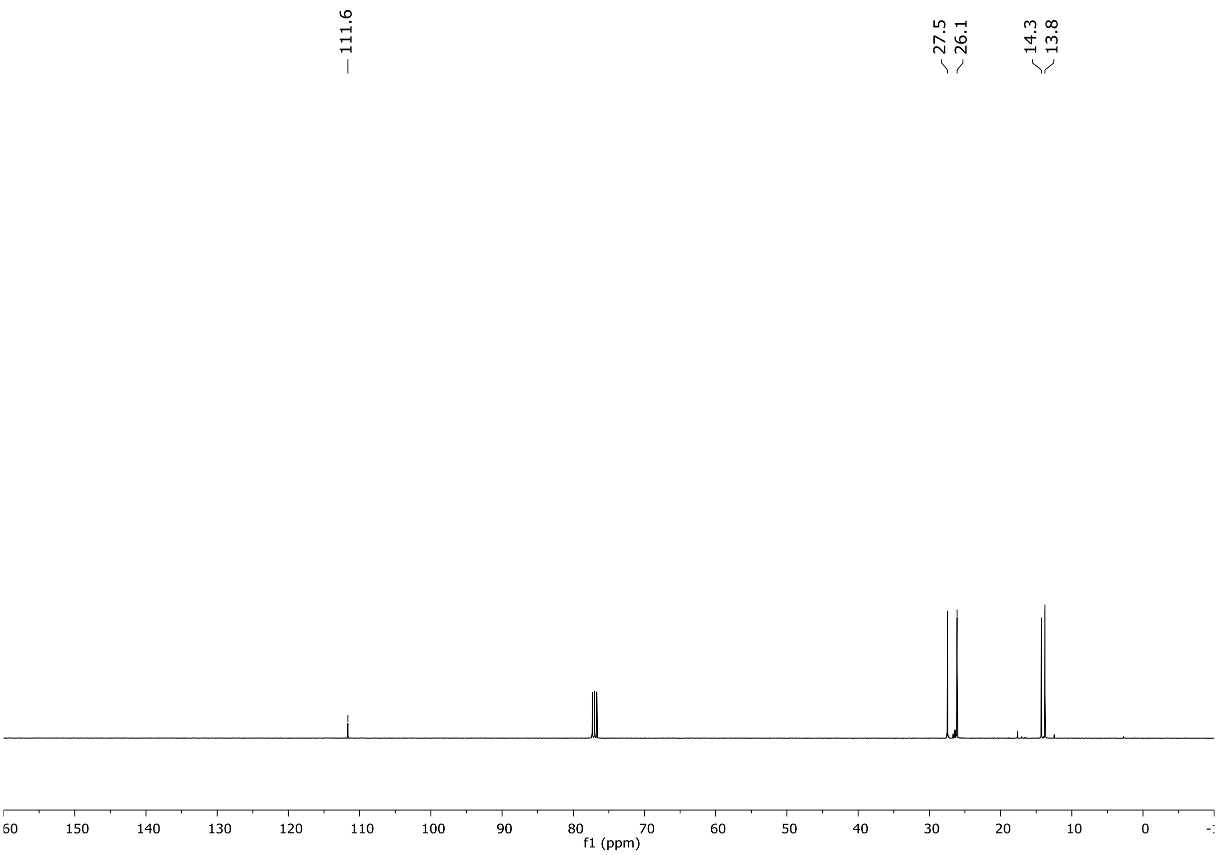

Figure S62. ^{13}C NMR Spectra of 1,2-bis(tributylgermyl)ethyne (**4b**)

1,2-Bis(triisopropylgermyl)ethyne (4c)

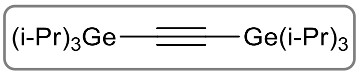

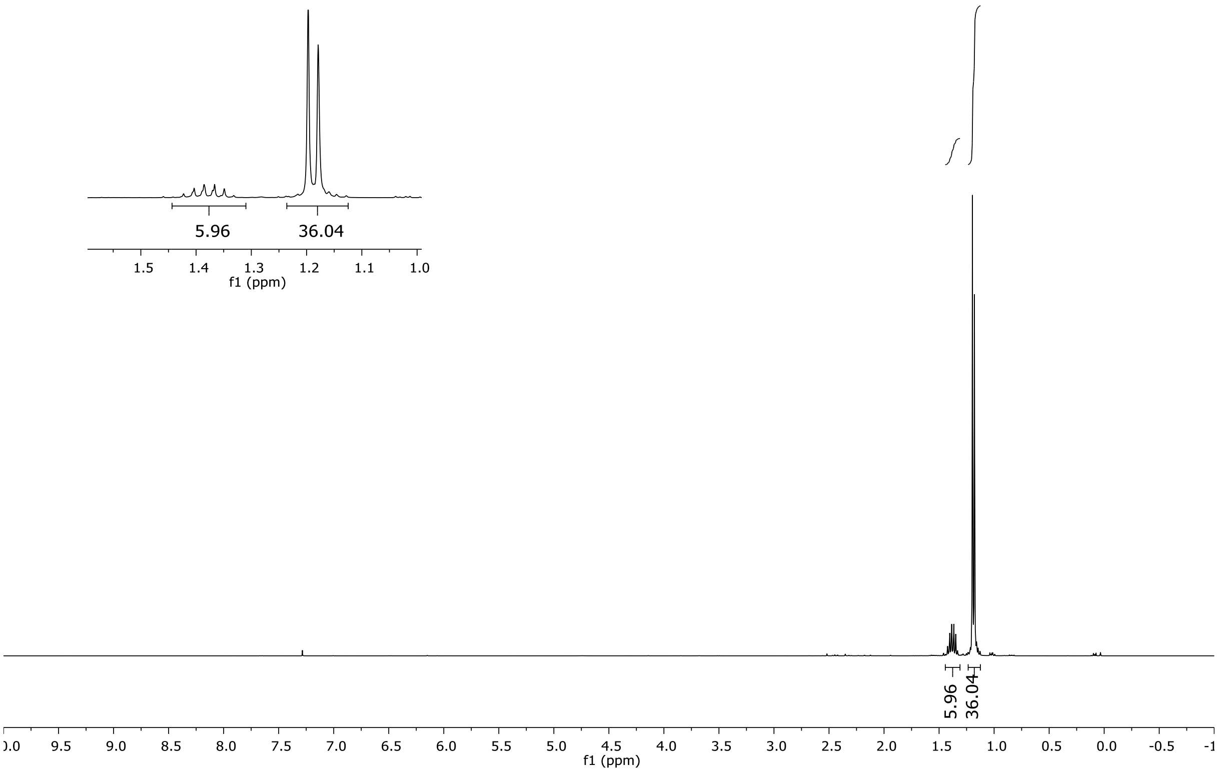

Figure S63. ^{1}H NMR Spectra of 1,2-bis(triisopropylgermyl)ethyne (**4c**)

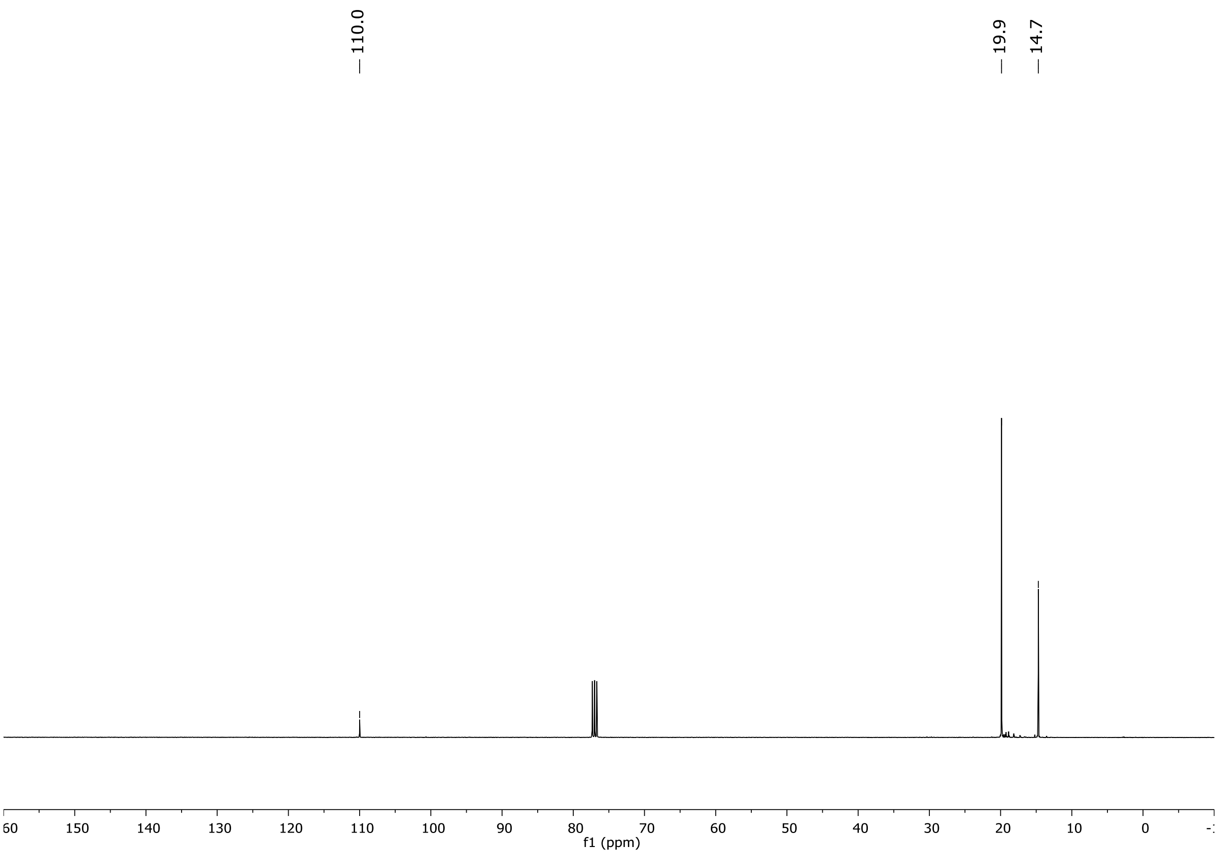

Figure S64. ^{13}C NMR Spectra of 1,2-bis(triisopropylgermyl)ethyne (**4c**)

1,2-Bis(dimethyl(phenyl)germyl)ethyne (4d)

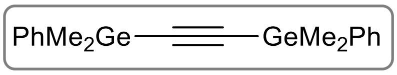

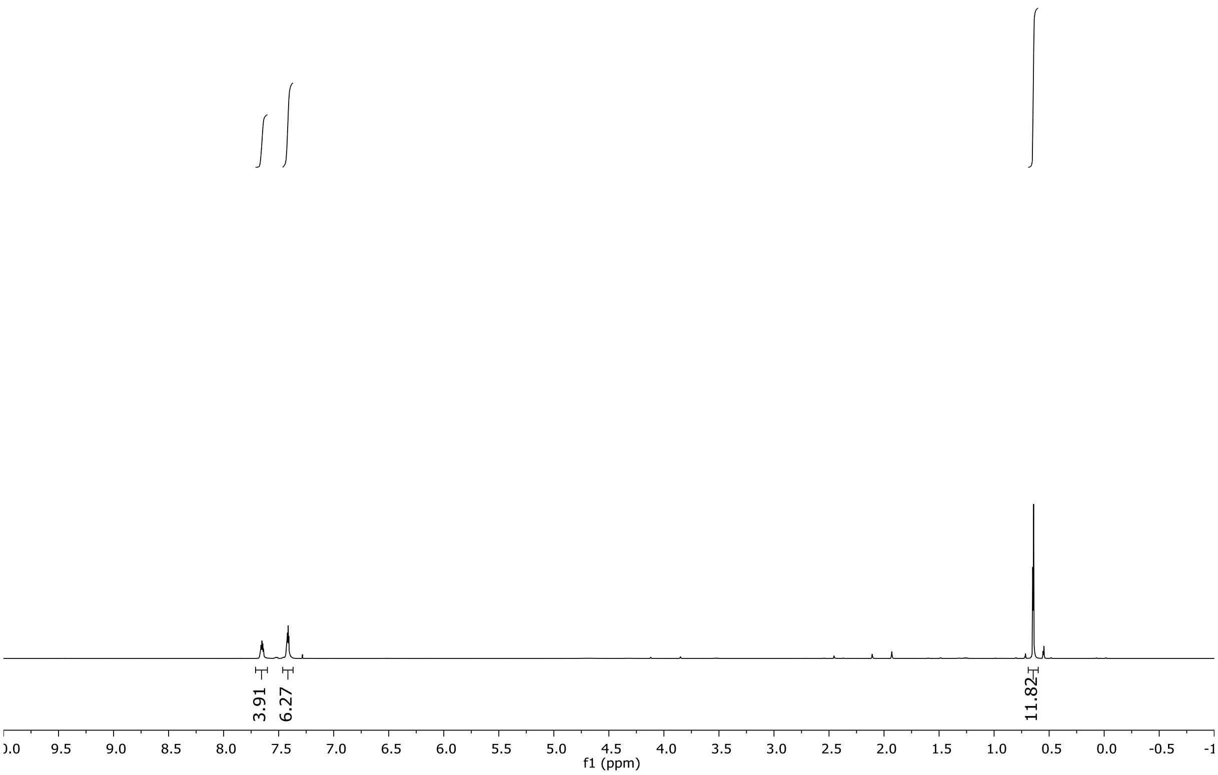

Figure S65. ^{1}H NMR Spectra of 1,2-bis(dimethyl(phenyl)germyl)ethyne (**4d**)

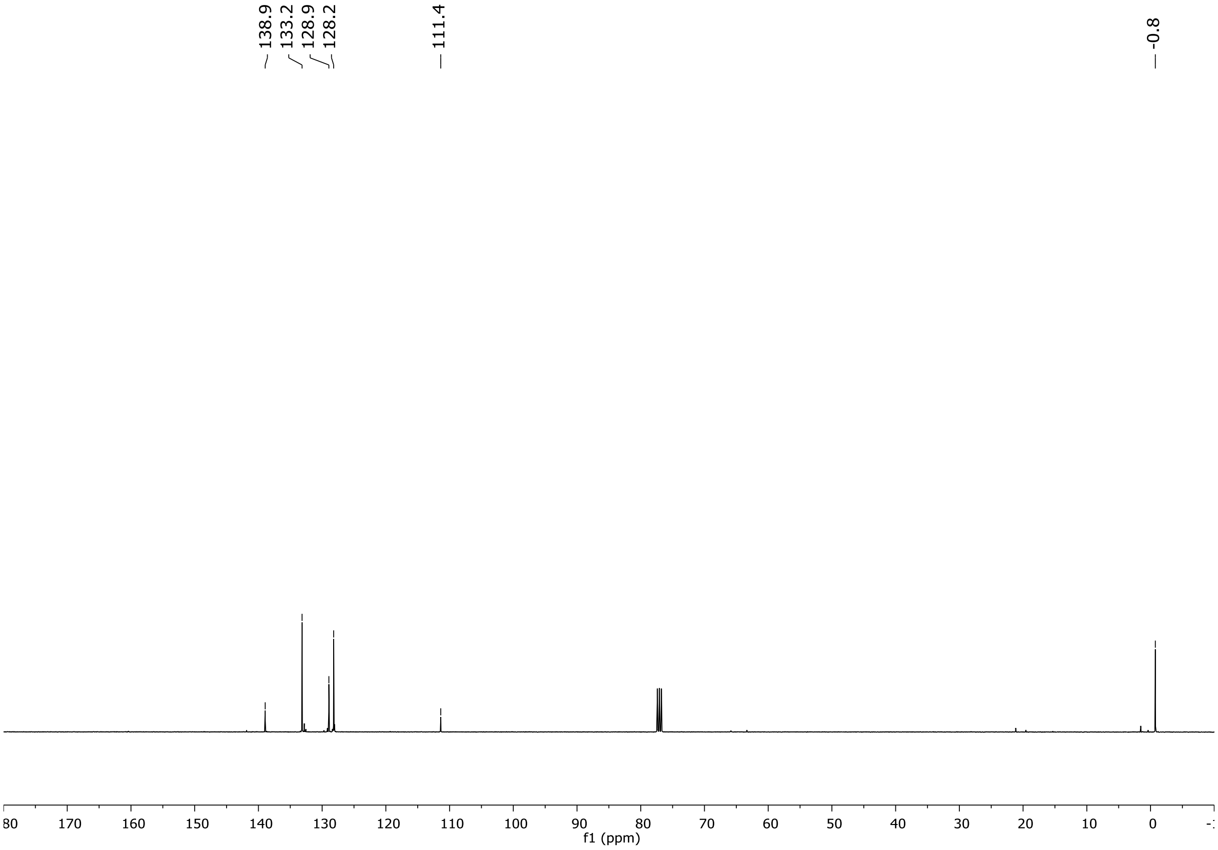

Figure S66. ^{13}C NMR Spectra of 1,2-bis(dimethyl(phenyl)germyl)ethyne (**4d**)

Triethyl(phenylethynyl)germane (6a)

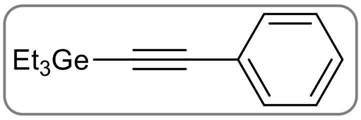

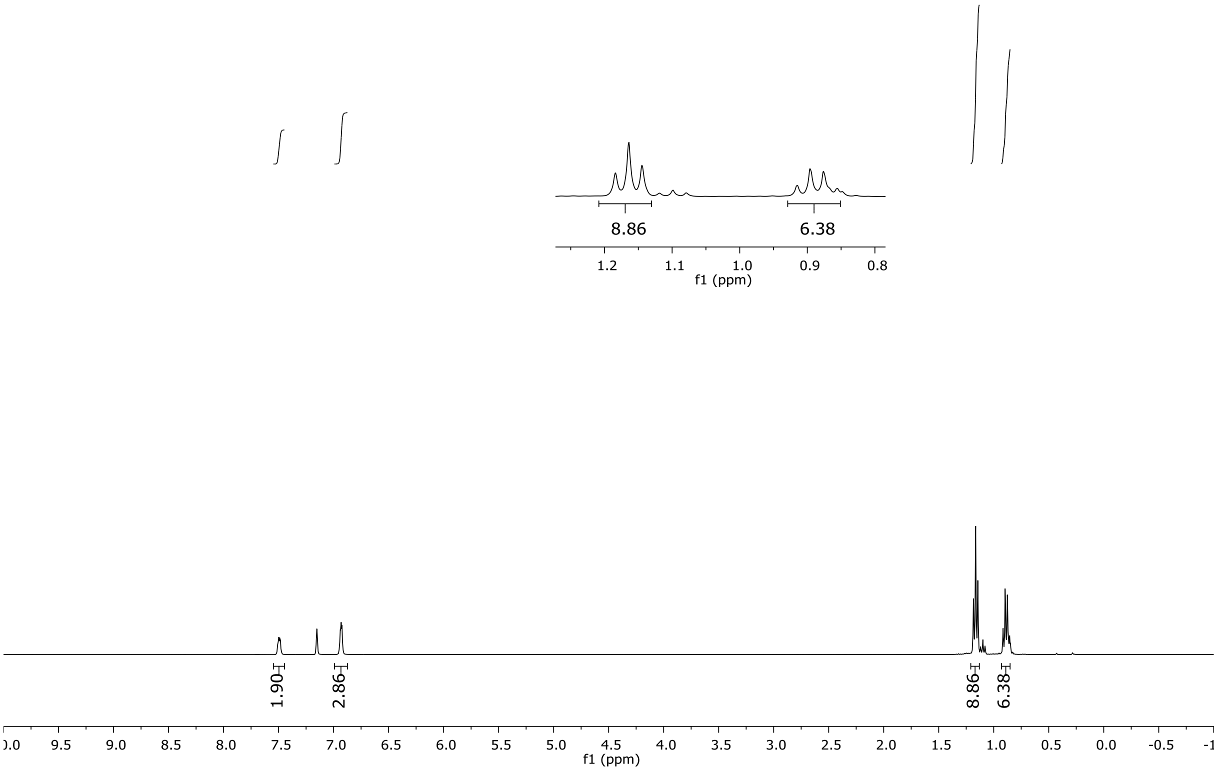

Figure S67. ^{1}H NMR Spectra of triethyl(phenylethynyl)germane (**6a**)

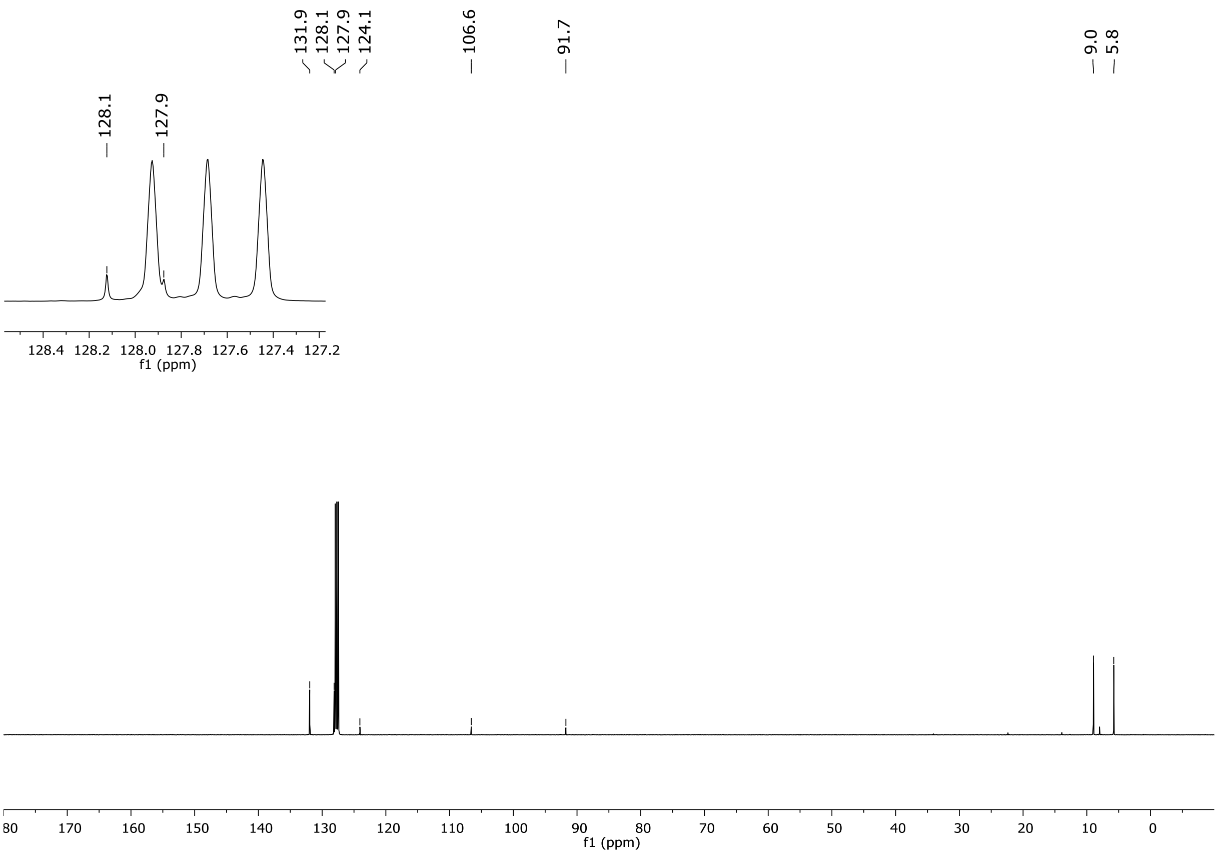

Figure S68. ^{13}C NMR Spectra of triethyl(phenylethynyl)germane (**6a**)

Triethyl((4-methoxyphenyl)ethynyl)germane (6b)

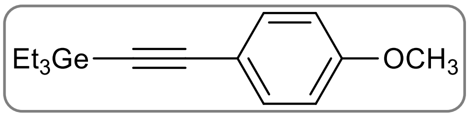

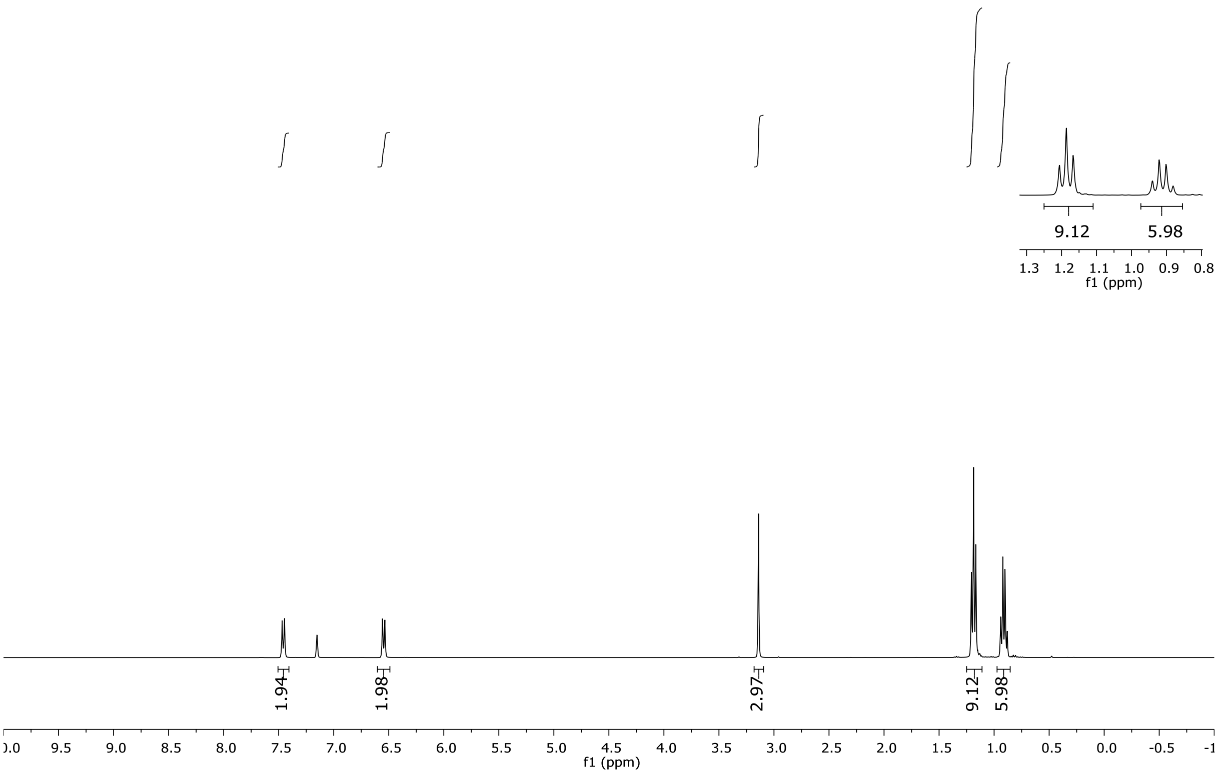

Figure S69. ^{1}H NMR Spectra of triethyl((4-methoxyphenyl)ethynyl)germane (**6b**)

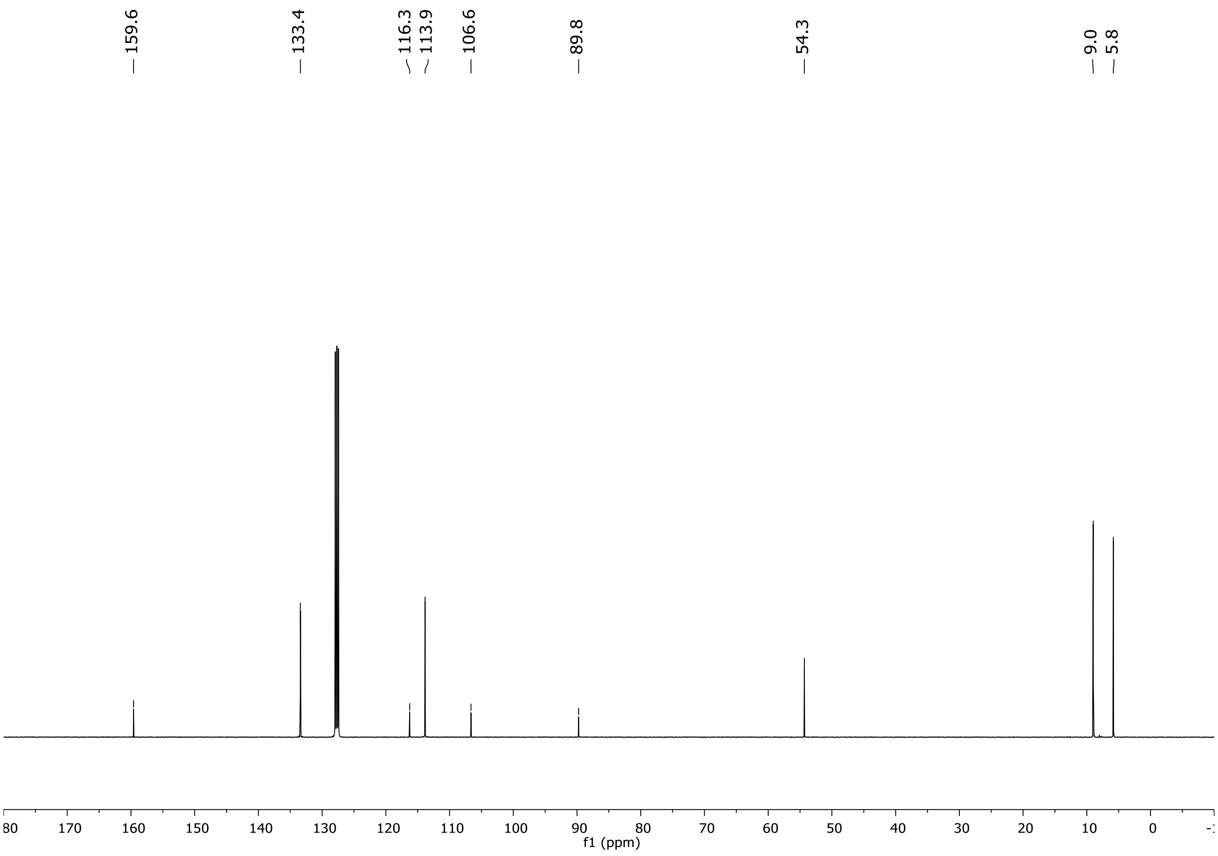

Figure S70. ^{13}C NMR Spectra of triethyl((4-methoxyphenyl)ethynyl)germane (**6b**)

Triethyl((4-(trifluoromethyl)phenyl)ethynyl)germane (6c)

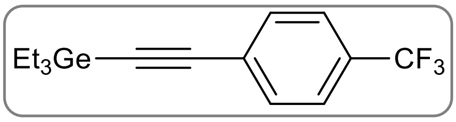

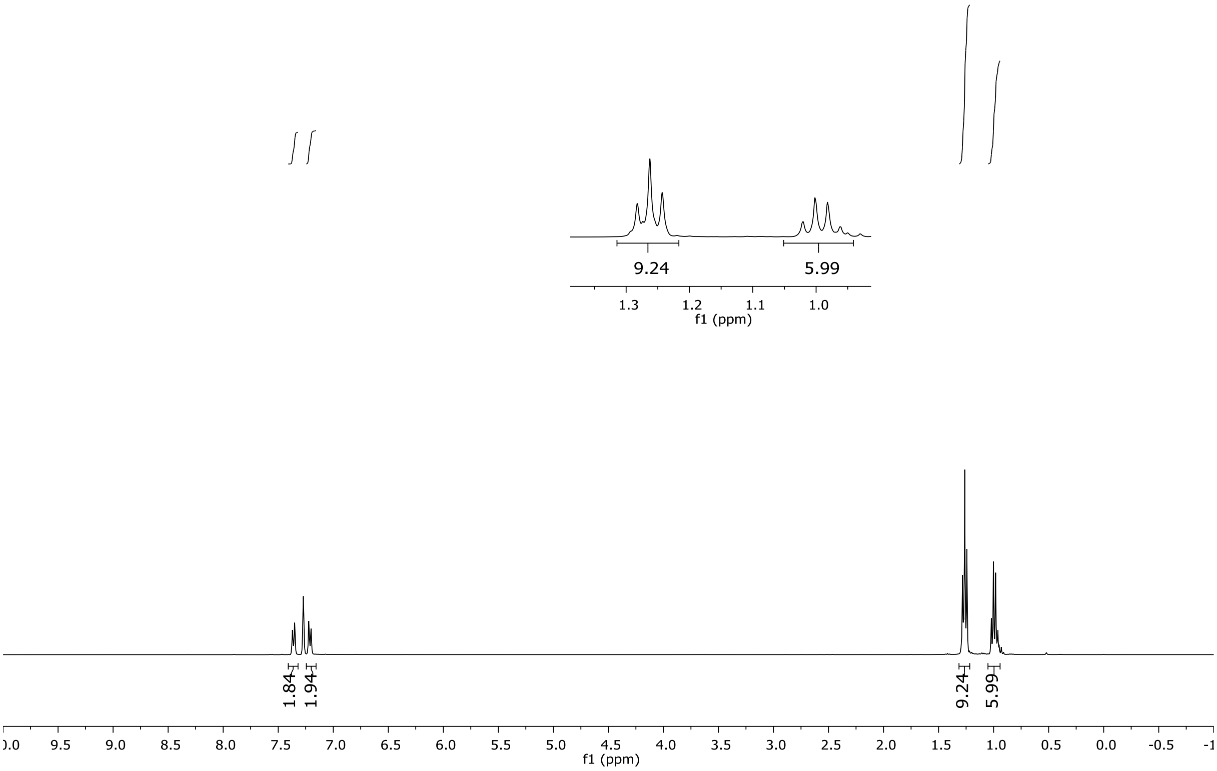

Figure S71. ^1H NMR Spectra of triethyl((4-(trifluoromethyl)phenyl)ethynyl)germane (**6c**)

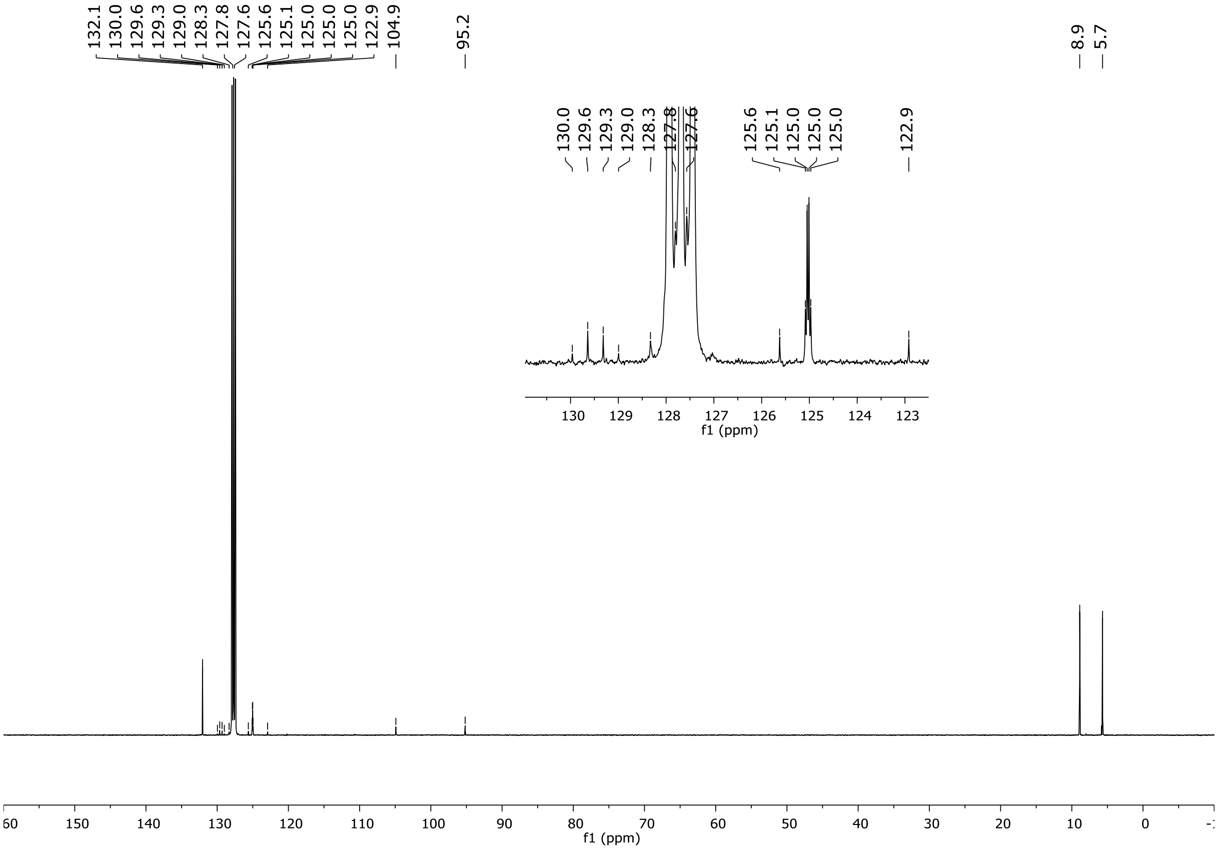

Figure S72. ^{13}C NMR Spectra of triethyl((4-(trifluoromethyl)phenyl)ethynyl)germane (**6c**)

(Cyclohex-1-en-1-ylethynyl)triethylgermane (6d)

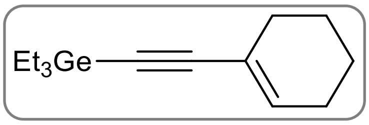

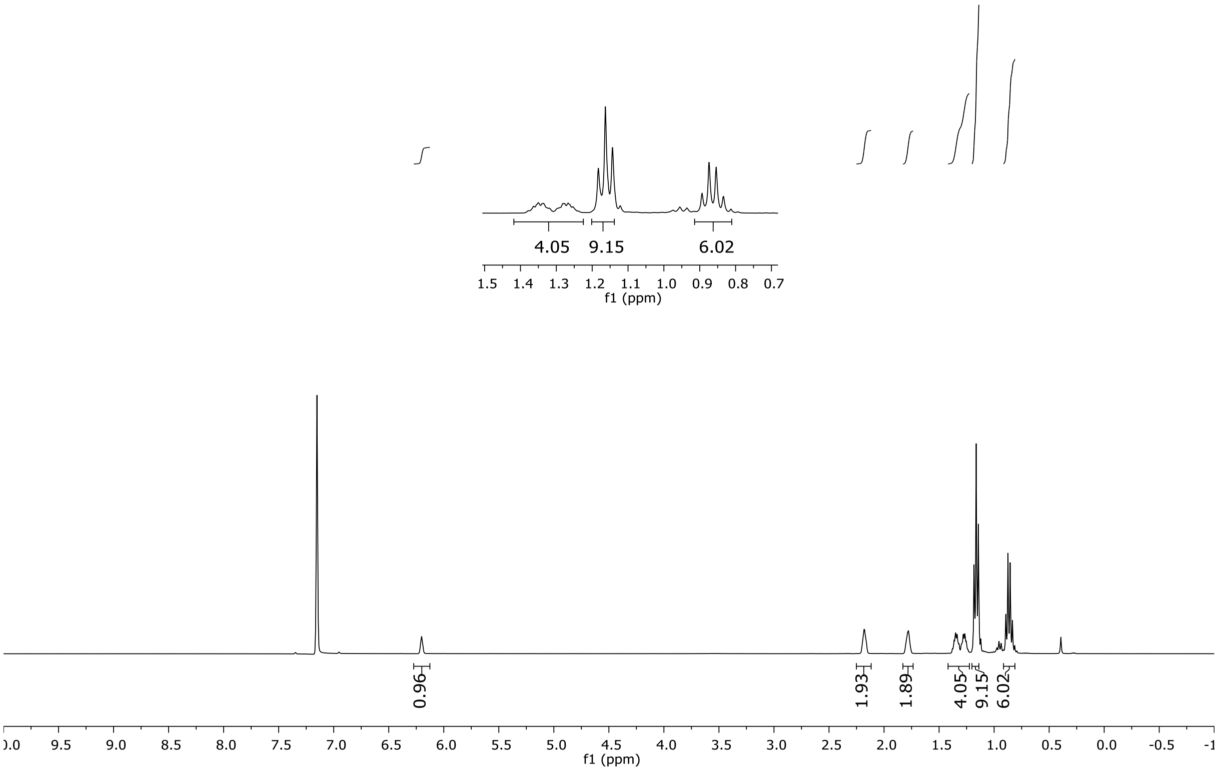

Figure S73. ^{1}H NMR Spectra of (cyclohex-1-en-1-ylethynyl)triethylgermane (**6d**)

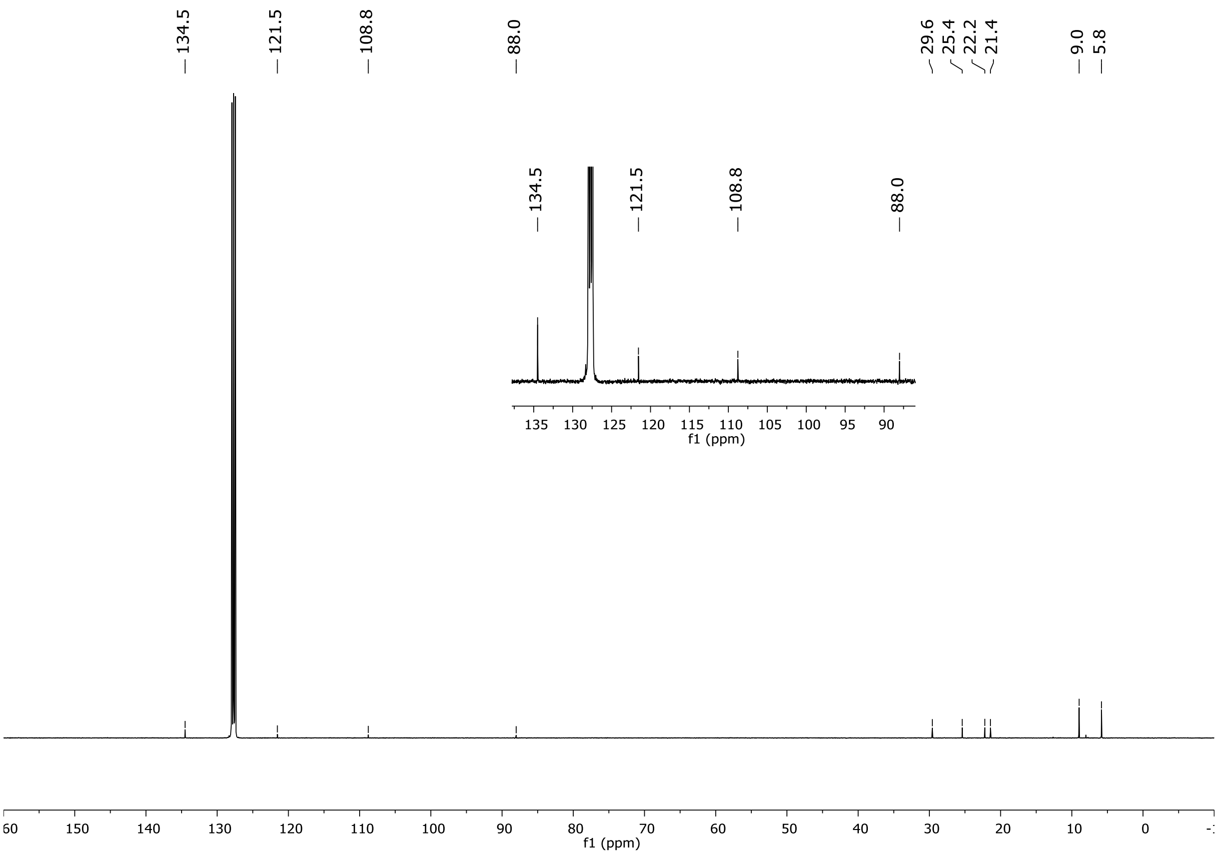

Figure S74. ^{13}C NMR Spectra of (cyclohex-1-en-1-ylethynyl)triethylgermane (**6d**)

Triethyl(naphthalen-1-ylethynyl)germane (6e)

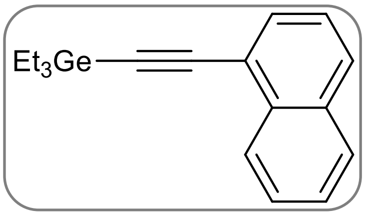

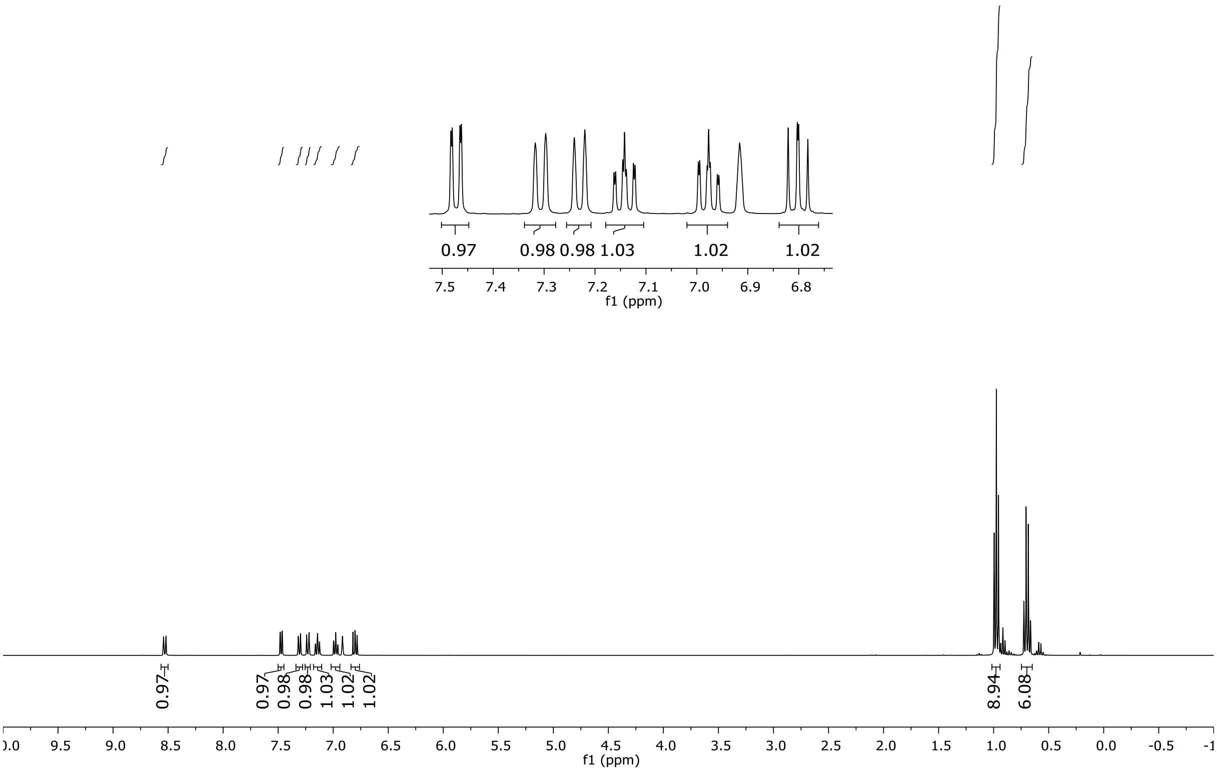

Figure S75. ^{1}H NMR Spectra of triethyl(naphthalen-1-ylethynyl)germane (**6e**)

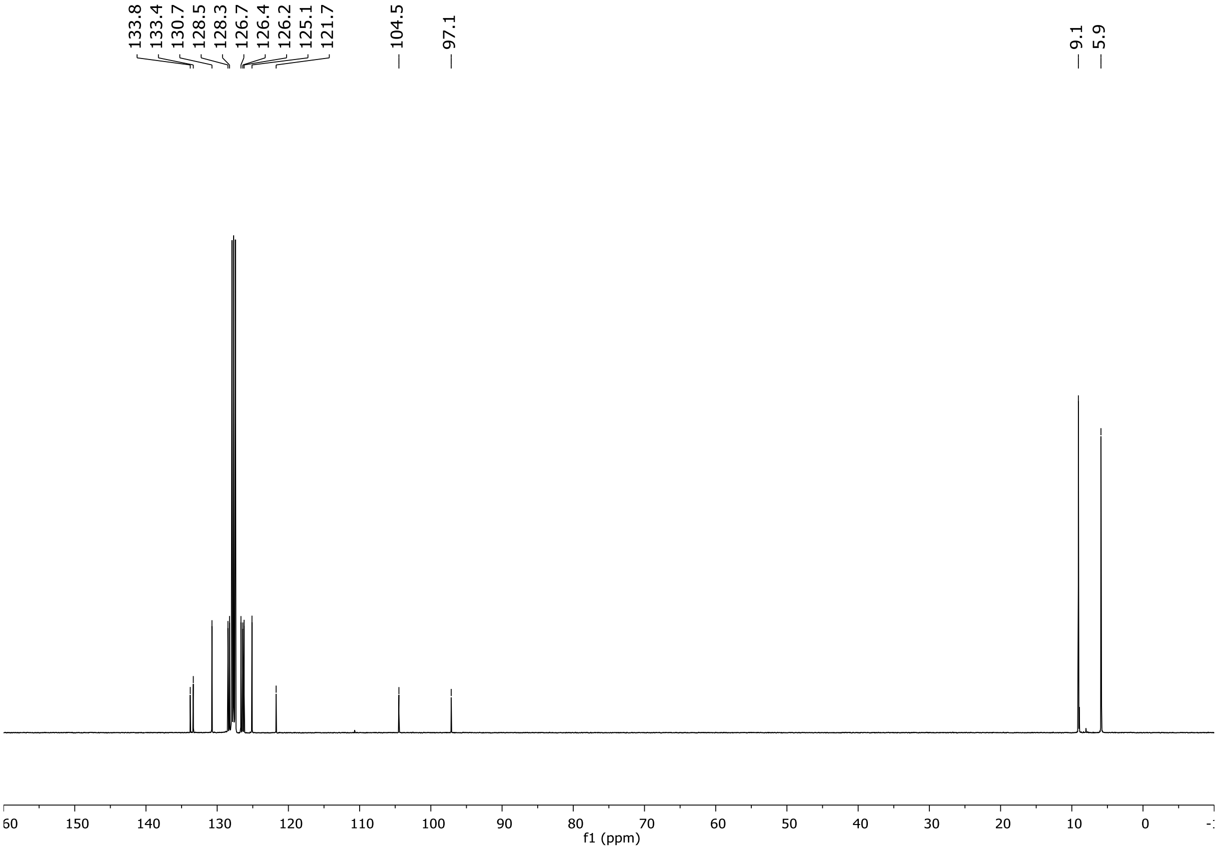

Figure S76. ^{13}C NMR Spectra of triethyl(naphthalen-1-ylethynyl)germane (**6e**)

N-Benzyl-N-methyl-3-(triethylgermyl)prop-2-yn-1-amine (6f)

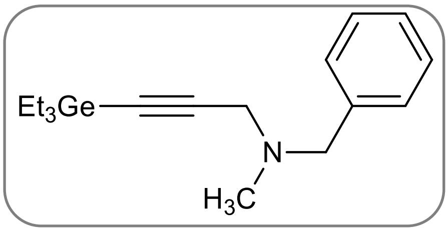

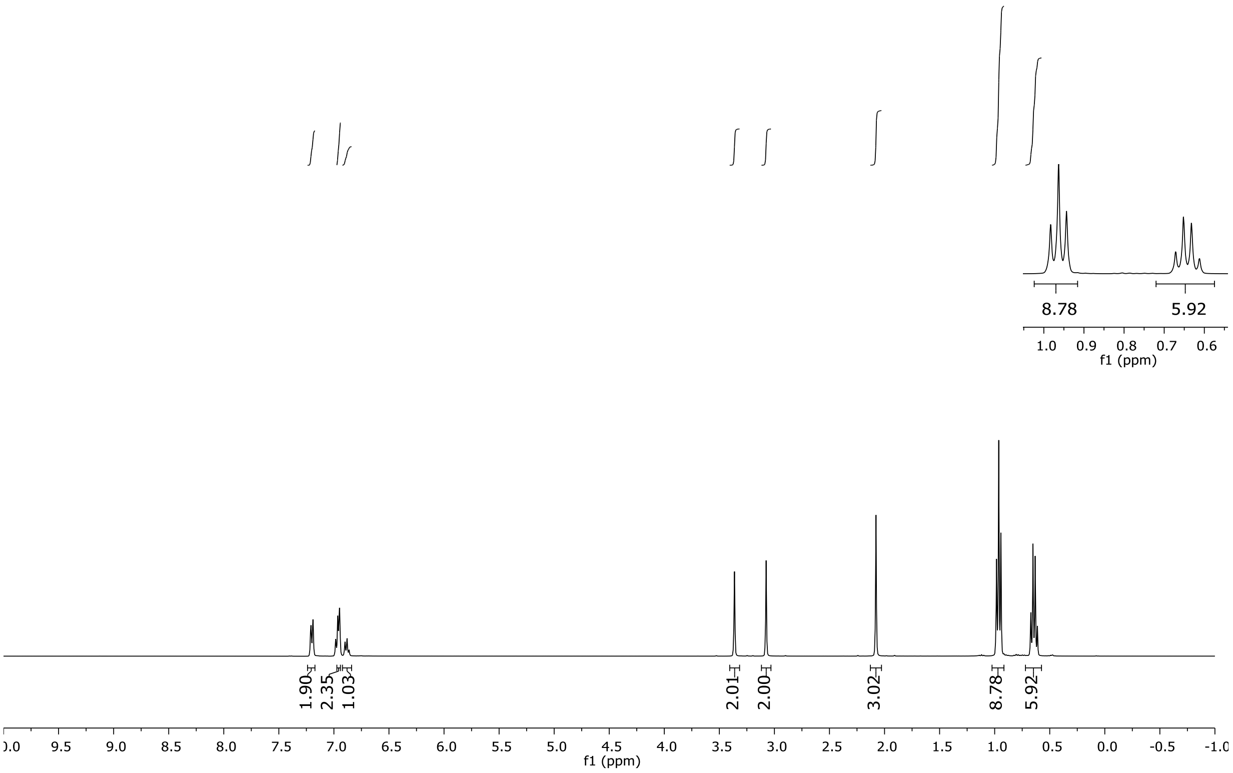

Figure S77. ^{1}H NMR Spectra of N-benzyl-N-methyl-3-(triethylgermyl)prop-2-yn-1-amine (**6f**)

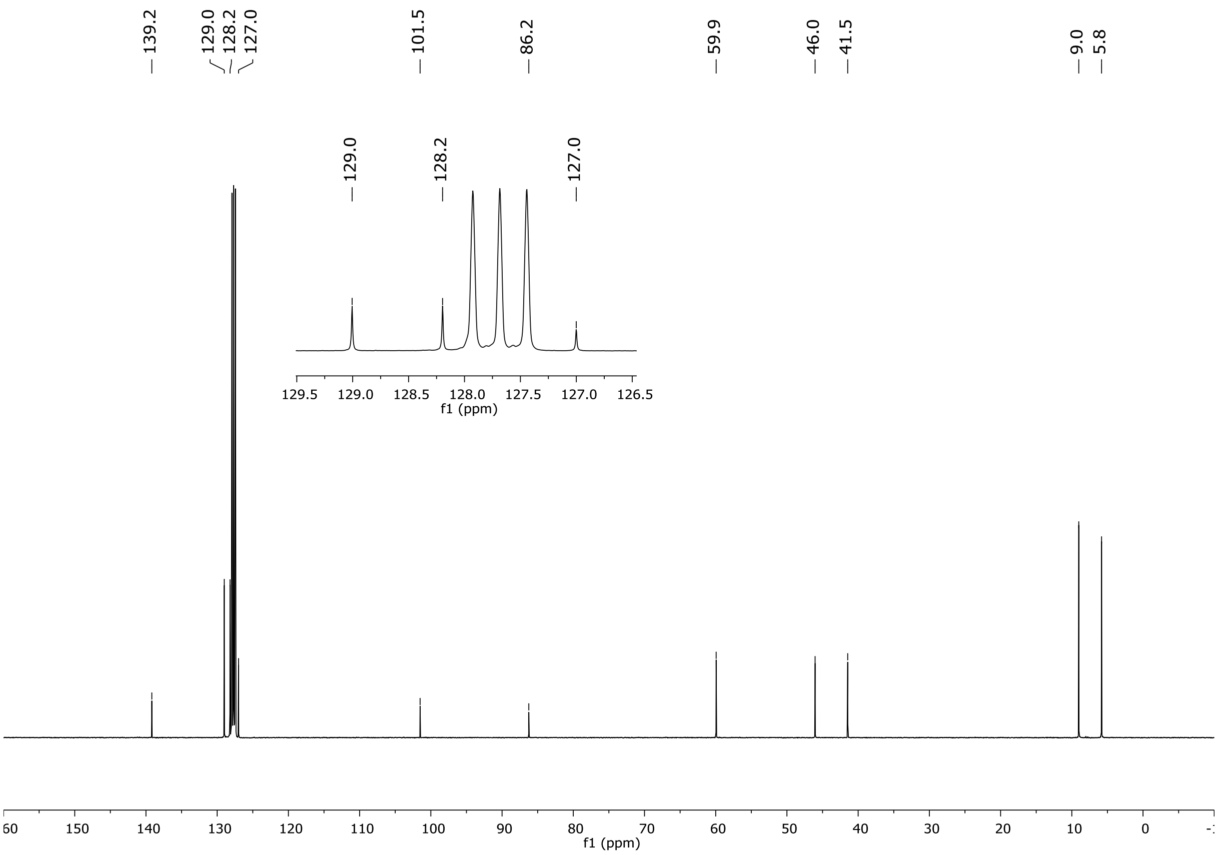

Figure S78. ^{13}C NMR Spectra of N-benzyl-N-methyl-3-(triethylgermyl)prop-2-yn-1-amine (**6f**)

3-((Triethylgermyl)ethynyl)aniline (6g)

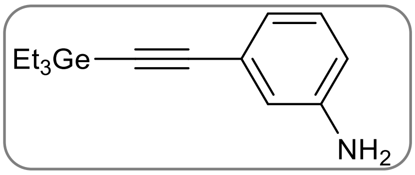

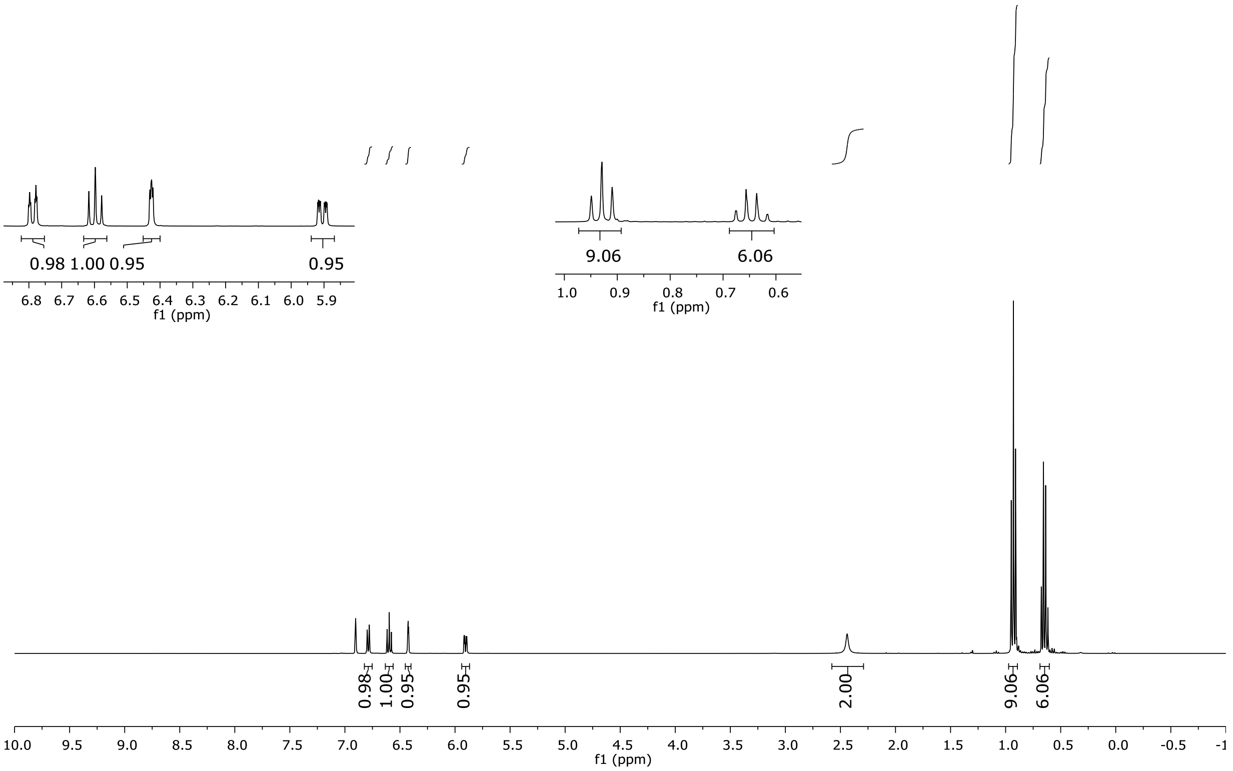

Figure S79. ^{1}H NMR Spectra of 3-((triethylgermyl)ethynyl)aniline (**6g**)

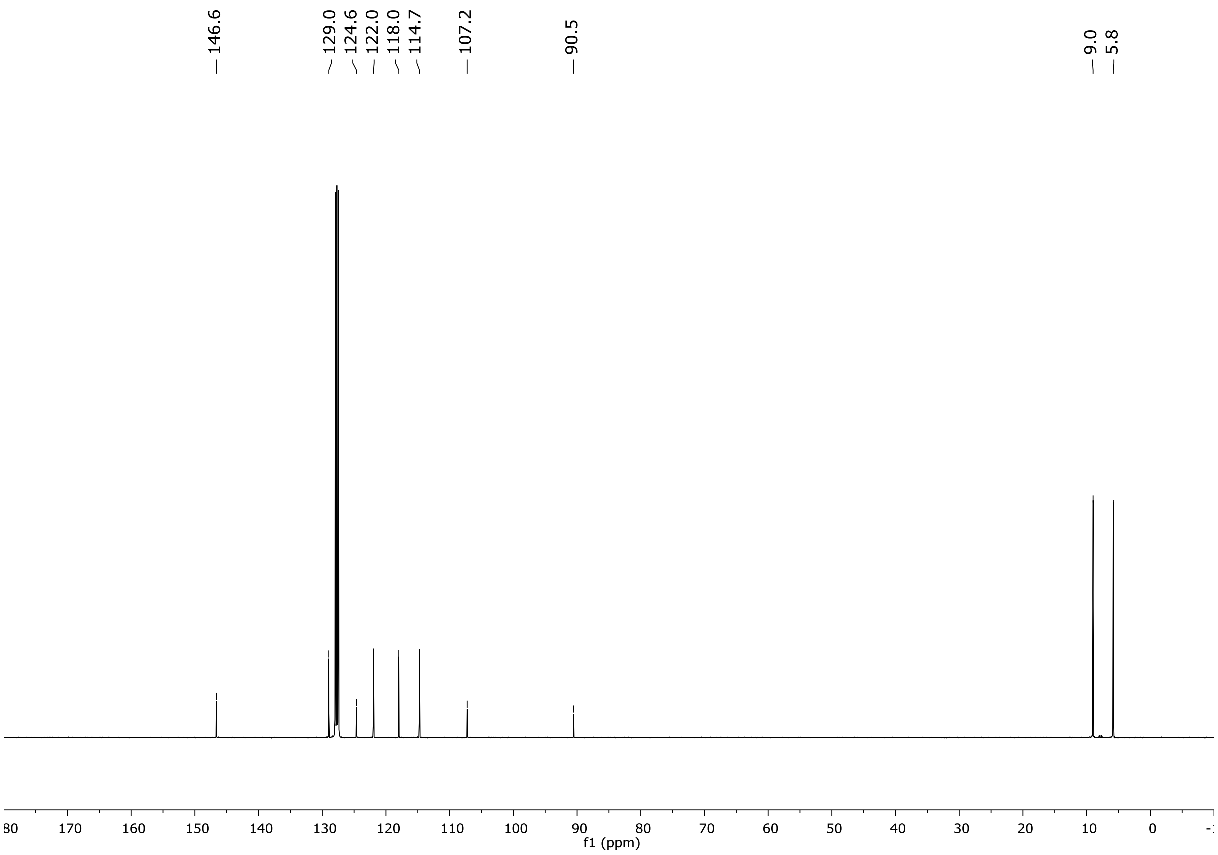

Figure S80. ^{13}C NMR Spectra of 3-((triethylgermyl)ethynyl)aniline (**6g**)

2-((Triethylgermyl)ethynyl)pyridine (6h)

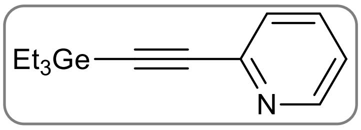

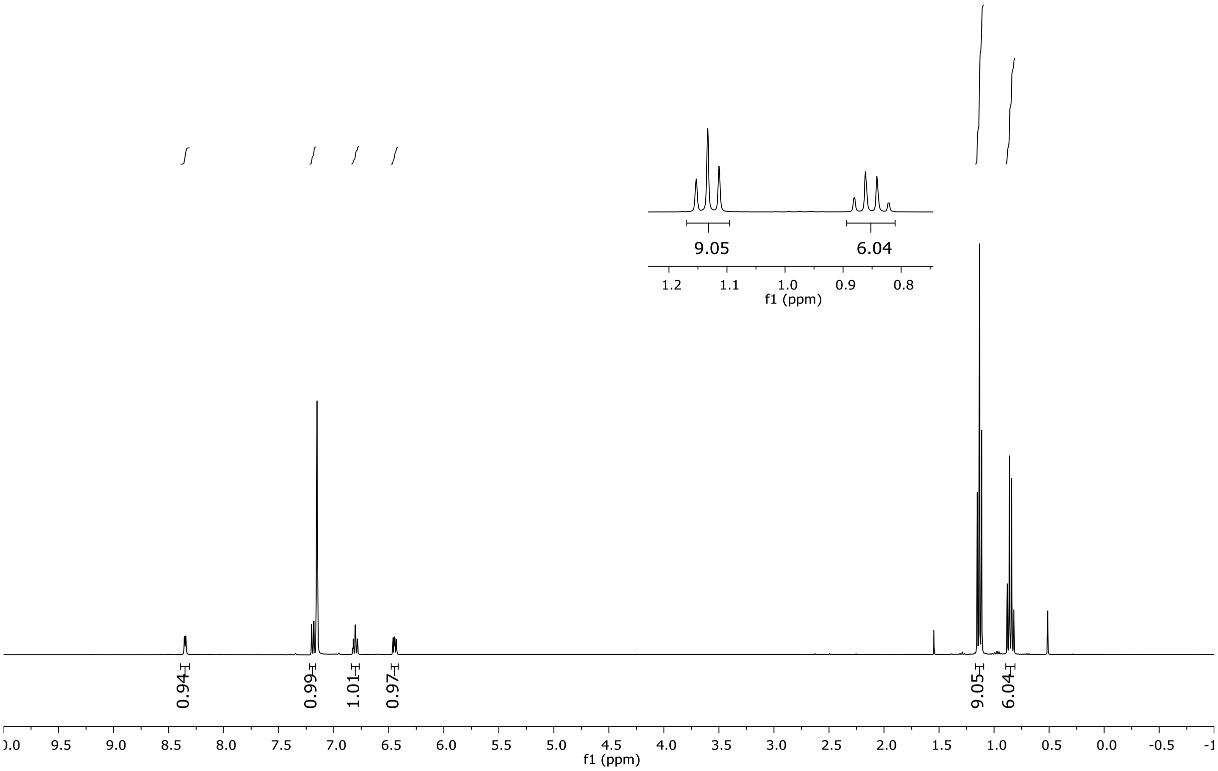

Figure S81. ^{1}H NMR Spectra of 2-((triethylgermyl)ethynyl)pyridine (**6h**)

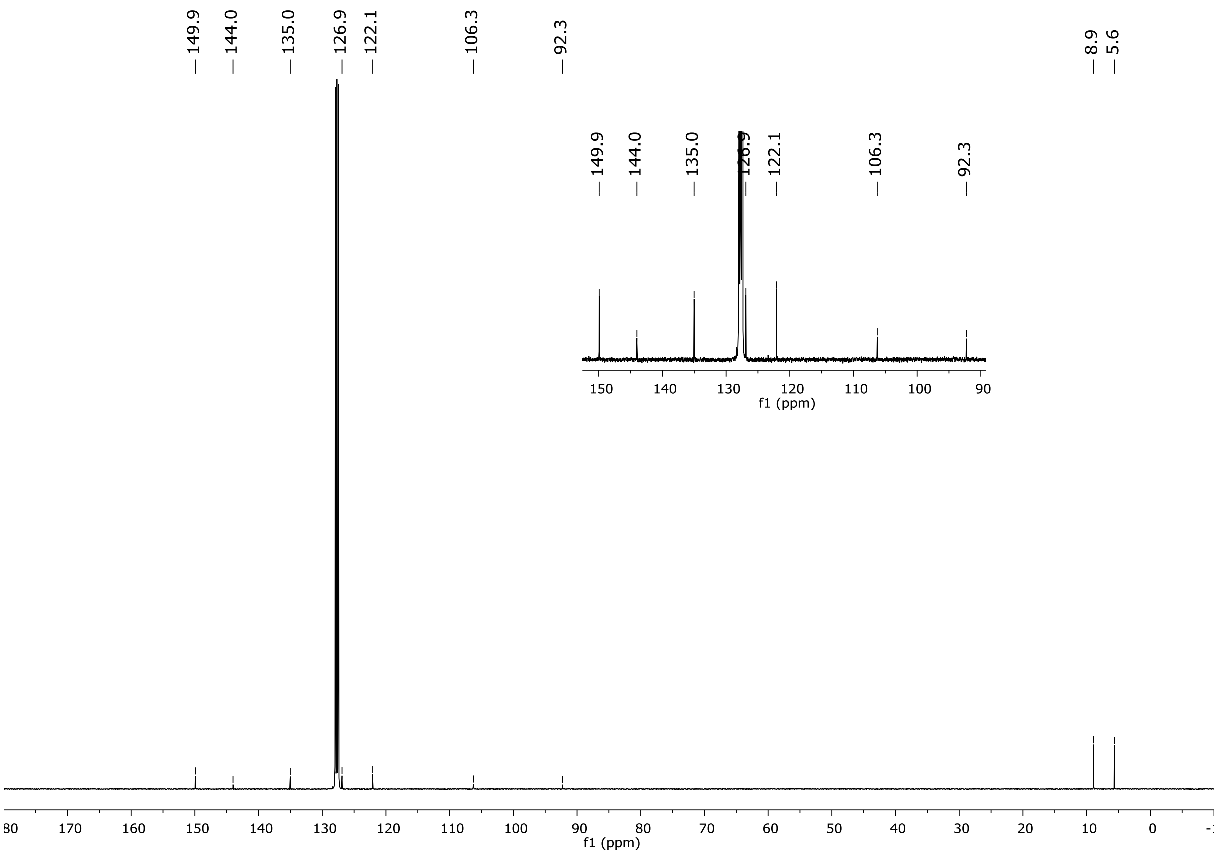

Figure S82. ^{13}C NMR Spectra of 2-((triethylgermyl)ethynyl)pyridine (**6h**)

1,3-Bis((triethylgermyl)ethynyl)benzene (6i)

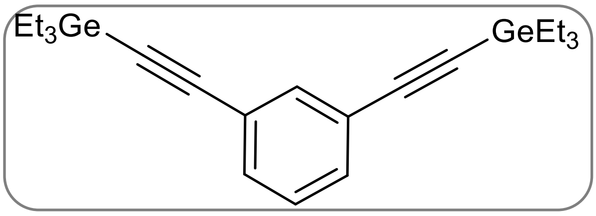

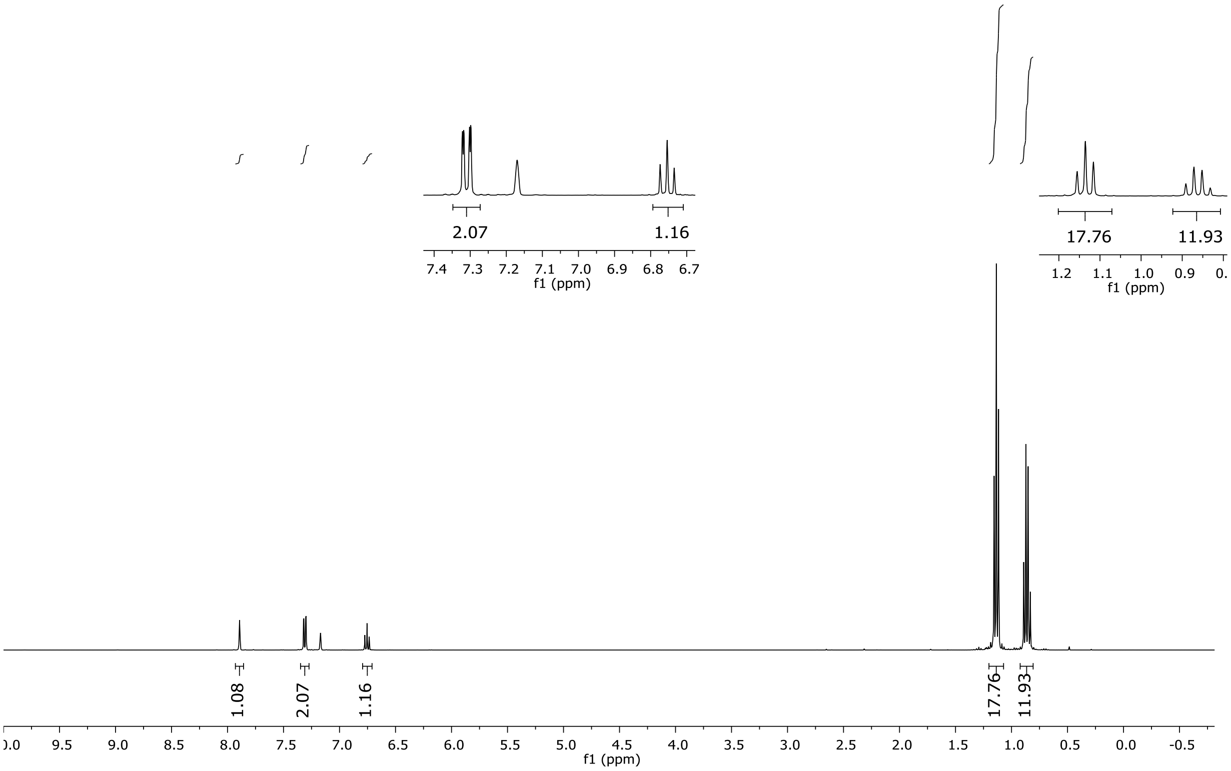

Figure S83. ^{1}H NMR Spectra of 1,3-bis((triethylgermyl)ethynyl)benzene (**6i**)

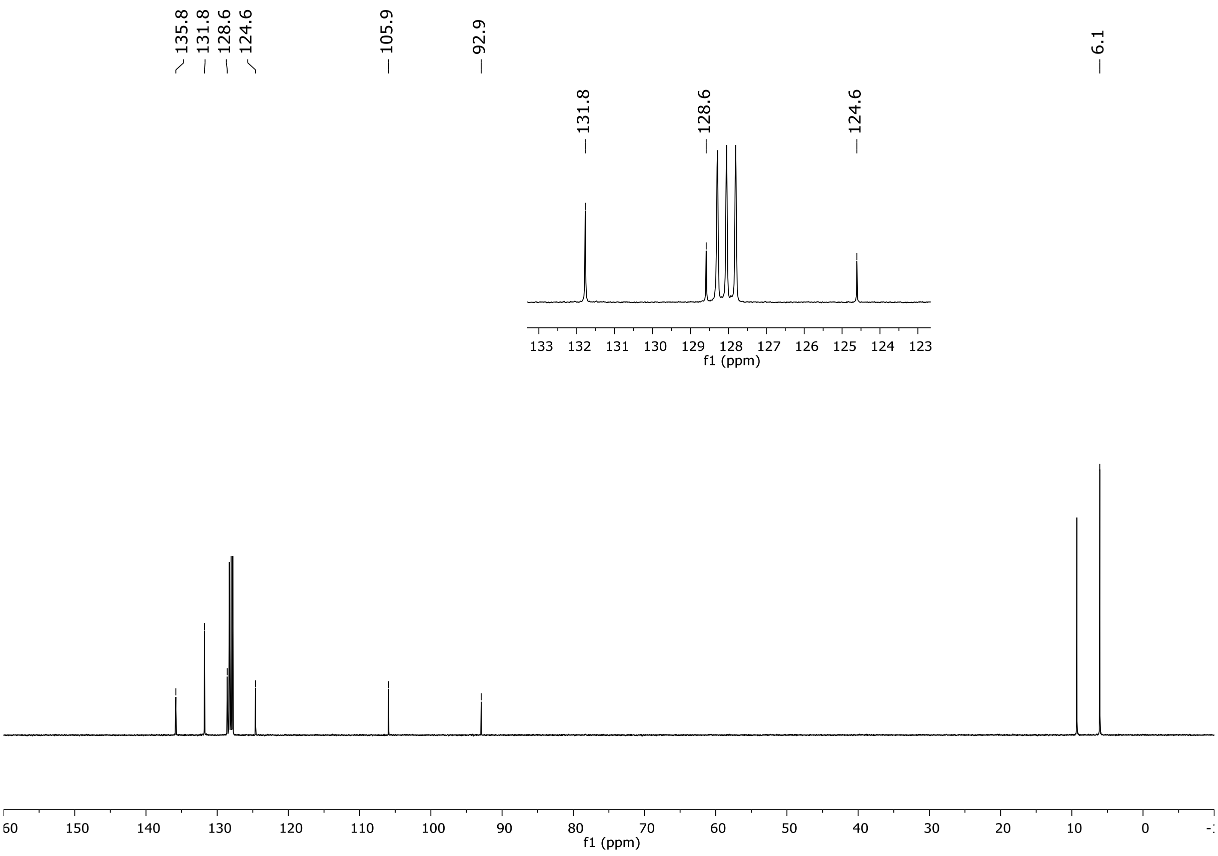

Figure S84. ^{13}C NMR Spectra of 1,3-bis((triethylgermyl)ethynyl)benzene (**6i**)

Triisopropyl((triethylgermyl)ethynyl)silane (6j)

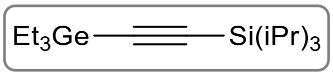

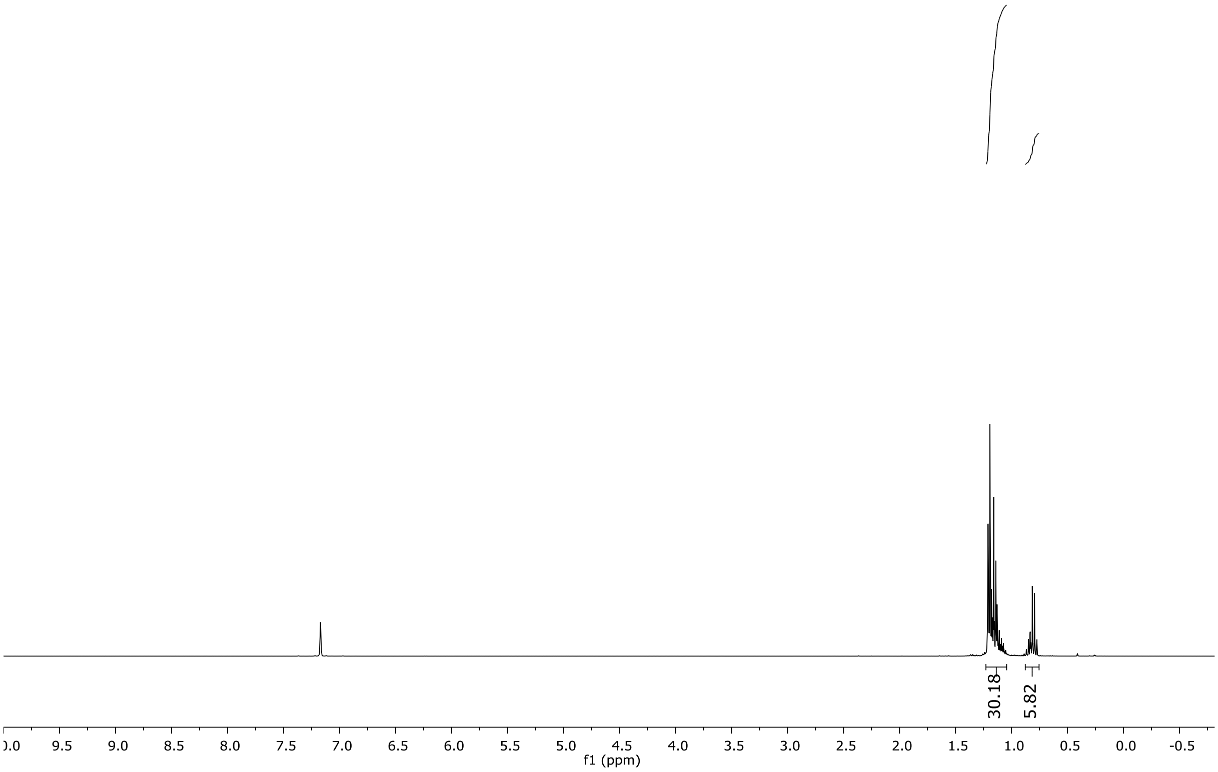

Figure S85. ^{1}H NMR Spectra of triisopropyl((triethylgermyl)ethynyl)silane (**6j**)

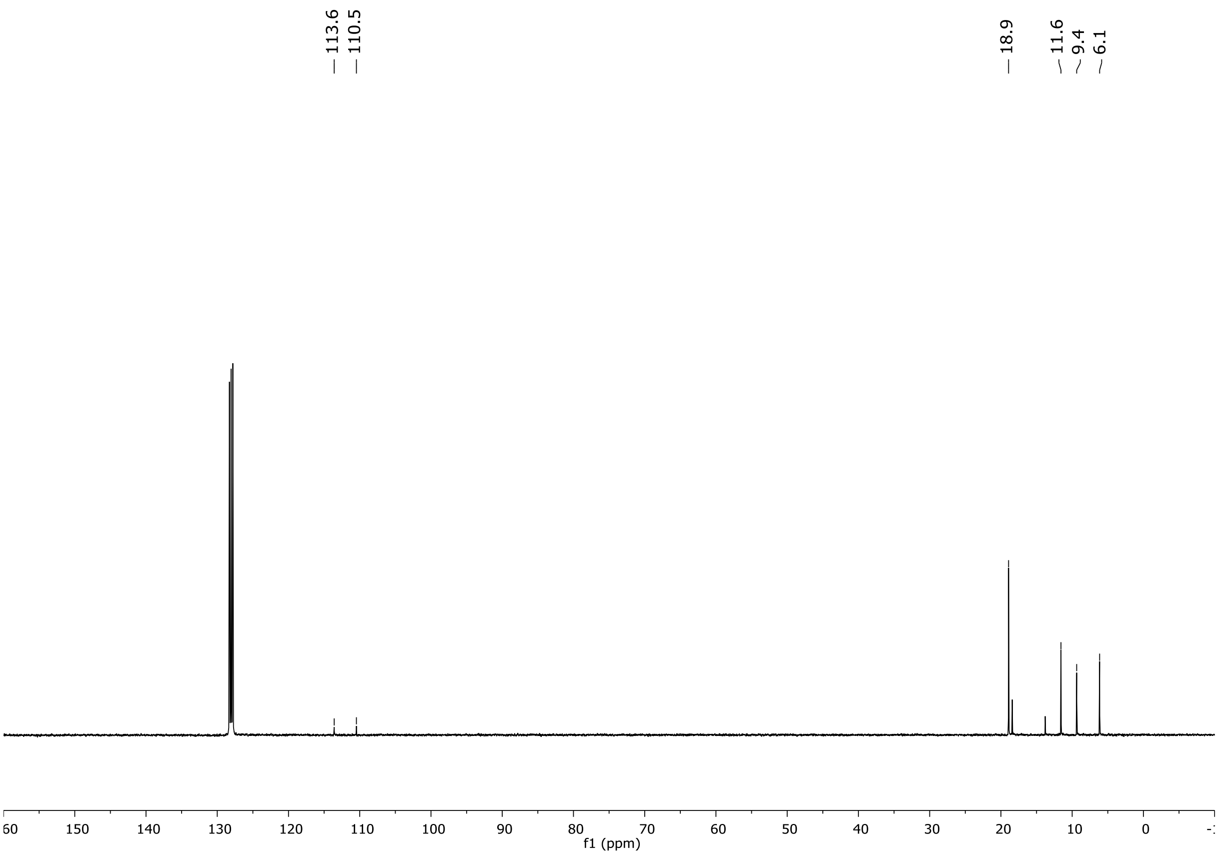

Figure S86. ^{13}C NMR Spectra of triisopropyl((triethylgermyl)ethynyl)silane (**6j**)

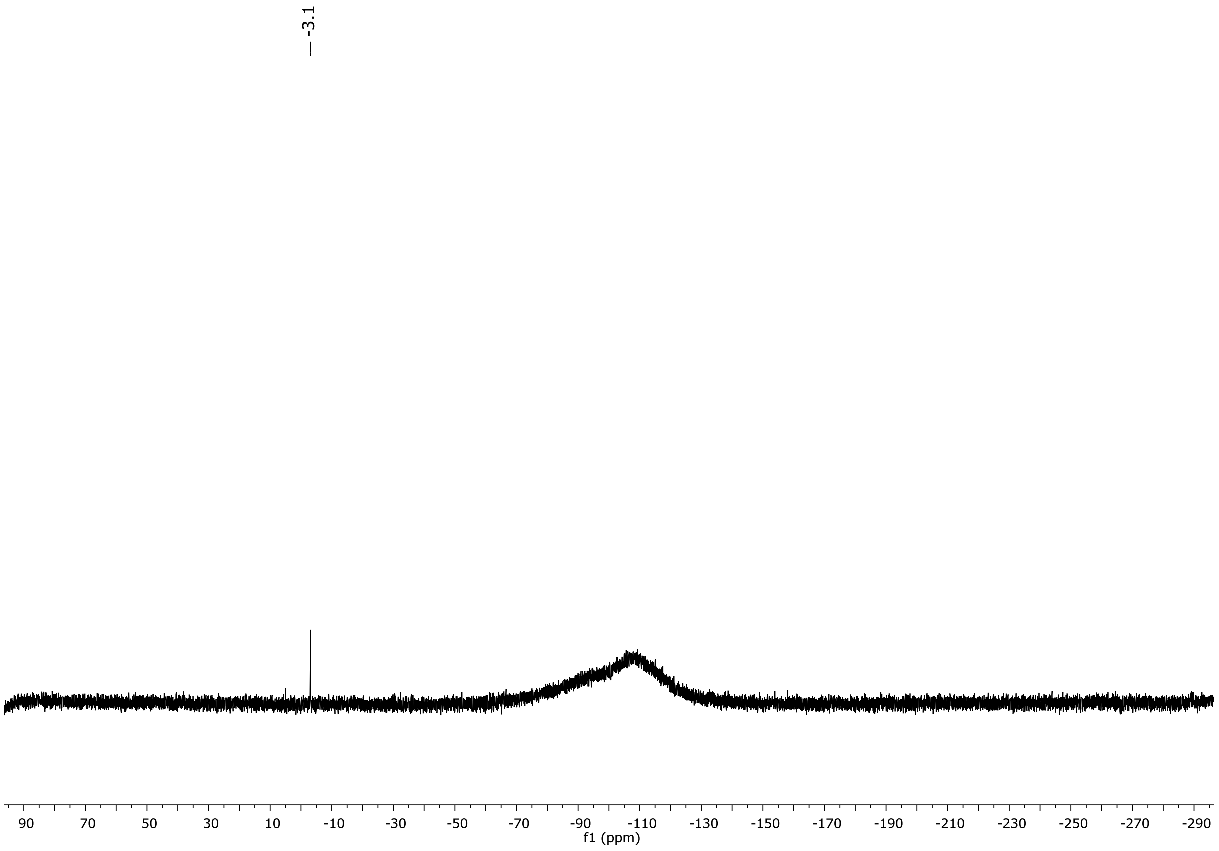

Figure S87. ^{29}Si NMR Spectra of triisopropyl((triethylgermyl)ethynyl)silane (**6j**)

Triethyl((triisopropylgermyl)ethynyl)germane (6k)

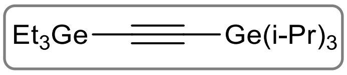

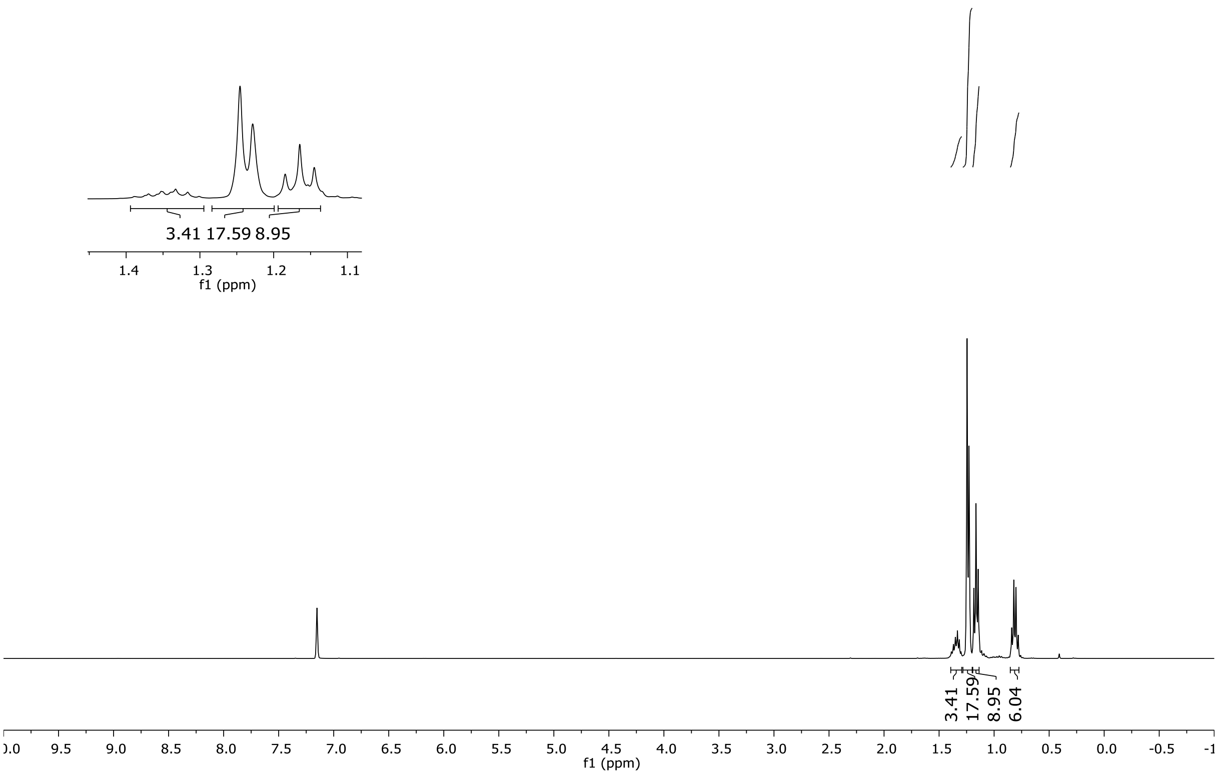

Figure S88. ^{1}H NMR Spectra of triethyl((triisopropylgermyl)ethynyl)germane (**6k**)

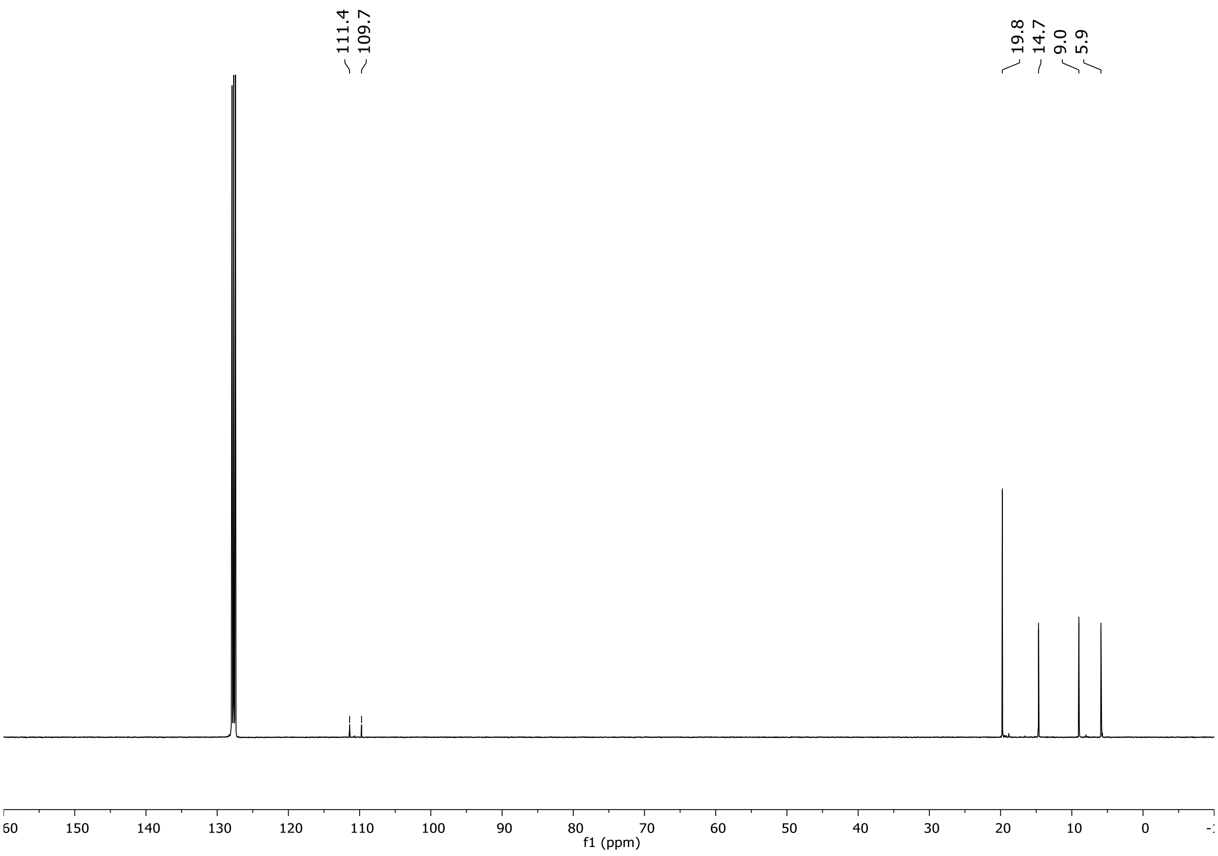

Figure S89. ^{13}C NMR Spectra of triethyl((triisopropylgermyl)ethynyl)germane (**6k**)

Tert-butyldimethyl((triethylgermyl)ethynyl)silane (6l)

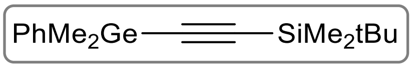

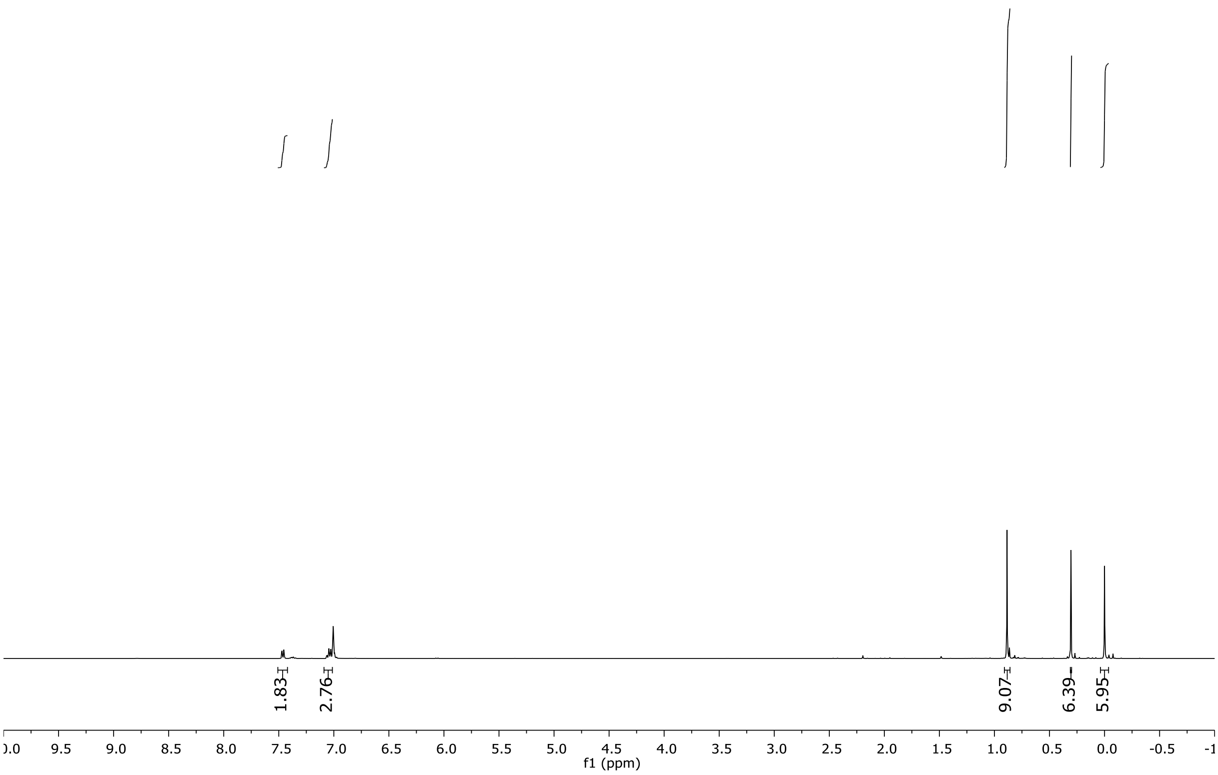

Figure S90. ^{1}H NMR Spectra of tert-butyldimethyl((triethylgermyl)ethynyl)silane (**6l**)

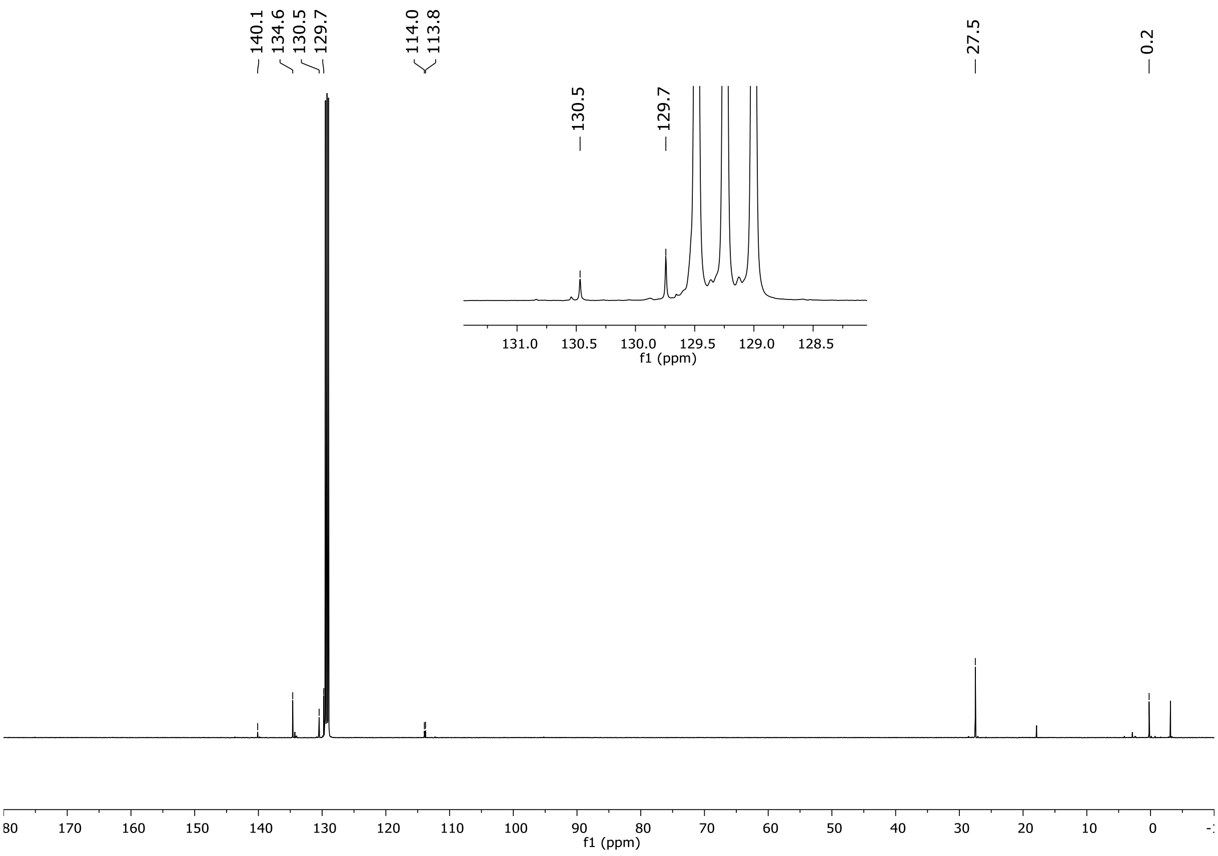

Figure S91. ^{13}C NMR Spectra of tert-butyldimethyl((triethylgermyl)ethynyl)silane (**6l**)

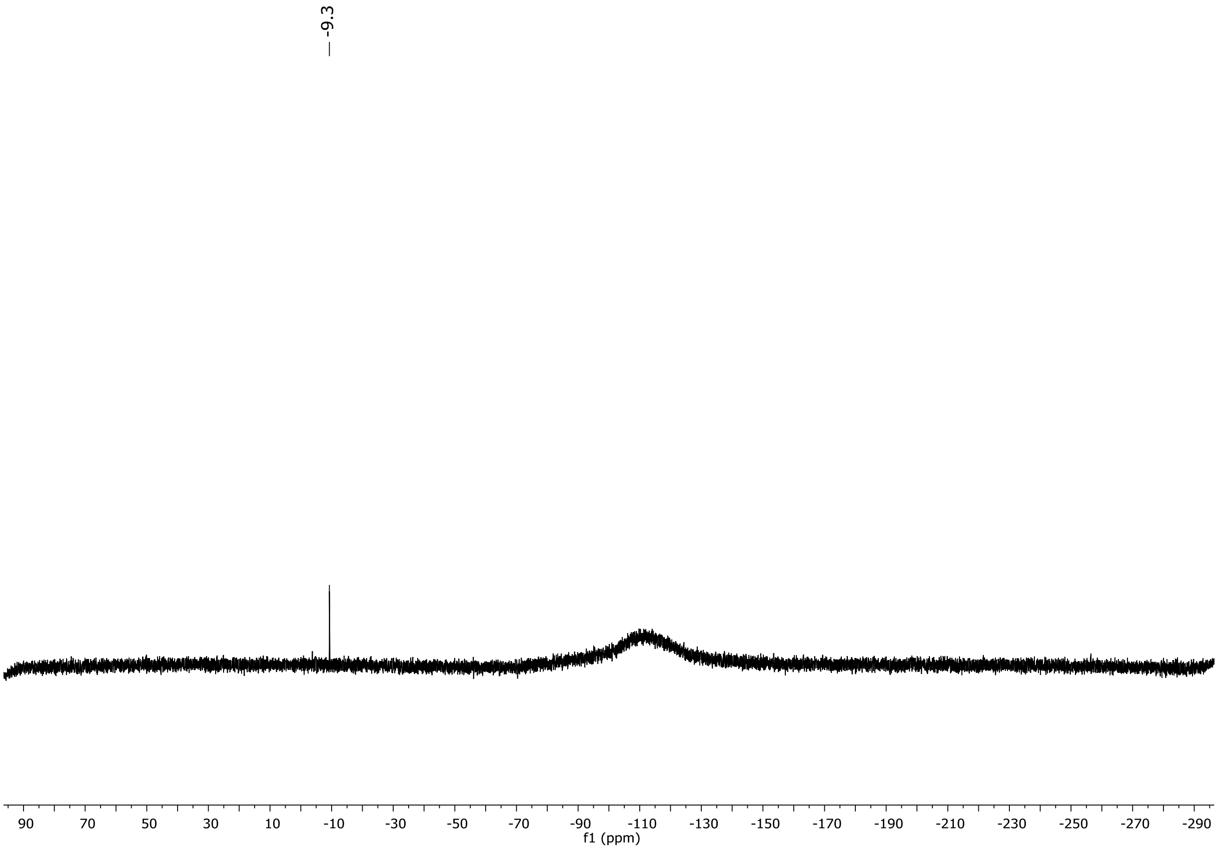

Figure S92. ^{29}Si NMR Spectra of tert-butyldimethyl((triethylgermyl)ethynyl)silane (**6l**)

Hex-1-yn-1-yldimethyl(phenyl)germane (6m)

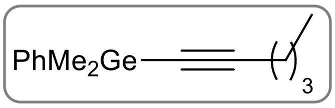

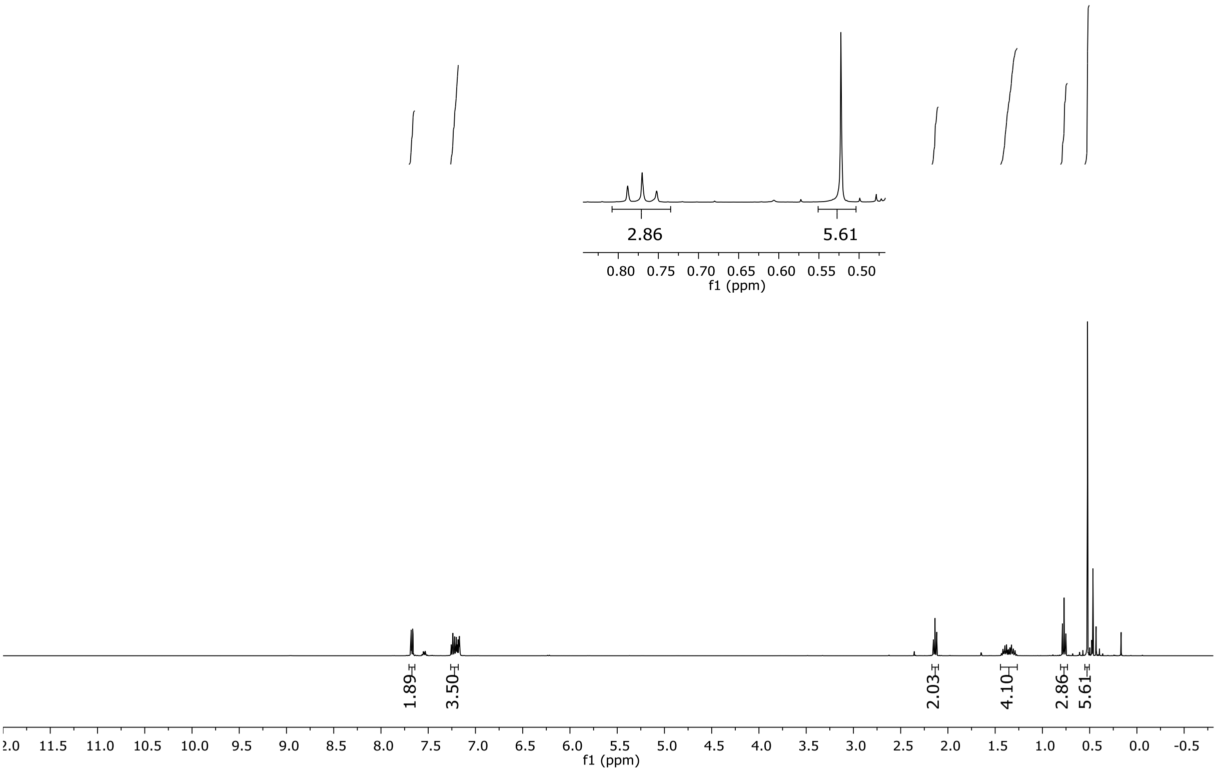

Figure S93. ^{1}H NMR Spectra of hex-1-yn-1-yldimethyl(phenyl)germane (**6m**)

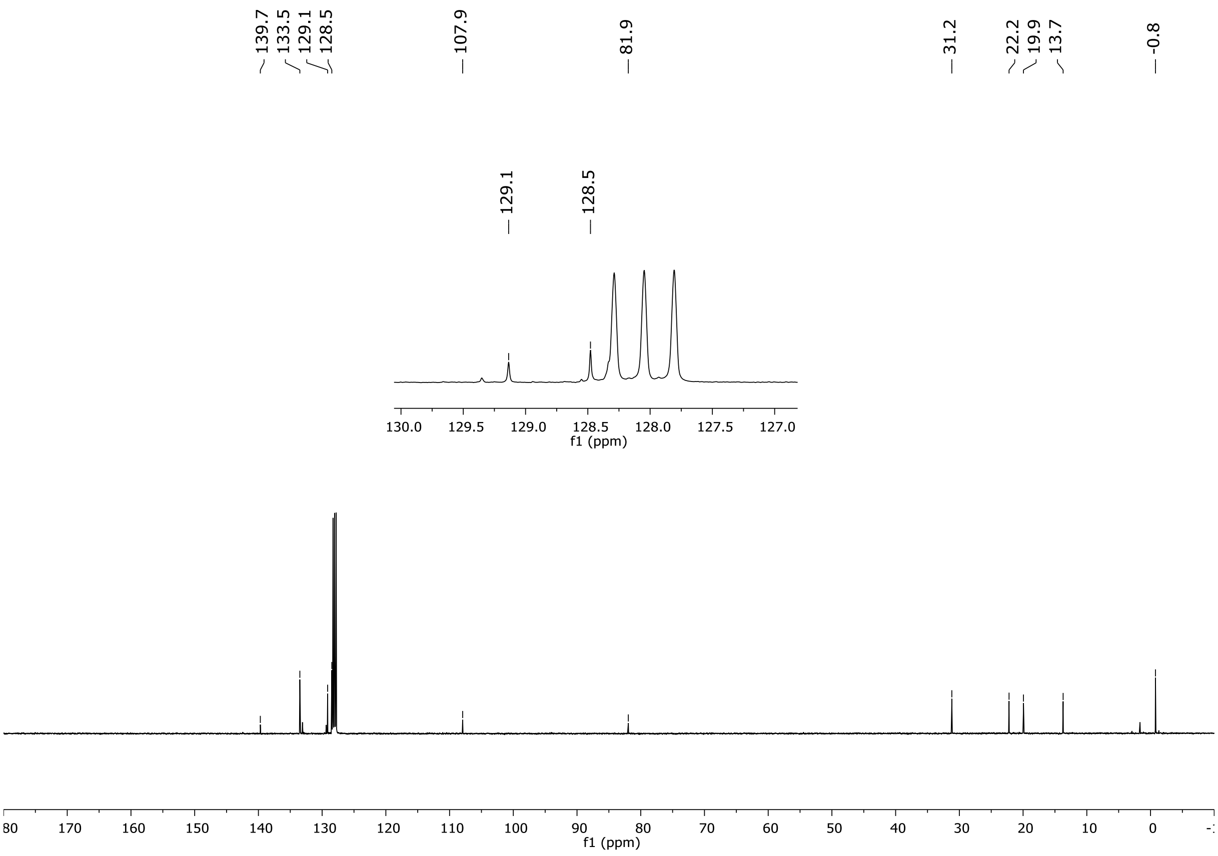

Figure S94. ^{13}C NMR Spectra of hex-1-yn-1-yldimethyl(phenyl)germane (**6m**)

Confirmation of the deprotonation step:

A_ For silanol (tert-butyldimethylsilanol (1 eq.) + KHMDS (1 eq.) in ACN-d3

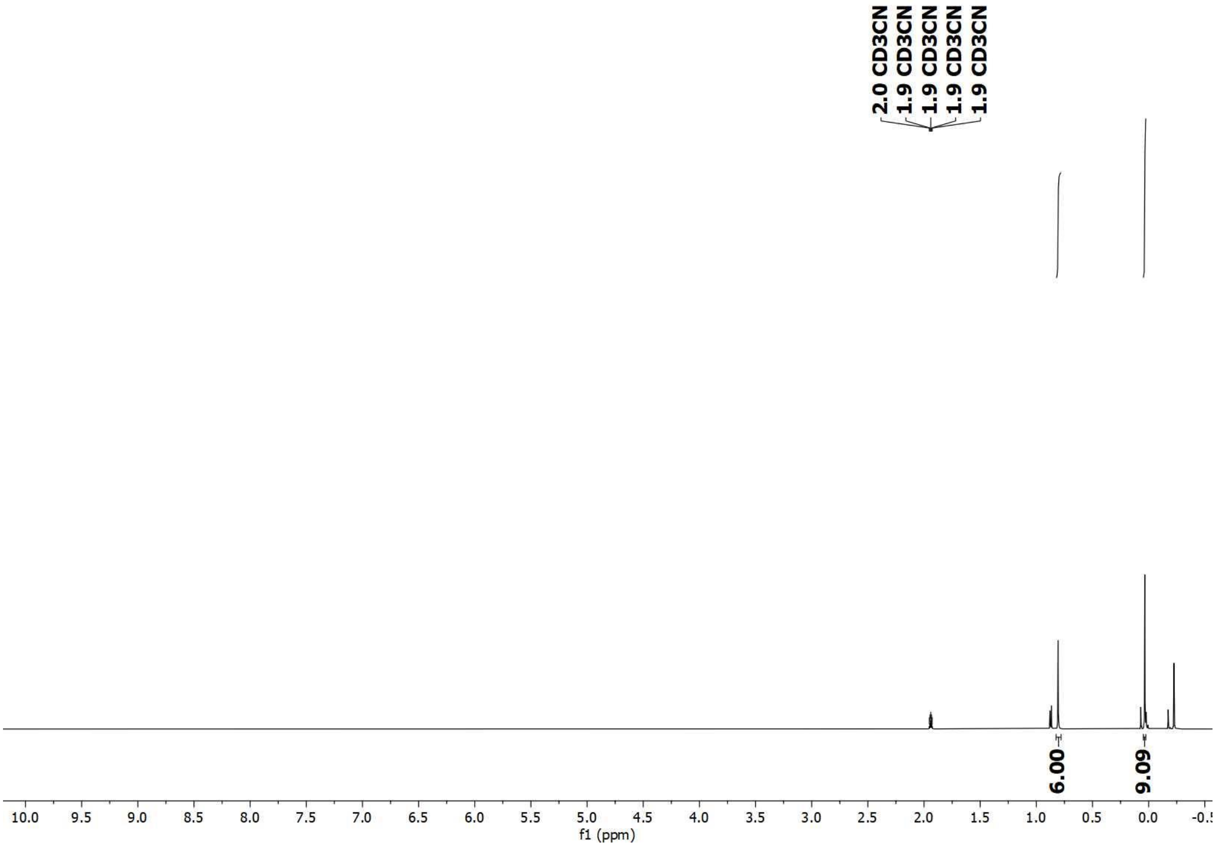

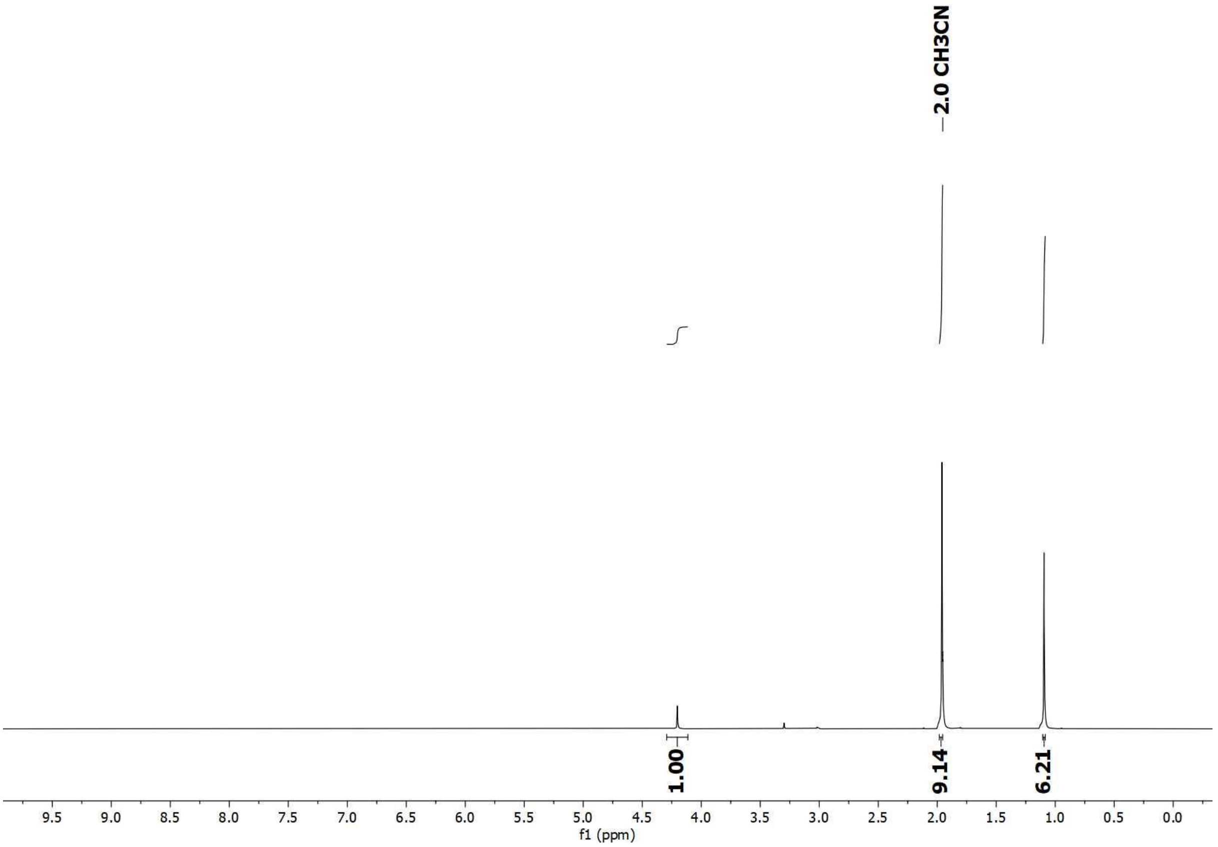

A_ For alkynylgermane (triethylethynylgermane (1 eq.) + KHMDS (1 eq.) in ACN-d3

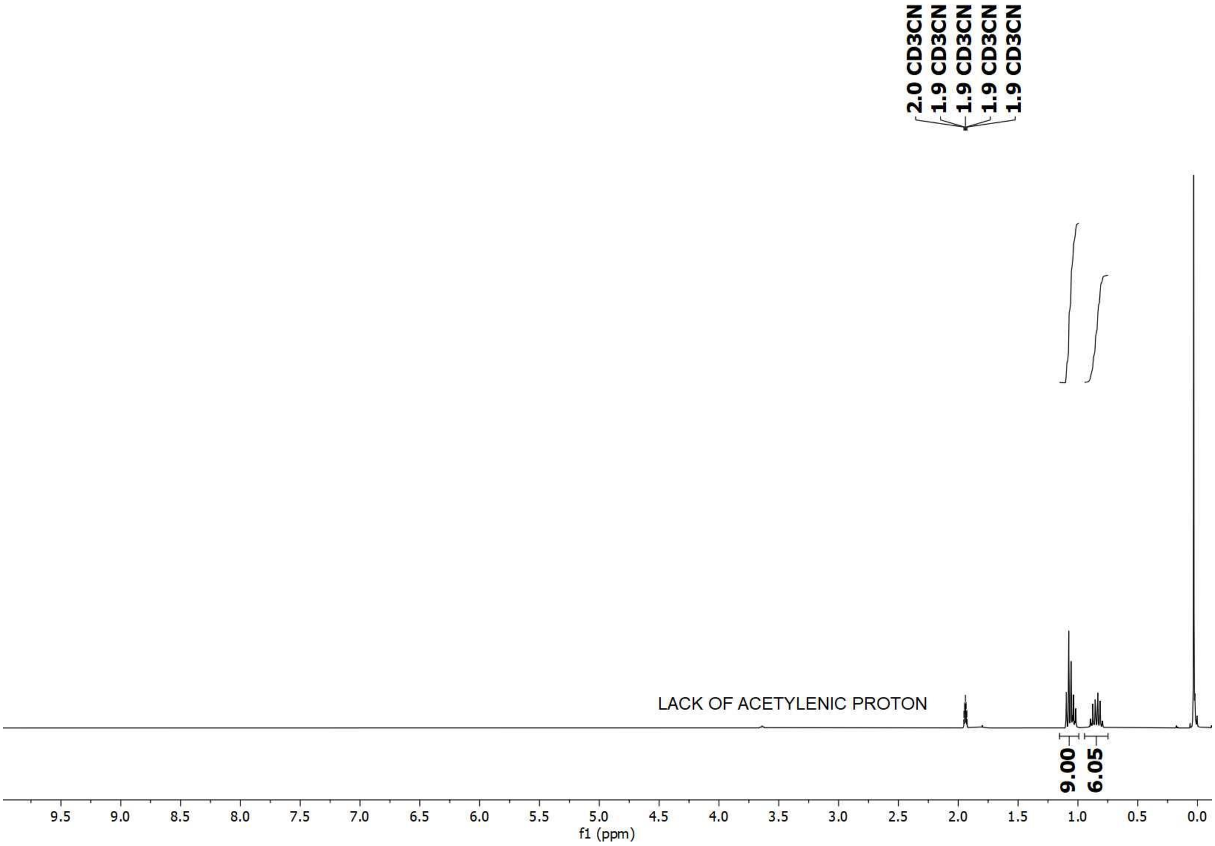

REFERENCES

[1] K. Kuciński and G. Hreczycho *Inorgan. Chim. Acta* **2019**, 490, 261-266, doi:10.1016/j.ica.2019.03.025.

[2] G. Hreczycho, P. Pawluć, and B. Marciniec *New J. Chem.* **2011**, 35, 2743–2746, doi:10.1039/C1NJ20624A.

[3] G. Hreczycho, D. Frąckowiak, P. Pawluć, and B. Marciniec *Tetrahedron Lett.* **2011**, 52, 1, 74-76, doi:10.1016/j.tetlet.2010.10.153.

[4] J. Kaźmierczak and G. Hreczycho *J. Catal.* **2018**, 367, 95-103, doi:10.1016/j.jcat.2018.08.024.

[5] P. Thornton *Sci. Synth.* **2003**, 5, 75–83, doi: 10.1055/sos-sd-005-00063.

[6] B. Marciniec, H. Ławicka, and B. Dudziec *Organometallics* **2007**, 26, 21, 5188–5192, doi.org/10.1021/om700528r.

[7] M. Rzonsowska, B. Woźniak, B. Dudziec, J. Pyziak, I. Kownacki, and B. Marciniec *Eur. J. Inorg. Chem.* **2016**, 339–346, doi.org/10.1002/ejic.201501110.

[8] A. Dahiya and F. Schoenebeck *Org. Lett.* **2022**, 24, 2728−2732, doi.org/10.1021/acs.orglett.2c00840.

[9] A. Dahiya and F. Schoenebeck *ACS Catal.* **2022**, 12, 8048−8054, doi.org/10.1021/acscatal.2c02179.
